# The nature and validity of implicit bias training for health care providers and trainees: A systematic review

**DOI:** 10.1126/sciadv.ado5957

**Published:** 2024-08-14

**Authors:** Nao Hagiwara, Conor Duffy, John Cyrus, Nadia Harika, Ginger S. Watson, Tiffany L. Green

**Affiliations:** ^1^ Department of Public Health Sciences, University of Virginia, Charlottesville, VA 22903, USA.; ^2^ Department of Psychology, Virginia Commonwealth University, Richmond, VA 23284, USA.; ^3^ Research and Education Department, Health Sciences Library, Virginia Commonwealth University, Richmond, VA 23298, USA.; ^4^ Department of Pediatrics, Virginia Commonwealth University, Richmond, VA 23219, USA.; ^5^ Virginia Modeling Analysis & Simulation Center, Old Dominion University, Suffolk, VA 23435, USA.; ^6^ Departments of Population Health Sciences and Obstetrics and Gynecology, University of Wisconsin-Madison, Madison, WI 53726, USA.

## Abstract

The number of health care educational institutions/organizations adopting implicit bias training is growing. Our systematic review of 77 studies (published 1 January 2003 through 21 September 2022) investigated how implicit bias training in health care is designed/delivered and whether gaps in knowledge translation compromised the reliability and validity of the training. The primary training target was race/ethnicity (49.3%); trainings commonly lack specificity on addressing implicit prejudice or stereotyping (67.5%). They involved a combination of hands-on and didactic approaches, lasting an average of 343.15 min, often delivered in a single day (53.2%). Trainings also exhibit translational gaps, diverging from current literature (10 to 67.5%), and lack internal (99.9%), face (93.5%), and external (100%) validity. Implicit bias trainings in health care are characterized by bias in methodological quality and translational gaps, potentially compromising their impacts.

## Teaser

Health care implicit bias training effectiveness is hindered by methodological flaws and gaps in knowledge translation.

## INTRODUCTION

Health care providers’ implicit racial bias (spontaneously activated attitudes and beliefs) is one major factor contributing to racial disparities in the patient care quality (*1*–*3*). Emerging data also suggest other forms of implicit bias (e.g., weight, disability, and sexuality) undermine patients’ health care experiences. Many U.S. educational institutions and organizations responsible for training health care providers have made increasing efforts to address implicit bias. For example, in 2020, the American Medical Association renewed their pledge to combat implicit bias in medicine and announced that they would lobby medical schools to implement implicit bias training (*4*). Legislators have also pushed to mandate implicit bias training for health care providers. Currently, eight states mandate implicit bias training as part of continuing medical education requirements (*5*). Furthermore, the Black Maternal Momnibus Act of 2023 calls for the provision of “funding for grant programs to implement and study consistent bias, racism, and discrimination trainings for all employees in maternity care settings” (*6*, *7*). Efforts to address implicit bias in health care are not limited to the United States. In response to the recent student activism, several medical schools in the United Kingdom made commitments to implement implicit bias training (*8*).

Despite the increasing number of health care educational institutions and organizations adopting implicit bias training, there are no guidelines for the development and implementation of such training. Further, the training content often does not reflect current scientific knowledge about implicit bias and its role in the patient care quality. For example, many implicit bias trainings tend to focus only on the affective component of implicit bias—prejudice. While implicit prejudice is linked consistently to the patient-provider communication quality (*9*–*12*), multiple recent reviews (*2*, *13*, *14*) found little evidence to support a link between provider implicit prejudice and provider treatment recommendations or final decisions. Lastly, there is no evidence these trainings result in long-term behavioral change.

In their call to action, Hagiwara and colleagues (*15*) posited that the development and implementation of effective implicit bias training must be (i) grounded in the knowledge gained through social psychology research on implicit bias and (ii) executed in incremental stages within the Clinical and Translational Science (CTS) framework. The five stages in the CTS range from T0 (basic science research) to T4 (Translation to community) (*16*). They argued any translational gap between the stages drastically attenuates the effectiveness of implicit bias training. Although Hagiwara and colleagues posited that T1 (Translation to humans) to T3 (Translation to practices) are particularly relevant to implicit bias training (*15*), we argue that T4 is a critical stage for ensuring generalizability of training and maximizing its impact. The goal of this systematic review was twofold. First, we investigated how implicit bias training for health care trainees and providers is designed and delivered. Second, we assessed whether the reliability and validity of the implicit bias training were compromised by translational gaps. Recognizing where and what translational gaps exist is essential for improving implicit bias training and ultimately achieving health care equity.

## RESULTS

### Study selection

**Fig. 1.**
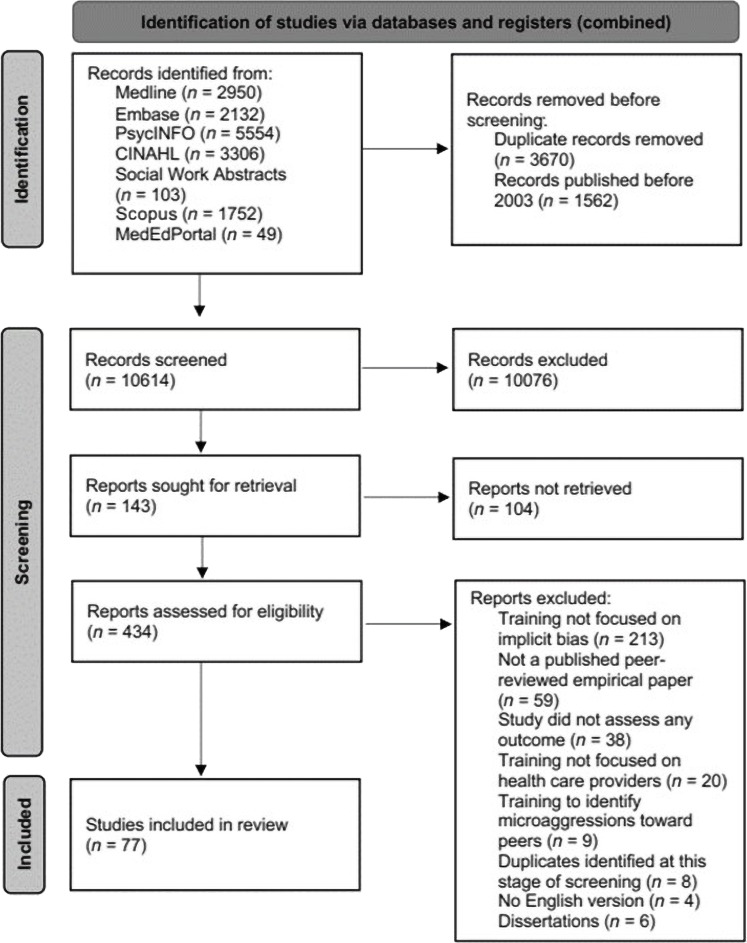
Identification of studies. Preferred Reporting Items for Systematic reviews and Meta-Analyses (PRISMA) diagram illustrating selection and review process of articles related to implicit bias training in health care.

The initial searches generated 14,183 results, which were reduced to 9424 abstracts after removing duplicates and studies published before 2003. During the abstract screening, 8950 records were excluded because the abstracts did not mention implicit bias training, and 76 records were excluded because they were conference abstracts. We screened 398 full-text articles and excluded 336 studies that did not meet all inclusion criteria, resulting in 62 studies. The 1-year follow-up searches generated 1663 results, which were reduced to 1190 without duplicates. One thousand one hundred twenty-six and 28 manuscripts were removed because their abstracts did not mention implicit bias or they were conference abstracts, respectively. Twenty-one studies not meeting all inclusion criteria were removed during full-text screening, resulting in 15 studies. The current review included 77 studies (Fig. 1 and table S2).

### Study characteristics

**Fig. 2.**
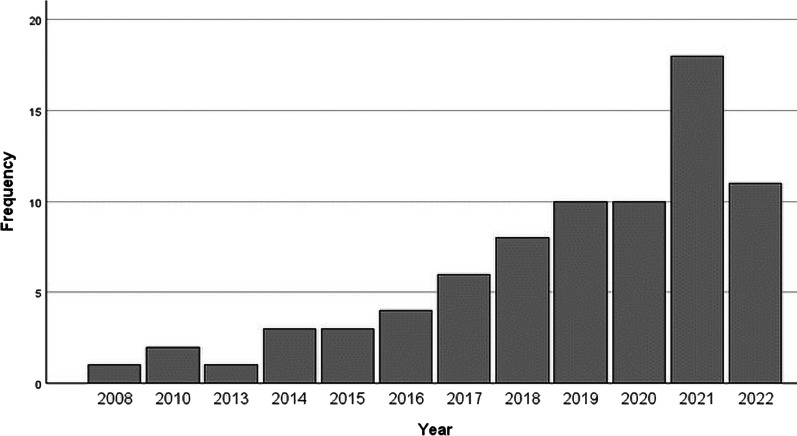
Published studies on implicit bias training. A number of empirical studies on implicit bias training published between January 2003 and September 2022 by year.

The first study was published in 2008, and the number of published studies has rapidly increased over years (Fig. 2).

### Study design

The most common research design used was quantitative (*n* = 38, 49.4%), followed by mixed methods (*n* = 28, 36.4%) and qualitative designs (*n* = 11, 14.3%). In 35 studies (45.5%), implicit bias training was delivered as part of the regular curriculum; in the remaining 42 studies (54.5%), training was delivered in addition to the regular curriculum. While implicit bias was the main training goal in 41 studies (53.2%), it was part of larger goals in 35 studies (45.5%; *n* = 1 unclear). Some common larger goals included cultural sensitivity and/or competency, DEI (diversity, equity, and inclusion), antiracism, and health disparities.

### Participants

Sample sizes ranged from 12 to 1250, representing all professional statuses (i.e., students, residents, fellows, and faculty/practicing clinicians) in 16 clinical specialties.

### Risk of bias in studies

The mean score of the Medical Education Research Quality Instrument (MERSQI) (sum of 10 items) of 66 quantitative (table S3) and mixed methods studies (table S4) was 8.95 (SD = 2.85; range, 5.5 to 16). Only 14 studies (21.2%) were considered low risk of bias. In addition, only four studies (6.0%) demonstrated full validation of the assessment measures.

The mean score of the Critical Appraisal Skills Programme (CASP) (percentage of “yes” across 10 items) of 39 qualitative (table S5) and mixed-methods studies was 45.90% (SD = 28.54%; range, 0 to 90%). Only 11 studies (28.2%) were considered low risk of bias. Notably, less than half of the studies (48.7%) clearly defined their qualitative study aims. Consequently, we were unable to determine the appropriateness of the qualitative methodological approach, research design, and the theoretical underpinnings for those studies.

### Results of individual studies and syntheses

Data S1 presents characteristics of individual implicit bias trainings reported in the 77 studies.

### Training characteristics

Table 1 provides the summary of training characteristics stratified by low versus moderate to high bias risk. Overall, the bias component, the targets of bias, and the training structure (format, duration, and frequency) were similar between the two groups. However, there were several notable differences.

**Table 1.** Summary of training characteristics stratified by the levels of study bias risk (low versus moderate to high).

Training characteristics	Low bias risk (*n* = 22)	Moderate to high bias risk (*n* = 55)
**Component of implicit bias**		
Implicit prejudice	3 (13.6%)	5 (9.1%)
Implicit stereotyping	2 (9.1%)	0
Both	5 (22.7%)	10 (18.2%)
Unclear	12 (54.5%)	40 (72.7%)
**Target of bias**		
Race/ethnicity	9 (40.9%)	29 (52.7%)
Weight	5 (22.7%)	5 (9.1%)
Gender	2 (9.1%)	7 (12.7%)
Sexual and gender minorities	2 (9.1%)	7 (12.7%)
Socioeconomic status	0	5 (9.1%)
Other	Immigration status, disability, culture, mental health, skin tone	Immigration status, disability, culture, mental health, substance use disorders, age, religion, power
Unclear	3 (13.6%)	15 (27.3%)
**Training format**		
A combination	14 (63.6%)	44 (80.0%)
Hands-on activities only	4 (18.2%)	10 (18.1%)
Didactic presentations only	3 (13.6%)	0
Unclear	1 (4.5%)	1 (1.8%)
**Training duration** ^*^	Range: 34–3030 min *M* = 409.31 (*SD* = 753.05)	Range: 50–1680 min *M* = 308.51 (*SD* = 379.71)
Unclear	6 (27.3%)	18 (32.7%)
**Training frequency**		
A single day	12 (54.5%)	29 (52.7%)
Multiple days	9 (40.9%)	23 (41.8%)
Unclear	1 (4.5%)	3 (5.5%)

*When studies reported ranges for training duration, we used the corresponding middle values to compute means and SDs.

#### Bias component

The proportion of studies that did not specify the component of implicit bias (i.e., prejudice, stereotyping, or both) that their training aimed to address was higher among moderate to high (72.7%) than low bias risk studies (54.5%). In addition, only two trainings reported in the low bias risk studies addressed implicit stereotyping only (9.1%).

#### Bias target

The proportion of studies that did not specify the target of bias was higher in the moderate to high (27.3%) than in the low bias risk group (13.6%). The four most commonly addressed targets were the same between the two groups (i.e., race/ethnicity, weight, gender, and sexual orientation/gender identity). Some trainings reported in moderate to high bias risk studies also focused on socioeconomic status (*n* = 5, 9.1%) and gave participants an option to select one from multiple options (*n* = 1, 1.8%). It should be noted that the numbers for the bias target do not add up to 100% because some training programs targeted multiple groups (13.6 and 16.4% of low versus moderate to high bias risk studies, respectively).

#### Training structure

All three aspects of training structure (format, duration, and frequency) were similar between low and moderate to high bias risk studies: Trainings typically involved a combination of hands-on activities and didactic presentations (75.3%), which on average lasted 343.15 min (SD = 519.45) and were delivered in a single day (53.2%). However, there were three notable differences between the two groups. First, trainings reported in low bias risk studies tended to last longer (*M* = 409.31) than moderate to high bias risk studies (*M* = 308.51). Second, there were more trainings reported in moderate to high bias risk studies that used a combination of hands-on activities and didactic presentations (80.0%) than low bias risk studies (63.6%). Last, all three trainings that used didactic presentations alone were low bias risk studies.

### Translational gaps

Table 2 provides the summary of translational gaps across stages T1 through T4 stratified by the levels of study bias risk.

**Table 2.** Summary of translational gaps across stages T1 through T4 stratified by the levels of study bias risk (low versus moderate to high).

Translational gap	Low bias risk (*n* = 22)	Moderate to high bias risk (*n* = 55)
**Between T1 and T2**		
Specified bias component	10 (45.5%)	15 (27.3%)
Specified bias target (particularly when addressing stereotyping)	7 (31.8%)	8 (14.5%)
Consistent with the evidence-based “habit breaking” strategies		
Hands-on practices	18 (81.8%)	54 (98.2%)
Training across multiple days	9 (40.9%)	23 (41.8%)
**Between T2 and T3**		
Evidence for internal validity		
Some evidence (one group pre-and-post change)	8 (36.4%)	22 (40.0%)
Some evidence (at least one comparison group, no randomization)	2 (9.1%)	1 (1.8%)
Strong evidence (at least one comparison group, randomization)	7 (31.8%)	0
Evidence for face validity*		
Self-reported changes in attitudes and/or beliefs	2 (9.1%)	7 (12.7%)
Assessed changes in explicit attitudes and/or beliefs	6 (27.3%)	38 (69.1%)
Assessed changes in implicit attitudes and/or beliefs	11 (50.0%)	8 (14.5%)
Assessed changes in behaviors	3 (13.6%)	2 (3.6%)
Assessed changes in patient outcomes	0	0
**Between T3 and T4**		
Evidence for external validity		
Some evidence (one sample consisting of individuals from multiple professional statuses and/or fields)	5 (22.7%)	16 (29.1%)
Strong evidence (multiple samples coming from different institutions/events)	0	0

*When studies used multiple assessment tools, we selected the highest validity tool as the evidence for face validity.

#### T1-T2 translational gap

A gap between T1 and T2 reflects inconsistencies between the scientific evidence of implicit bias and how implicit bias training is designed. As noted in the introduction, implicit prejudice predicts the quality of patient-provider communication but not the quality of treatment recommendations (*2*, *13*, *14*). Our review revealed that 67.5% of studies overall failed to specify which component of bias their trainings are designed to address despite that more low (45.5%) than moderate to high bias risk studies (27.3%) specified the bias component. Further, there were more low bias risk studies (31.8%) than moderate to high bias risk studies (14.5%) that reported the target of bias when their trainings were designed to address stereotyping.

Two important elements of successful behavioral change are (i) learning specific strategies to replace old behaviors and (ii) the opportunity to practice the strategies repeatedly over time (*17*). As proxy measures, we looked at whether implicit bias training used specific hands-on activities and whether the trainings were delivered across multiple days (assuming that attendees are more likely to be given opportunities to rehearse what they learned in earlier sessions). While almost all training programs involved some sort of hands-on activities (81.8 and 98.2% low versus moderate to high bias risk studies, respectively), less than half of the programs were delivered across multiple days (40.9 and 41.8% low versus moderate to high bias risk studies, respectively).

#### T2-T3 translational gaps

Gaps between T2 and T3 are reflected in how researchers assessed (i.e., face validity) and tested the effectiveness (i.e., internal validity) of implicit bias training. First, the majority of the low bias risk studies examined changes in implicit attitudes and/or beliefs (50.0%), whereas the majority of the moderate to high bias risk studies examined changes in explicit attitudes and/or beliefs (69.1%). More critically, the intended goals of implicit bias training are improved provider behaviors (i.e., communication behaviors, clinical recommendations, or both) and/or improved patient outcomes (e.g., increased reports in care satisfaction, improved clinical outcomes, increased subsequent health care utilization). However, only five studies (6.5%) examined behavioral changes among providers, and no study examined patient outcomes, indicating low face validity overall.

Second, the only study design that enables researchers to conclude, with confidence, that their trainings mitigate implicit bias is a randomized clinical trial. Only seven studies (9.1%), which were all low bias risk studies, used a randomized clinical trial to test the internal validity of their trainings. Only three of the seven studies found evidence for a significant change in providers’/trainees’ attitudes or behaviors.

#### T3-T4 translational gap

A gap between T3 and T4 reflects the lack of rigorous testing of external validity. No studies included in the current review tested the external validity of their implicit bias trainings, although some studies (22.7% of low bias risk studies and 29.1% of moderate to high bias risk studies) used a sample that consisted of individuals from different professional statuses (e.g., students, residents, and faculty) and/or health care fields (e.g., medicine, nursing, and dentistry).

## DISCUSSION

There are several systematic reviews that examined related topics, such as the prevalence of implicit bias in health care professionals (*3*, *18*), consequences of provider implicit bias (*2*, *13*, *19*), and the effectiveness of interventions in reducing implicit bias in general (*20*) and in health care specifically (*21*). However, this systematic review differs from prior reviews in that it addresses the question of why implicit bias training may or may not be effective. Specifically, we formally assessed the reliability and validity of 77 studies over a 20-year period to understand whether and to what extent scholars have developed evidence-based implicit bias training with the broad potential to improve patient outcomes. Participants included students, residents, fellows, and faculty/practicing clinicians across 16 clinical specialties. The most common targets of implicit bias addressed in these trainings were race/ethnicity, weight, gender, and sexual orientation.

Our findings indicate that the number of implicit bias trainings has rapidly increased over time and that organizations are beginning to integrate this programming into standard clinical curricula. However, we found that implicit bias training in health care settings is characterized by bias in methodological quality (as assessed with MERSQI and CASP) and several translational gaps, which likely compromise their potential impacts.

First, few of these studies are rigorously grounded in theoretical evidence (T1-T2 gaps). The most common example of this is the failure to identify the component of implicit bias being addressed in the training. Failing to identify the relevant component likely results in a misalignment between training and desired outcomes (improved patient-provider communication and/or treatment recommendations). Further, even among studies that specified components of bias, the majority failed to tailor the material appropriately. For example, research has established that differential endorsement of explicit stereotypes (e.g., Black people being more prone to opioid addiction) contributes to disparities in treatment recommendations for Black patients versus white patients (*22*, *23*). It is likely the case (although it still needs to be tested empirically) that differential endorsement of implicit stereotypes also contributes to racial disparities in treatment recommendations. Thus, it is important for researchers to specify which group-based stereotypes and what resulting treatment recommendations the training is designed to address.

We found mixed evidence of best teaching practices in training delivery. On one hand, almost all implicit bias trainings used a combination of hands-on activities and didactic presentations. This is consistent with findings from prior research suggesting that mitigating implicit bias requires both improvements in health care providers’ understanding of implicit bias and awareness of their own bias (likely achieved with didactic presentations) and learning and practicing concrete strategies (likely achieved with hands-on activities). While the training format does not always dictate learning outcomes, more interactive learning activities typically lead to higher-level learning outcomes and more consistent transfer of training to the work environment. On the other hand, however, most training is delivered at a single time point, lasting less than 6 hours on average. While this likely reflects the constraints imposed by the current health care system and medical education (e.g., competing priorities for time, financial costs of taking providers out of clinical care for clinics and hospitals, and costs of taking providers out of clinical care for patients), these findings suggest that attendees were unlikely to have enough opportunities to practice newly learned strategies to mitigate their implicit bias. This further provides evidence that implicit bias training aimed solely at reducing providers’ implicit bias is unlikely to be effective or even realistic in mitigating the negative health care consequences of implicit bias within the current health care system (T3-T1 gaps).

Our review also revealed that only a small number of studies used best research practices that allowed the authors to rigorously assess the efficacy of the implicit bias training (T2-T3 gaps). While the goal of implicit bias training is ultimately to improve provider behaviors and/or patient outcomes, most trainings focused on changing attitudes or beliefs alone. Relatedly, variance in the methodological quality of studies is an important and likely unappreciated barrier to progress in this area. Few studies used validated measures and rigorous empirical approaches such as randomized designs to test efficacy (see tables S3 to S5). We strongly urge that investigators use relevant tools to assess the methodological quality of trainings before testing them at the organizational level. Lastly, given that none of the studies in our review tested the external validity of their programs (T3-T4 gaps), we have no evidence of training effectiveness more broadly.

The current findings must be interpreted with the following limitations with the evidence. As reflected in the number of items coded as “unclear,” many studies failed to provide necessary information to code each item included in the current review. It is unclear whether the information was not addressed in the actual implicit bias trainings or authors simply failed to report them in their publications. Relatedly, only a small number of studies were determined to have low bias risk. Consequently, the stratified results syntheses were based on a highly unbalanced number of studies between low and moderate to high bias.

There are also limitations with the review processes that might have potentially affected the interpretation of the results. First, some of the questions in the MERSQI rely on reviewers’ subjective judgments. For example, a binary rating (yes/no) of “appropriateness of analysis” heavily depends on reviewers’ knowledge. In addition, there is a lack of clarity in the literature over which quantified CASP scores indicate high, moderate, and low bias (*24*). Despite these limitations, our approach to the assessment of study bias risk is consistent with the current paradigm and enables us to explore the strengths and weaknesses of both individual studies and the state of research.

Second, the report and interpretation of the implicit bias trainings were based on details provided in the publication, which were often sparse. Very few studies provided sufficient information to verify the instructional methods or content covered. Lack of materials sharing limits replication and reproducibility of the study findings, as well as interpretation and generalizability in this review.

Does implicit bias training work? Should health care educational institutions and organizations as well as the government continue to spend their efforts on it? Our conclusion based on the findings from the current systematic review is it is premature to answer these questions. There is little scientific evidence to support that implicit bias training improves the quality of patient care, and this could be due to three reasons. First, some trainings may actually improve patient care, but they simply did not use proper outcome assessments (i.e., T2-T3 translational gaps). Second, implicit bias trainings just do not work because of translational gaps between T1 and T2. Third, even well-designed implicit bias training that is validated outside the health care context may not be effective in reducing provider implicit bias because of the gaps between T3 back to T1. It is critical for health care educational institutions and organizations to both expand the objectives of implicit bias training and (re)evaluate their implicit bias trainings by using the CTS framework before they (further) devote their time and resources into implicit bias training. Similarly, T3-T4 translational gaps found in all reviewed studies suggest that rigorous testing of external validity of the implicit bias trainings currently used in eight states is urgently needed before more states start to mandate such training.

## MATERIALS AND METHODS

### Eligibility criteria

We defined implicit bias training for health care trainees/providers as the action of teaching trainees/providers the knowledge and skills to either reduce their negative attitudes toward and/or beliefs about a certain social group or mitigate the health care consequences of their bias(es). This is not to imply that implicit bias training programs with other objectives, such as promoting DEI within the health care system (*25*, *26*) or reducing interprofessional conflicts (*27*, *28*), are not important. However, the training designs, contents, and the assessments of the reliability and validity of the implicit bias training would vary depending on the training objectives. Therefore, this systematic review focused on implicit bias training aimed primarily at reducing disparities in the quality of health care among patients from diverse social groups. Given this focus, we limited our review to training targeting health care trainees/providers with direct patient contact. We also excluded undergraduates pursuing careers in health care because they generally do not have direct patient contact.

We excluded studies from the review if they did not clearly state addressing implicit bias was one of their goals (simply mentioning implicit bias as one training component was insufficient). We also limited our review to published empirical studies or education interventions written in English. We excluded published theses/dissertations and conference abstracts because they generally do not undergo rigorous review processes. Last, we excluded studies published before 2003 to correspond with the publication of the Institute of Medicine’s seminal report “Unequal Treatment” (*1*).

### Information sources and search strategy

We conducted the initial search on 1 September 2021 and the 1-year follow-up search on 21 September 2022: Medline (Ovid), Embase (Ovid), PsycINFO (Proquest), Cumulative Index to Nursing and Allied Health Literature (CINAHL) [Elton B. Stephens Company (EBSCO)], Social Work Abstracts (EBSCO), Scopus (Elsevier), and MedEdPortal. Searches included terms, phrases, and controlled vocabulary related to the concepts of implicit bias and health care professionals, including practitioners, trainees, and levels of training. See table S1 for full search strategies.

### Selection process

After removing duplicates, two reviewers coded study abstracts independently as “include,” “exclude,” or “maybe” using Rayyan (*29*). Coding discrepancies were discussed and solved by the two reviewers first and with all coauthors where necessary. Next, two reviewers independently read full texts of “include” or “maybe” studies and determined whether the studies met all eligibility criteria. Once again, conflicts were first resolved by the two reviewers and then with the coauthors when necessary.

### Data collection process

Two reviewers independently extracted information for data synthesis that included bias component (prejudice, stereotyping, or both), sample size, sample professional characteristics (trainee/provider status and health care fields), training structure (format, duration, and frequency), and outcomes. Primary outcomes were broadly categorized as follows: changes in (i) explicit bias; (ii) implicit bias; (iii) trainee/provider behaviors; and (iv) patient outcomes. We also included self-reflections (e.g., self-awareness of bias, motivation to reduce bias, and intention to improve patient care) as secondary outcomes. Discrepancies were resolved through discussion, first between the two reviewers and then with a third reviewer where necessary.

### Study risk of bias assessment

Two reviewers independently assessed risk of bias of quantitative and qualitative studies by using the MERSQI (*30*) and the CASP checklist (*31*), respectively. Discrepancies were resolved through discussion first between the two reviewers and then with a third reviewer where necessary. We computed the total score of 10 items in MERSQI and the percentage of “yes” across 10 items in CASP. Last, we used the cutoff scores of MERSQI ≥12 (*32*) and CASP ≥66.7% (*24*) to define “low bias” (versus “moderate to high bias”).

### Synthesis methods

We anticipated a high level of heterogeneity in the findings; thus, we decided to provide a narrative synthesis of the findings. To account for this variability, we created a comprehensive tabulation of study characteristics, structured around intended goals of training, the training content, bias component, and outcomes. We also summarized internal validity (the extent to which cause-and-effect relationships between training and outcomes are established with confidence), face validity (the degree to which outcomes assessed in a study is consistent with the intended goal of the study), and external validity (the extent to which training produces the same outcomes in different populations and settings). The findings were stratified according to bias risk using subgroup analysis. The procedure was preregistered with PROSPERO (CRD42021270641).

## Supplementary Materials

### The PDF file includes:

Tables S1, S3 to S5 Legend for table S2 Legend for data S1 References

## References

[1] Institute of Medicine (US) Committee on Understanding and Eliminating Racial and Ethnic Disparities in Health Care, *Unequal Treatment: Confronting Racial and Ethnic Disparities in Health Care* (National Academies Press, 2003); www.ncbi.nlm.nih.gov/books/NBK220358/. 25032386

[2] I. W. Maina, T. D. Belton, S. Ginzberg, A. Singh, T. J. Johnson, A decade of studying implicit racial/ethnic bias in healthcare providers using the implicit association test. Soc. Sci. Med. 199, 219–229 (2018). 28532892 10.1016/j.socscimed.2017.05.009

[3] C. FitzGerald, S. Hurst, Implicit bias in healthcare professionals: A systematic review. BMC Med. Ethics 18, 19–18 (2017). 28249596 10.1186/s12910-017-0179-8 PMC5333436

[4] S. Heath, AMA joins industry efforts against medical racism, implicit bias (Patient Engagement Hit, 2023); https://patientengagementhit.com/news/ama-joins-industry-efforts-against-medical-racism-implicit-bias.

[5] L. A. Cooper, S. Saha, M. van Ryn, Mandated implicit bias training for health professionals—A step toward equity in health care. JAMA Health Forum 3, e223250 (2022). 36218984 10.1001/jamahealthforum.2022.3250

[6] U.S. Senator Cory Booker of New Jersey, Booker, Underwood, Adams reintroduce the bicameral Momnibus Act to end America’s maternal health crisis (2023); www.booker.senate.gov/news/press/booker-underwood-adams-reintroduce-the-bicameral-momnibus-act-to-end-americas-maternal-health-crisis.

[7] 118th Congress (2023-2024), *H.R.3305 - Black Maternal Health Momnibus Act* (2023); www.congress.gov/bill/118th-congress/house-bill/3305/text.

[8] P. Papinemi, S. Filson, T. Harrison, M. McIntosh, *Adopting an anti-racist medical curriculum* (The BMJ, 2021); https://blogs.bmj.com/bmj/2021/02/19/adopting-an-anti-racist-medical-curriculum/.

[9] N. Hagiwara, L. A. Penner, R. Gonzalez, S. Eggly, J. F. Dovidio, S. L. Gaertner, T. West, T. L. Albrecht, Racial attitudes, physician-patient talk time ratio, and adherence in racially discordant medical interactions. Soc. Sci. Med. 87, 123–131 (2013). 23631787 10.1016/j.socscimed.2013.03.016 PMC3677202

[10] N. Hagiwara, R. B. Slatcher, S. Eggly, L. A. Penner, Physician racial bias and word use during racially discordant medical interactions. Health Commun. 32, 401–408 (2017). 27309596 10.1080/10410236.2016.1138389 PMC5161737

[11] L. A. Penner, J. F. Dovidio, R. Gonzalez, T. L. Albrecht, R. Chapman, T. Foster, F. W. K. Harper, N. Hagiwara, L. M. Hamel, A. F. Shields, S. Gadgeel, M. S. Simon, J. G. Griggs, S. Eggly, The effects of oncologist implicit racial bias in racially discordant oncology interactions. J. Clin. Oncol. 34, 2874–2880 (2016). 27325865 10.1200/JCO.2015.66.3658 PMC5012663

[12] L. A. Cooper, D. L. Roter, K. A. Carson, M. C. Beach, J. A. Sabin, A. G. Greenwald, T. S. Inui, The associations of clinicians’ implicit attitudes about race with medical visit communication and patient ratings of interpersonal care. Am. J. Public Health 102, 979–987 (2012). 22420787 10.2105/AJPH.2011.300558 PMC3483913

[13] E. Dehon, N. Weiss, J. Jones, W. Faulconer, E. Hinton, S. Sterling, A systematic review of the impact of physician implicit racial bias on clinical decision making. Acad. Emerg. Med. 24, 895–904 (2017). 28472533 10.1111/acem.13214

[14] N. Hagiwara, J. F. Dovidio, J. Stone, L. A. Penner, Applied racial/ethnic healthcare disparities research using implicit measures. Soc. Cogn. 38, s68–s97 (2020). 34103783 10.1521/soco.2020.38.supp.s68 PMC8183978

[15] N. Hagiwara, F. W. Kron, M. W. Scerbo, G. S. Watson, A call for grounding implicit bias training in clinical and translational frameworks. Lancet 395, 1457–1460 (2020). 32359460 10.1016/S0140-6736(20)30846-1 PMC7265967

[16] S. H. Woolf, The meaning of translational research and why it matters. JAMA 299, 211–213 (2008). 18182604 10.1001/jama.2007.26

[17] C. A. Zestcott, I. V. Blair, J. Stone, Examining the presence, consequences, and reduction of implicit bias in health care: A narrative review. Group Process. Intergroup Relat. 19, 528–542 (2016). 27547105 10.1177/1368430216642029 PMC4990077

[18] B. Ahadinezhad, O. Khosravizadeh, A. Maleki, A. Hashtroodi, Implicit racial bias among medical graduates and students by an IAT measure: A systematic review and meta-analysis. Ir. J. Med. Sci. 191, 1941–1949 (2022). 34495481 10.1007/s11845-021-02756-3

[19] W. J. Hall, M. V. Chapman, K. M. Lee, Y. M. Merino, T. W. Thomas, B. K. Payne, E. Eng, S. H. Day, T. Coyne-Beasley, Implicit racial/ethnic bias among health care professionals and its influence on health care outcomes: A systematic review. Am. J. Public Health 105, e60–e76 (2015). 10.2105/AJPH.2015.302903 PMC4638275 26469668

[20] C. FitzGerald, A. Martin, D. Berner, S. Hurst, Interventions designed to reduce implicit prejudices and implicit stereotypes in real world contexts: A systematic review. BMC Psychol. 7, 29 (2019). 31097028 10.1186/s40359-019-0299-7 PMC6524213

[21] M. B. Vela, A. I. Erondu, N. A. Smith, M. E. Peek, J. N. Woodruff, M. H. Chin, Eliminating explicit and implicit biases in health care: Evidence and research needs. Annu. Rev. Public Health 43, 477–501 (2022). 35020445 10.1146/annurev-publhealth-052620-103528 PMC9172268

[22] S. K. Calabrese, V. A. Earnshaw, K. Underhill, N. B. Hansen, J. F. Dovidio, The impact of patient race on clinical decisions related to prescribing HIV pre-exposure prophylaxis (PrEP): Assumptions about sexual risk compensation and implications for access. AIDS Behav. 18, 226–240 (2014). 24366572 10.1007/s10461-013-0675-x PMC3983275

[23] K. M. Hoffman, S. Trawalter, J. R. Axt, M. N. Oliver, Racial bias in pain assessment and treatment recommendations, and false beliefs about biological differences between blacks and whites. Proc. Natl. Acad. Sci. U.S.A. 113, 4296–4301 (2016). 27044069 10.1073/pnas.1516047113 PMC4843483

[24] H. A. Long, D. P. French, J. M. Brooks, Optimising the value of the critical appraisal skills programme (CASP) tool for quality appraisal in qualitative evidence synthesis. Res. Methods Med. Health Sci. 1, 31–42 (2020).

[25] S. M. Cheng, C. C. McKinney, A. Hurtado-de-Mendoza, S. Chan, K. D. Graves, Confidence, connection & collaboration: Creating a scalable bias reduction improvement coaching train-the-trainer program to mitigate implicit bias across a medical center. Teach. Learn. Med. 36, 381–398 (2023). 37074228 10.1080/10401334.2023.2201289

[26] C. Okorie-Awé, S. Y. Crawford, L. K. Sharp, B. U. Jaki, M. D. Kachlic, A faculty and staff workshop on microaggression and implicit bias: Knowledge and awareness of student, faculty, and staff experiences. Curr. Pharm. Teach. Learn. 13, 1200–1209 (2021). 34330399 10.1016/j.cptl.2021.06.031

[27] H. J. Braun, P. S. O’Sullivan, M. N. Dusch, S. Antrum, N. L. Ascher, Improving interprofessional collaboration: Evaluation of implicit attitudes in the surgeon-nurse relationship. Int. J. Surg. 13, 175–179 (2015). 25497005 10.1016/j.ijsu.2014.11.032

[28] J. Sukhera, K. Bertram, S. Hendrikx, M. S. Chisolm, J. Perzhinsky, E. Kennedy, L. Lingard, M. Goldszmidt, Exploring implicit influences on interprofessional collaboration: A scoping review. J. Interprof. Care 36, 716–724 (2022). 34602007 10.1080/13561820.2021.1979946

[29] Rayyan, “Rayyan - AI powered tool for systematic literature reviews” (2021) 36; www.rayyan.ai/.

[30] D. A. Reed, D. A. Cook, T. J. Beckman, R. B. Levine, D. E. Kern, S. M. Wright, Association between funding and quality of published medical education research. JAMA 298, 1002–1009 (2007). 17785645 10.1001/jama.298.9.1002

[31] Critical Appraisal Skills Programme, “CASP - Critical Appraisal Skills Programme”; https://casp-uk.net/casp-tools-checklists/.

[32] D. A. Cook, R. Hatala, R. Brydges, B. Zendejas, J. H. Szostek, A. T. Wang, P. J. Erwin, S. J. Hamstra, Technology-enhanced simulation for health professions education: A systematic review and meta-analysis. JAMA 306, 978–988 (2011). 21900138 10.1001/jama.2011.1234

[33] K. W. Bartlett, P. Strelitz, J. Hawley, R. Sloane, B. B. Staples, Impact of small-group workshop on resident preparedness to provide culturally competent care. Acad. Pediatr. 15, e1–e2 (2015).

[34] R. Bernstein, L. Ruffalo, D. Bower, A multielement community medicine curriculum for the family medicine clerkship. MedEdPORTAL 12, 10417 (2016). 31008197 10.15766/mep_2374-8265.10417 PMC6464429

[35] M. V. Chapman, W. J. Hall, K. Lee, R. Colby, T. Coyne-Beasley, S. Day, E. Eng, A. F. Lightfoot, Y. Merino, F. M. Siman, T. Thomas, K. Thatcher, K. Payne, Making a difference in medical trainees’ attitudes toward Latino patients: A pilot study of an intervention to modify implicit and explicit attitudes. Soc. Sci. Med. 199, 202–208 (2018). 28532893 10.1016/j.socscimed.2017.05.013 PMC5714690

[36] L. Corsino, K. Railey, K. Brooks, D. Ostrovsky, S. O. Pinheiro, A. McGhan-Johnson, B. I. Padilla, The impact of racial bias in patient care and medical education: Let’s focus on the educator. MedEdPORTAL 17, 11183 (2021). 34557589 10.15766/mep_2374-8265.11183 PMC8410857

[37] M. DallaPiazza, M. Padilla-Register, M. Dwarakanath, E. Obamedo, J. Hill, M. L. Soto-Greene, Exploring racism and health: An intensive interactive session for medical students. MedEdPORTAL 14, 10783 (2018). 30800983 10.15766/mep_2374-8265.10783 PMC6354798

[38] D. L. F. Davis, D. Tran-Taylor, E. Imbert, J. O. Wong, C. L. Chou, Start the way you want to finish: An intensive diversity, equity, inclusion orientation curriculum in undergraduate medical education. J. Med. Educ. Curric. Dev. 8, 23821205211000352 (2021). 33796793 10.1177/23821205211000352 PMC7975489

[39] G. Geller, P. A. Watkins, Addressing medical students’ negative bias toward patients with obesity through ethics education. AMA J. Ethics 20, E948–E959 (2018). 30346923 10.1001/amajethics.2018.948

[40] A. C. Gill, Y. Zhou, J. T. Greely, A. D. Beasley, J. Purkiss, M. Juneja, Longitudinal outcomes one year following implicit bias training in medical students. Med. Teach. 44, 744–751 (2022). 35021935 10.1080/0142159X.2021.2023120

[41] C. M. Gonzalez, M. Y. Kim, P. R. Marantz, Implicit bias and its relation to health disparities: A teaching program and survey of medical students. Teach. Learn. Med. 26, 64–71 (2014). 24405348 10.1080/10401334.2013.857341

[42] M. Jindal, R. L. J. Thornton, A. McRae, N. Unaka, T. J. Johnson, K. B. Mistry, Effects of a curriculum addressing racism on pediatric residents’ racial biases and empathy. J. Grad. Med. Educ. 14, 407–413 (2022). 35991090 10.4300/JGME-D-21-01048.1 PMC9380619

[43] J. W. Kanter, D. C. Rosen, K. E. Manbeck, H. M. L. Branstetter, A. M. Kuczynski, M. D. Corey, D. W. M. Maitland, M. T. Williams, Addressing microaggressions in racially charged patient-provider interactions: A pilot randomized trial. BMC Med. Educ. 20, 88 (2020). 32209082 10.1186/s12909-020-02004-9 PMC7092438

[44] B. Khandalavala, J. Koran-Scholl, J. Geske, Comprehensive obesity education for family medicine residents. Primer 4, 25 (2020). 33111052 10.22454/PRiMER.2020.525629 PMC7581191

[45] K. Knox, D. Simpson, J. Bidwell, W. Lehmann, Implementing an interprofessional anti-racism training with community partners during a pandemic: Outcomes and recommended strategies. WMJ. 120, S70–S73 (2021). 33819408

[46] M. Kokas, J. W. Fakhoury, M. Hoffert, S. Whitehouse, M. Van Harn, K. Baker-Genaw, Health care disparities: A practical approach to teach residents about self-bias and patient communication. J. Racial Ethn. Health Disparities 6, 1030–1034 (2019). 31215015 10.1007/s40615-019-00604-w

[47] J. R. Korndorffer, S. M. Wren, C. M. Pugh, M. T. Hawn, From listening to action. Ann. Surg. 274, 921–924 (2021). 33856378 10.1097/SLA.0000000000004891

[48] C. Lelutiu-Weinberger, K. A. Clark, J. E. Pachankis, Mental health provider training to improve LGBTQ competence and reduce implicit and explicit bias: A randomized controlled trial of online and in-person delivery. Psychol. Sex. Orientat. Gend. Divers. 10, 589–599 (2023). 38239562 10.1037/sgd0000560 PMC10794005

[49] K. F. Leslie, S. Sawning, M. A. Shaw, L. J. Martin, R. C. Simpson, J. E. Stephens, V. F. Jones, Changes in medical student implicit attitudes following a health equity curricular intervention. Med. Teach. 40, 372–378 (2018). 29171321 10.1080/0142159X.2017.1403014

[50] F. F. Liu, J. Coifman, E. McRee, J. Stone, A. Law, L. Gaias, R. Reyes, C. K. Lai, I. V. Blair, C. Yu, H. Cook, A. R. Lyon, A brief online implicit bias intervention for school mental health clinicians. Int. J. Environ. Res. Public Health 19, 679 (2022). 35055506 10.3390/ijerph19020679 PMC8776032

[51] W. H. Lu, P. Baldelli, P. Migdal, R. Iuli, L. Strano-Paul, K. L. Zacharoff, Early refill of an opioid medication: recognizing personal biases through clinical vignettes and OSCEs. MedEdPORTAL 18, 11234 (2022). 35497675 10.15766/mep_2374-8265.11234 PMC8986891

[52] B. Marr, S. H. Mickey, S. G. Blythe, J. Baruch, The weight of pain: What does a 10 on the pain scale mean? An innovative use of art in medical education to enhance pain management. J. Pain Symptom Manage. 57, 1182–1187 (2019). 30905676 10.1016/j.jpainsymman.2019.03.016

[53] J. J. Mayfield, E. M. Ball, K. A. Tillery, C. Crandall, J. Dexter, J. M. Winer, Z. M. Bosshardt, J. H. Welch, E. Dolan, E. R. Fancovic, A. I. Nanez, H. De May, E. Finlay, S. M. Lee, C. G. Streed, K. Ashraf, Beyond men, women, or both: A comprehensive, LGBTQ-inclusive, implicit-bias-aware, standardized-patient-based sexual history taking curriculum. MedEdPORTAL 13, 10634 (2017). 30800835 10.15766/mep_2374-8265.10634 PMC6338175

[54] A. Mendizabal, J. H. Fan, R. S. Price, R. H. Hamilton, Feasibility and effectiveness appraisal of a neurology residency health equities curriculum. J. Neurol. Sci. 431, 120040 (2021). 34748973 10.1016/j.jns.2021.120040

[55] S. C. Nelson, S. Prasad, H. W. Hackman, Training providers on issues of race and racism improve health care equity. Pediatr. Blood Cancer 62, 915–917 (2015). 25683782 10.1002/pbc.25448

[56] S. Nestorowicz, N. Saks, Addressing bias toward overweight patients: A training program for first-year medical students. Med. Sci. Educ. 31, 1115–1123 (2021). 34457955 10.1007/s40670-021-01282-2 PMC8368903

[57] D. Ogunyemi, Defeating unconscious bias: The role of a structured, reflective, and interactive workshop. J. Grad. Med. Educ. 13, 189–194 (2021). 33897951 10.4300/JGME-D-20-00722.1 PMC8054602

[58] P. Poitevien, C. Osman, Tackling implicit and explicit bias through objective structured teaching exercises for faculty. J. Grad. Med. Educ. 10, 353–354 (2018). 29946404 10.4300/JGME-D-17-00906.1 PMC6008013

[59] C. Reddyhough, V. Locke, G. Paulik, Changing healthcare professionals’ attitudes towards voice hearers: An education intervention. Community Ment. Health J. 57, 960–964 (2021). 32783075 10.1007/s10597-020-00695-4

[60] M. Ruben, N. S. Saks, Addressing implicit bias in first-year medical students: A longitudinal, multidisciplinary training program. Med. Sci. Educ. 30, 1419–1426 (2020). 34457809 10.1007/s40670-020-01047-3 PMC8368581

[61] J. N. Siegelman, C. Woods, B. Salhi, S. Heron, Health care disparities education using the implicit association test. Med. Educ. 50, 1158–1159 (2016). 27762020 10.1111/medu.13174

[62] J. Stone, G. B. Moskowitz, C. A. Zestcott, K. J. Wolsiefer, Testing active learning workshops for reducing implicit stereotyping of hispanics by majority and minority group medical students. Stigma Health 5, 94–103 (2020). 33134507 10.1037/sah0000179 PMC7597671

[63] J. A. Swift, V. Tischler, S. Markham, I. Gunning, C. Glazebrook, C. Beer, R. Puhl, Are anti-stigma films a useful strategy for reducing weight bias among trainee healthcare professionals? Results of a pilot randomized control trial. Obes. Facts 6, 91–102 (2013). 23466551 10.1159/000348714 PMC5644731

[64] K. Terry, N. A. Nickman, S. Mullin, P. Ghule, L. S. Tyler, Implementation of implicit bias awareness and action training in a pharmacy residency program. Am. J. Health. Syst. Pharm. 79, 1929–1937 (2022). 35880865 10.1093/ajhp/zxac199

[65] C. Traba, A. Jain, K. Pianucci, J. Rosen-Valverde, S. Chen, Down to the last dollar: Utilizing a virtual budgeting exercise to recognize implicit bias. MedEdPORTAL. 17, 11199 (2021). 34917754 10.15766/mep_2374-8265.11199 PMC8645532

[66] E. Ufomata, K. L. Eckstrand, P. Hasley, K. Jeong, D. Rubio, C. Spagnoletti, Comprehensive internal medicine residency curriculum on primary care of patients who identify as LGBT. LGBT Health 5, 375–380 (2018). 30141734 10.1089/lgbt.2017.0173

[67] E. Vaimberg, L. Demers, E. Ford, M. Sabatello, B. Stevens, S. Dasgupta, Project inclusive genetics: Exploring the impact of patient-centered counseling training on physical disability bias in the prenatal setting. PLOS ONE 16, e0255722 (2021). 34352009 10.1371/journal.pone.0255722 PMC8341652

[68] N. N. Wijayatunga, D. Bailey, S. S. Klobodu, J. A. Dawson, K. Knight, E. J. Dhurandhar, A short, attribution theory-based video intervention does not reduce weight bias in a nationally representative sample of registered dietitians: A randomized trial. Int. J. Obes. (Lond) 45, 787–794 (2021). 33504932 10.1038/s41366-021-00740-6

[69] D. Wu, L. Saint-Hilaire, A. Pineda, D. Hessler, G. W. Saba, R. Salazar, N. Olayiwola, The efficacy of an antioppression curriculum for health professionals. Fam. Med. 51, 22–30 (2019). 30412265 10.22454/FamMed.2018.227415

[70] A. J. Zeidan, U. G. Khatri, J. Aysola, F. S. Shofer, M. Mamtani, K. R. Scott, L. W. Conlon, B. L. Lopez, Implicit bias education and emergency medicine training: Step one? Awareness. AEM Educ. Train. 3, 81–85 (2019). 30680351 10.1002/aet2.10124 PMC6339553

[71] M. E. K. Amin, Addressing cultural competence and bias in treating migrant workers in pharmacies: Pharmacy students learning and changing norms. Res. Soc. Adm. Pharm. 18, 3362–3368 (2022). 10.1016/j.sapharm.2021.11.012 34857481

[72] M. E. Archambault, J. A. Van Rhee, G. S. Marion, S. J. Crandall, Utilizing implicit association testing to promote awareness of biases regarding age and disability. J. Physician Assist. Educ. 19, 20–26 (2008).

[73] A. N. Chary, M. F. Molina, F. Z. Dadabhoy, E. C. Manchanda, Addressing racism in medicine through a resident-led health equity retreat. West. J. Emerg. Med. 22, 41–44 (2020). 33439802 10.5811/westjem.2020.10.48697 PMC7806337

[74] M. H. Chin, M. M. Aburmishan, M. Zhu, Standup comedy principles and the personal monologue to explore interpersonal bias: Experiential learning in a health disparities course. BMC Med. Educ. 22, 80 (2022). 35123451 10.1186/s12909-022-03139-7 PMC8817666

[75] L. Clementz, M. McNamara, N. M. Burt, M. Sparks, M. K. Singh, Starting with Lucy: Focusing on human similarities rather than differences to address health care disparities. Acad. Med. 92, 1259–1263 (2017). 28272112 10.1097/ACM.0000000000001631

[76] M. DallaPiazza, M. S. Ayyala, M. L. Soto-Greene, Empowering future physicians to advocate for health equity: A blueprint for a longitudinal thread in undergraduate medical education. Med. Teach. 42, 806–812 (2020). 32180494 10.1080/0142159X.2020.1737322

[77] E. Díaz, T. Armah, C. T. Linse, A. Fiskin, A. Jordan, J. Hafler, Novel brief cultural psychiatry training for residents. Acad. Psychiatry 40, 366–368 (2016). 25636254 10.1007/s40596-015-0279-z

[78] J. Ellison, C. Gunther, M. B. Campbell, R. English, C. Lazarus, Critical consciousness as a framework for health equity-focused peer learning. MedEdPORTAL 17, 11145 (2021). 33937521 10.15766/mep_2374-8265.11145 PMC8079426

[79] H. F. Fitterman-Harris, J. S. Vander Wal, Weight bias reduction among first-year medical students: A quasi-randomized, controlled trial. Clin. Obes. 11, e12479 (2021). 34263533 10.1111/cob.12479

[80] E. Gatewood, C. Broholm, J. Herman, C. Yingling, Making the invisible visible: Implementing an implicit bias activity in nursing education. J. Prof. Nurs. 35, 447–451 (2019). 31857054 10.1016/j.profnurs.2019.03.004

[81] C. M. Gonzalez, A. D. Fox, P. R. Marantz, The evolution of an elective in health disparities and advocacy: Description of instructional strategies and program evaluation. Acad. Med. 90, 1636–1640 (2015). 26222321 10.1097/ACM.0000000000000850 PMC6949531

[82] A. K. Hughes, C. Luz, D. Hall, P. Gardner, C. W. Hennessey, L. Lammers, Transformative theatre: A promising educational tool for improving health encounters with LGBT older adults. Gerontol. Geriatr. Educ. 37, 292–306 (2016). 26886812 10.1080/02701960.2015.1127812

[83] V. Kerrigan, N. Lewis, A. Cass, M. Hefler, A. P. Ralph, “How can I do more?” Cultural awareness training for hospital-based healthcare providers working with high aboriginal caseload. BMC Med. Educ. 20, 173 (2020). 32471490 10.1186/s12909-020-02086-5 PMC7260793

[84] K. Matharu, J. F. Shapiro, R. R. Hammer, R. L. Kravitz, M. D. Wilson, F. T. Fitzgerald, Reducing obesity prejudice in medical education. Educ. Health 27, 231–237 (2014). 10.4103/1357-6283.152176 25758385

[85] P. A. McElfish, C. R. Long, B. Rowland, S. Moore, R. Wilmoth, B. Ayers, Improving culturally appropriate care using a community-based participatory research approach: Evaluation of a multicomponent cultural competency training program, Arkansas, 2015-2016. Prev. Chronic Dis. 14, E62 (2017). 28771402 10.5888/pcd14.170014 PMC5542547

[86] M. Medlock, A. Weissman, S. S. Wong, A. Carlo, M. Zeng, C. Borba, M. Curry, D. Shtasel, Racism as a unique social determinant of mental health: Development of a didactic curriculum for psychiatry residents. MedEdPORTAL 13, 10618 (2017). 29387786 10.15766/mep_2374-8265.10618 PMC5788030

[87] T. A. Mullett, S. N. Rooholamini, C. Gilliam, H. McPhillips, H. M. Grow, Description of a novel curriculum on equity, diversity and inclusion for pediatric residents. J. Natl. Med. Assoc. 113, 616–625 (2022). 34172296 10.1016/j.jnma.2021.05.014

[88] J. Perdomo, D. Tolliver, H. Hsu, Y. He, K. A. Nash, S. Donatelli, C. Mateo, C. Akagbosu, F. Alizadeh, A. Power-Hays, T. Rainier, D. J. Zheng, C. J. Kistin, R. J. Vinci, C. D. Michelson, Health equity rounds: An interdisciplinary case conference to address implicit bias and structural racism for faculty and trainees. MedEdPORTAL 15, 10858 (2019). 32166114 10.15766/mep_2374-8265.10858 PMC7050660

[89] J. Raney, R. Pal, T. Lee, S. R. Saenz, D. Bhushan, P. Leahy, C. Johnson, C. Kapphahn, M. A. Gisondi, K. Hoang, Words matter: An antibias workshop for health care professionals to reduce stigmatizing language. MedEdPORTAL 17, 11115 (2021). 33768147 10.15766/mep_2374-8265.11115 PMC7970642

[90] N. Rodriguez, E. Kintzer, J. List, M. Lypson, J. H. Grochowalski, P. R. Marantz, C. M. Gonzalez, Implicit bias recognition and management: Tailored instruction for faculty. J. Natl. Med. Assoc. 113, 566–575 (2021). 34140145 10.1016/j.jnma.2021.05.003 PMC8556183

[91] P. L. Schultz, J. Baker, Teaching strategies to increase nursing student acceptance and management of unconscious bias. J. Nurs. Educ. 56, 692–696 (2017). 29091241 10.3928/01484834-20171020-11

[92] R. Steed, Attitudes and beliefs of occupational therapists participating in a cultural competency workshop. Occup. Ther. Int. 17, 142–151 (2010). 20641132 10.1002/oti.299

[93] R. Steed, The effects of an instructional intervention on racial attitude formation in occupational therapy students. J. Transcult. Nurs. 25, 403–409 (2014). 24583876 10.1177/1043659614523471

[94] J. Sukhera, K. Miller, C. Scerbo, A. Milne, R. Lim, C. Watling, Implicit stigma recognition and management for health professionals. Acad. Psychiatry. 44, 59–63 (2020). 31701387 10.1007/s40596-019-01133-8

[95] G. S. Tajeu, L. Juarez, J. H. Williams, J. Halanych, I. Stepanikova, A. A. Agne, J. Stone, A. L. Cherrington, Development of a multicomponent intervention to decrease racial bias among healthcare staff. J. Gen. Intern. Med. 37, 1970–1979 (2022). 35266123 10.1007/s11606-022-07464-x PMC9198170

[96] S. Wasmuth, K. Pritchard, C. Milton, E. Smith, A mixed-method analysis of community-engaged theatre illuminates black women’s experiences of racism and addresses healthcare inequities by targeting provider bias. Inquiry 57, 46958020976255 (2020). 33300406 10.1177/0046958020976255 PMC7734486

[97] T. White-Davis, J. Edgoose, J. S. B. Speights, K. Fraser, J. M. Ring, J. Guh, G. W. Saba, Addressing racism in medical education: An interactive training module. Fam. Med. 50, 364–368 (2018). 29762795 10.22454/FamMed.2018.875510

[98] A. Zeidan, A. Tiballi, M. Woodward, I. M. Di Bartolo, Targeting implicit bias in medicine: Lessons from art and archaeology. West. J. Emerg. Med. 21, 1–3 (2020). 31913809 10.5811/westjem.2019.9.44041 PMC6948688

[99] N. B. Collier, L. Taylor, Fostering awareness of implicit bias using an adapted visual thinking strategy and reflection. J. Physician Assist. Educ. 33, 145–147 (2022). 35511460 10.1097/JPA.0000000000000425

[100] E. Geiser, L. V. Schilter, J. M. Carrier, C. Clair, J. Schwarz, Reflexivity as a tool for medical students to identify and address gender bias in clinical practice: A qualitative study. Patient Educ. Couns. 105, 3521–3528 (2022). 36075808 10.1016/j.pec.2022.08.017

[101] C. M. Gonzalez, S. Nava, J. List, A. Liguori, P. R. Marantz, How assumptions and preferences can affect patient care: An introduction to implicit bias for first-year medical students. MedEdPORTAL 17, 11162 (2021). 34263027 10.15766/mep_2374-8265.11162 PMC8236500

[102] A. L. Holm, M. Rowe Gorosh, M. Brady, D. White-Perkins, Recognizing privilege and bias: An interactive exercise to expand health care providers’ personal awareness. Acad. Med. 92, 360–364 (2017). 27355785 10.1097/ACM.0000000000001290

[103] R. Khazanchi, H. Keeler, S. Strong, E. R. Lyden, P. Davis, B. K. Grant, J. R. Marcelin, Building structural competency through community engagement. Clin. Teach. 18, 535–541 (2021). 34278725 10.1111/tct.13399

[104] B. McMichael, A. Nickel, E. A. Duffy, L. Skjefte, L. Lee, P. Park, S. C. Nelson, S. Puumala, A. P. Kharbanda, The impact of health equity coaching on patient’s perceptions of cultural competency and communication in a pediatric emergency department: An intervention design. J. Patient Exp. 6, 257–264 (2019). 31853480 10.1177/2374373518798111 PMC6908992

[105] M. D. Sherman, J. Ricco, S. C. Nelson, S. J. Nezhad, S. Prasad, Implicit bias training in a residency program: Aiming for enduring effects. Fam. Med. 51, 677–681 (2019). 31509218 10.22454/FamMed.2019.947255

[106] O. Solá, C. Marquez, Integrating social determinants of health into clinical training during the COVID-19 pandemic. Primer 4, 28 (2020). 33111055 10.22454/PRiMER.2020.449390 PMC7581196

[107] C. R. Teal, R. E. Shada, A. C. Gill, B. M. Thompson, E. Frugé, G. B. Villarreal, P. Haidet, When best intentions aren’t enough: Helping medical students develop strategies for managing bias about patients. J. Gen. Intern. Med. 25 ( Suppl. 2), S115–S118 (2010). 20352504 10.1007/s11606-009-1243-y PMC2847119

[108] T. J. West, K. Loomer, T. R. Wyatt, How diverse is your universe? An activity for students to reflect on ethnoracial diversity during orientation. MedEdPORTAL 15, 10840 (2019). 31890871 10.15766/mep_2374-8265.10840 PMC6897539

[109] A. A. White, H. J. Logghe, D. A. Goodenough, L. L. Barnes, A. Hallward, I. M. Allen, D. W. Green, E. Krupat, R. Llerena-Quinn, Self-awareness and cultural identity as an effort to reduce bias in medicine. J. Racial Ethn. Health Disparities 5, 34–49 (2018). 28342029 10.1007/s40615-017-0340-6

