# Supplementary material for: The nature and validity of implicit bias training for health care providers and trainees: A systematic review
Source: Sci Adv. 2024 Aug 14;10(33):eado5957. doi: 10.1126/sciadv.ado5957 (PMC11323883; doi:10.1126/sciadv.ado5957)
Supplement: Supplementary file 1 — Tables S1, S3 to S5 Legend for table S2 Legend for data S1 References [file sciadv.ado5957_sm.pdf]

Science Advances
AAAS

Supplementary Materials for

The nature and validity of implicit bias training for health care providers and trainees: A systematic review

Nao Hagiwara *et al.*

Corresponding author: Nao Hagiwara, nh6ty@virginia.edu

Sci. Adv. **10**, eado5957 (2024)
DOI: 10.1126/sciadv.ado5957

The PDF file includes:

Tables S1, S3 to S5
Legend for table S2
Legend for data S1
References

Other Supplementary Material for this manuscript includes the following:

Table S2
Data S1

Table S1. Summary of the full search strategies.

Medline (Ovid) September 1, 2021		
1	exp Education, Professional/ or exp Health Personnel/ or exp "Internship and Residency"/	796979
2	("medical education" or clerkship or internship or residency or "medical student" or "medical students" or resident or trainee or fellow or healthcare or doctor or physician or provider or nurse or pharmac* or counsel* or therapist or clinician or dentist* or optometr* or pediatrician or "medical school" or "medical schools " or stem-h or stemm or attending or faculty).mp.	5413498
3	1 or 2	5817967
4	exp Stereotyping/ or exp Prejudice/	41492
5	("implicit association test" or microaggression or microaggressions or "sequential priming task" or "sequential priming tasks" or "racial attitude").mp.	1524
6	((implicit or unconscious or nonconscious or non-conscious or automatic or spontaneous) adj3 attitude).mp.	226
7	((implicit or unconscious or nonconscious or non-conscious or automatic or spontaneous) adj3 stereotyp*).mp.	299
8	((implicit or unconscious or nonconscious or non-conscious or automatic or spontaneous) adj3 prejudice*).mp.	132
9	((implicit or unconscious or nonconscious or non-conscious or automatic or spontaneous) adj3 bias).mp.	1535
10	4 or 5 or 6 or 7 or 8 or 9	43843
11	(curricul* or instruction or instructional or train* or interven* or workshop or educational).mp.	2018534
12	3 and 10 and 11	3195
13	limit 12 to dt=20030101-20210831	2618
Embase (Ovid) September 1, 2021		
1	exp medical education/	333587
2	exp paramedical personnel/	530856
3	exp health care personnel/	1696919
4	exp paramedical education/	92006
5	("medical education" or clerkship or internship or residency or "medical student" or "medical students" or resident or trainee or fellow or healthcare or doctor or physician or provider or nurse or pharmac* or counsel* or therapist or clinician or dentist* or optometr* or pediatrician or "medical school" or "medical schools " or stem-h or stemm or attending or faculty).mp.	5930114
6	1 or 2 or 3 or 4 or 5	6643532
7	exp stereotypy/	16385
8	exp prejudice/	3464
9	("implicit association test" or microaggression or microaggressions or "sequential priming task" or "sequential priming tasks" or "racial attitude").mp.	1736

10	((implicit or unconscious or nonconscious or non-conscious or automatic or spontaneous) adj3 attitude).mp.	276
11	((implicit or unconscious or nonconscious or non-conscious or automatic or spontaneous) adj3 stereotyp*).mp.	337
12	((implicit or unconscious or nonconscious or non-conscious or automatic or spontaneous) adj3 prejudice*).mp.	148
13	((implicit or unconscious or nonconscious or non-conscious or automatic or spontaneous) adj3 bias).mp.	1878
14	7 or 8 or 9 or 10 or 11 or 12 or 13	23075
15	(curricul* or instruction or instructional or train* or interven* or workshop or educational).mp.	2752293
16	6 and 14 and 15	1744
17	limit 16 to dc=20030101-20210831	1650
PsycINFO(Proquest) September 1, 2021		
1	(DE "Clinical Psychology Graduate Training" OR DE "Clinical Psychology Internship" OR DE "Graduate Education" OR DE "Dental Education" OR DE "Medical Education" OR DE "Medical Internship" OR DE "Medical Residency" OR DE "Psychiatric Training" OR DE "Rehabilitation Education" OR DE "Nursing Education" OR DE "Health Personnel") OR ("medical education" or clerkship or internship or residency or "medical student" or "medical students" or resident or trainee or fellow or healthcare or doctor or physician or provider or nurse or pharmac* or counsel* or therapist or clinician or dentist* or optometr* or pediatrician or "medical school" or "medical schools " or stem-h or stemm or attending or faculty)	1,307,199
2	DE "Prejudice" OR DE "Implicit Attitudes" OR DE "Implicit Bias" OR DE "Implicit Bias" OR DE "Race and Ethnic Discrimination" OR DE "Racial Disparities" OR DE "Racial and Ethnic Attitudes" OR DE "Antiracism" OR DE "AntiSemitism" OR DE "Ethnocentrism" OR DE "Racism" OR DE "Racial Bias" OR DE "Racism" OR DE "Stereotyped Attitudes"	47,201
3	(implicit bias or unconscious bias or nonconscious bias or non-conscious bias or automatic bias or spontaneous bias or prejudice or implicit prejudice or unconscious prejudice or nonconscious prejudice or non-conscious prejudice or automatic prejudice or spontaneous prejudice or stereotyp* or implicit stereotyp* or unconscious stereotyp* or nonconscious stereotyp* or non-conscious stereotyp* or automatic stereotyp* or spontaneous stereotyp* or racial attitude or implicit racial attitude or unconscious racial attitude or nonconscious racial attitude or non-conscious racial attitude or automatic racial attitude or spontaneous racial attitude or implicit attitude or unconscious attitude or nonconscious attitude or non-conscious attitude or automatic attitude or spontaneous attitude)	109,161
4	S2 OR S3	119,920
5	(curricul* or instruction or instructional or train* or interven* or workshop or educational)	1,372,547
6	(S1 OR S4 OR S5) AND (S1 AND S4 AND S5)	10,096
7	(("implicit association test" or microaggression or microaggressions or "sequential	20,881

	priming task" or "sequential priming tasks" or "racial attitude")) OR (((implicit or unconscious or nonconscious or non-conscious or automatic or spontaneous) N3 attitude)) OR (((implicit or unconscious or nonconscious or non-conscious or automatic or spontaneous) N3 stereotyp*)) OR (((implicit or unconscious or nonconscious or non-conscious or automatic or spontaneous) N3 prejudice*)) OR (((implicit or unconscious or nonconscious or non-conscious or automatic or spontaneous) N3 bias))	
8	S2 OR S7	63,712
9	S1 AND S5 AND S8	5,224
CINAHL(EBSCO) September 1, 2021		
1	MH "Education, Health Sciences+")	293,181
2	(MH "Health Personnel+")	606,349
3	(MH "Education, Clinical+")	18,638
4	S1 OR S2 OR S3	843,038
5	(S1 OR S2 OR S3) OR (("medical education" or clerkship or internship or residency or "medical student" or "medical students" or resident or trainee or fellow or healthcare or doctor or physician or provider or nurse or pharmac* or counsel* or therapist or clinician or dentist* or optometr* or pediatrician or "medical school" or "medical schools " or stem-h or stemm or attending or faculty))	2,321,695
6	MH "Prejudice+") OR (MH "Stereotyping")	26,550
7	(implicit bias or unconscious bias or nonconscious bias or non-conscious bias or automatic bias or spontaneous bias or prejudice or implicit prejudice or unconscious prejudice or nonconscious prejudice or non-conscious prejudice or automatic prejudice or spontaneous prejudice or stereotyp* or implicit stereotyp* or unconscious stereotyp* or nonconscious stereotyp* or non-conscious stereotyp* or automatic stereotyp* or spontaneous stereotyp* or racial attitude or implicit racial attitude or unconscious racial attitude or nonconscious racial attitude or non-conscious racial attitude or automatic racial attitude or spontaneous racial attitude or implicit attitude or unconscious attitude or nonconscious attitude or non-conscious attitude or automatic attitude or spontaneous attitude)	29,633
8	S6 OR S7	42,731
9	(curricul* or instruction or instructional or train* ori nterven* or workshop or educational)	919,122
10	S5 AND S8 AND S9	4,409
11	(("implicit association test" or microaggression or microaggressions or "sequential priming task" or "sequential priming tasks" or "racial attitude")) OR (((implicit or unconscious or nonconscious or non-conscious or automatic or spontaneous) N3 attitude)) OR (((implicit or unconscious or nonconscious or non-conscious or automatic or spontaneous) N3 stereotyp*)) OR (((implicit or unconscious or nonconscious or non-conscious or automatic or spontaneous) N3 prejudice*)) OR (((implicit or unconscious or nonconscious or non-conscious or automatic or spontaneous) N3 bias))	5,300
12	S6 OR S11	31,065
13	S10 OR S12	3,067

Social Work Abstracts(EBSCO) September 1, 2021		
S5	S2 AND S3 AND S4	103
S4	curricul* or instruction or instructional or train* or interven* or workshop or educational	19,676
S3	bias OR implicit bias or unconscious bias or nonconscious bias or non-conscious bias or automatic bias or spontaneous bias or prejudice or implicit prejudice or unconscious prejudice or nonconscious prejudice or non-conscious prejudice or automatic prejudice or spontaneous prejudice or stereotyp* or implicit stereotyp* or unconscious stereotyp* or nonconscious stereotyp* or non-conscious stereotyp* or automatic stereotyp* or spontaneous stereotyp* or racial attitude or implicit racial attitude o ...	1,797
S2	("medical education" or clerkship or internship or residency or "medical student" or "medical students" or resident or trainee or fellow or healthcare or doctor or physician or provider or nurse or pharmac* or counsel* or therapist or clinician or dentist* or optometr* or pediatrician or "medical school" or "medical schools " or stem-h or stemm or attending or faculty) OR (health professions education OR nursing education OR pharmacy education OR health personnel OR healthcare personnel)	19,537

Scopus(Elsevier) September 1, 2021		
1	curricul* OR instruction OR instructional OR train* OR interven* OR workshop OR educational	11,634,135
2	TITLE-ABS-KEY ("medical education" OR clerkship OR internship OR residency OR "medical student" OR "medical students" OR resident OR trainee OR fellow OR healthcare OR doctor OR physician OR provider OR nurse OR pharmac* OR counsel* OR therapist OR clinician OR dentist* OR optometr* OR pediatrician OR "medical school" OR "medical schools " OR stem-h OR stemm OR attending OR faculty)	4,835,776
3	TITLE-ABS-KEY (("Racial attitude") OR (((implicit OR unconscious OR nonconscious OR non-conscious OR automatic OR spontaneous) W/3 attitude)) OR (((implicit OR unconscious OR nonconscious OR non-conscious OR automatic OR spontaneous) W/3 stereotyp*)) OR (((implicit OR unconscious OR nonconscious OR non-conscious OR automatic OR spontaneous) W/3 prejudice*)) OR (((implicit OR unconscious OR nonconscious OR non-conscious OR automatic OR spontaneous) W/3 bias))) View Less	8,316
4	1 AND 2 AND 3	1,033
5	((TITLE-ABS-KEY ("medical education" OR clerkship OR internship OR residency OR "medical student" OR "medical students" OR resident OR trainee OR fellow OR healthcare OR doctor OR physician OR provider OR nurse OR pharmac* OR counsel* OR therapist OR clinician OR dentist* OR optometr* OR pediatrician OR "medical school" OR "medical schools " OR stem-h OR stemm OR attending OR faculty)) AND (curricul* OR instruction OR instructional OR train* OR interven* OR workshop OR educational)) AND (TITLE-ABS-KEY ("implicit association test" OR microaggression OR microaggressions OR "sequential priming task" OR "sequential priming tasks"))	450

6	4 OR 5	1483
MedEdPortal September 1, 2021		
bias OR prejudice OR stereotyp* OR discrimination OR microaggression		

Table S2. Additional studies that were excluded during full-text screening and corresponding references.

Table S3. Summary of MERSQI coding and scores for 38 quantitative studies.

Author (year)	Study design	Sampling		Data type	Validity			Data analysis		Outcome	Total score
		Institutions	Response rate		Content	Internal structure	Relationships to other variables	Sophistication	Appropriateness		
Bartlett (2015)[33]	1.5	0.5	1.0	3.0	1.0	0.0	0.0	0.0	1.0	1.0	9
Bernstein (2016)[34]	1.0	0.5	1.5	1.0	0.0	0.0	0.0	0.0	1.0	1.0	6
Chapman (2018)[35]	1.5	0.5	1.5	3.0	1.0	1.0	1.0	1.0	2.0	1.0	13.5
Corsino (2018)[36]	1.5	0.5	1.5	1.0	0.0	0.0	0.0	0.0	1.0	1.0	6.5
DallaPiazza (2018)[37]	1.0	0.5	1.0	1.0	0.0	0.0	0.0	1.0	1.0	1.0	6.5
Davis (2021)[38]	1.0	0.5	1.5	1.0	0.0	0.0	0.0	0.0	1.0	1.0	6
Geller (2018)[39]	1.0	0.5	1.0	1.0	0.0	0.0	0.0	0.0	1.0	1.0	5.5
Gill (2022)[40]	1.5	0.5	1.5	1.0	0.0	0.0	0.0	1.0	2.0	1.0	8.5
Gonzalez (2014)[41]	1.0	0.5	1.0	1.0	0.0	0.0	0.0	0.0	2.0	1.0	6.5

Jindal (2022)[42]	1.5	1.0	1.0	3.0	1.0	0.0	0.0	1.0	2.0	1.0	11.5
Kanter (2020)[43]	3.0	0.5	1.5	3.0	1.0	1.0	1.0	1.0	2.0	2.0	16
Khandalavala (2020)[44]	1.0	0.5	1.0	1.0	0.0	0.0	0.0	0.0	2.0	1.0	6.5
Knox (2021)[45]	1.0	0.5	1.0	1.0	0.0	0.0	0.0	0.0	1.0	1.0	5.5
Kokas (2019)[46]	1.5	0.5	1.0	1.0	1.0	1.0	0.0	1.0	2.0	1.0	10
Korndorffer (2021)[47]	1.0	0.5	0.5	1.0	0.0	0.0	0.0	1.0	1.0	1.0	6
Lelutiu-Weinberger (2022)[48]	3.0	1.5	1.0	3.0	1.0	0.0	0.0	1.0	2.0	1.0	13.5
Leslie (2018)[49]	3.0	0.5	Not appliable	3.0	1.0	1.0	1.0	1.0	2.0	1.0	13.5
Liu (2022)[50]	1.5	1.5	1.5	3.0	1.0	0.0	0.0	1.0	2.0	1.5	13
Lu (2022)[51]	1.0	0.5	1.5	3.0	0.0	0.0	0.0	1.0	2.0	2.0	11
Marr (2019)[52]	1.0	0.5	1.5	1.0	0.0	0.0	0.0	1.0	1.0	1.0	7

Mayfield (2017)[53]	1.5	0.5	0.5	1.0	0.0	0.0	0.0	1.0	2.0	1.0	7.5
Mendizabal (2021)[54]	1.5	0.5	1.0	1.0	0.0	0.0	0.0	0.0	1.0	1.0	6
Nelson (2015)[55]	1.5	0.5	1.5	1.0	0.0	0.0	0.0	1.0	2.0	1.0	8.5
Nestorowicz (2021)[56]	2.0	0.5	Not appliable	3.0	1.0	1.0	0.0	1.0	2.0	1.0	11.5
Ogunyemi (2021)[57]	1.5	1.5	1.0	1.0	0.0	0.0	0.0	0.0	2.0	1.0	8
Poitevien (2018)[58]	1.0	0.5	1.5	1.0	0.0	0.0	0.0	0.0	2.0	1.0	7
Reddyhough (2021)[59]	1.5	0.5	1.0	3.0	1.0	0.0	0.0	1.0	2.0	1.0	11
Ruben (2020)[60]	2.0	0.5	1.5	3.0	1.0	0.0	0.0	1.0	2.0	1.0	12
Siegelman (2016)[61]	1.0	0.5	1.0	1.0	0.0	0.0	0.0	1.0	1.0	1.0	6.5
Stone (2020)[62]	1.5	0.5	1.0	3.0	1.0	0.0	1.0	1.0	2.0	1.0	12
Swift (2013)[63]	3.0	0.5	0.5	3.0	1.0	0.0	0.0	1.0	2.0	1.0	12

Terry (2022)[64]	1.5	0.5	0.5	3.0	0.0	0.0	0.0	0.0	1.0	1.5	8
Traba (2021)[65]	1.0	0.5	1.5	1.0	0.0	0.0	0.0	1.0	1.0	1.0	7
Ufomata (2018)[66]	1.5	0.5	1.0	3.0	1.0	0.0	0.0	1.0	2.0	1.5	11.5
Vaimberg (2021)[67]	2.0	1.5	0.5	3.0	1.0	0.0	1.0	1.0	2.0	1.0	13
Wijayatunga (2021)[68]	3.0	1.5	0.5	3.0	1.0	0.0	1.0	1.0	2.0	1.0	14
Wu (2019)[69]	1.5	0.5	1.5	1.0	0.0	0.0	0.0	1.0	2.0	1.0	8.5
Zeidan (2019)[70]	1.5	0.5	1.0	1.0	0.0	0.0	0.0	0.0	1.0	1.0	6

Table S4. Summary of MERSQI and CASP coding and scores for 28 mixed methods studies.

Author (year)	Quantitative Part										
	Study design	Sampling		Data type	Validity			Data analysis		Outcome	Total score
		Institutions	Response rate		Content	Internal structure	Relationships to other variables	Sophistication	Appropriateness		
	Qualitative Part										
	Clear aims	Method	Design	Recruit-ment	Data collection	Relation-ship	Ethical issues	Analysis	Clear findings	Value	% "yes"
Amin (2022)[71]	1.0	0.5	1.5	1.0	1.0	0.0	0.0	1.0	1.0	1.0	8
	Yes	Yes	Yes	Yes	Yes	No	Yes	No	Yes	Can't tell	70
Archambault (2008)[72]	1.5	0.5	1.0	3.0	1.0	1.0	0.0	0.0	1.0	1.0	10
	Yes	Yes	No	Yes	No	No	Yes	No	No	Yes	50
Chary (2020)[73]	1.0	0.5	1.0	1.0	0.0	0.0	0.0	0.0	1.0	1.0	5.5
	No	Can't tell	Can't tell	Can't tell	No	No	No	No	No	Yes	10
Chin (2022)[74]	1.0	0.5	0.5	1.0	0.0	0.0	0.0	1.0	1.0	1.0	6
	Yes	Can't tell	Can't tell	Yes	Can't tell	Yes	Yes	Can't tell	Yes	Can't tell	50
Clementz (2017)[75]	1.0	0.5	1.5	1.0	0.0	0.0	0.0	1.0	1.0	1.0	7
	Can't tell	Can't tell	Can't tell	Yes	No	No	Yes	No	No	Yes	30

Dalla Piazza (2020)[76]	1.0	0.5	1.0	1.0	0.0	0.0	0.0	0.0	1.0	1.0	5.5
	Can't tell	Can't tell	Can't tell	Yes	No	No	No	No	No	Yes	20
Diaz (2016)[77]	1.5	0.5	1.0	1.0	0.0	0.0	0.0	0.0	2.0	1.0	7
	No	Can't tell	Can't tell	Yes	No	No	No	No	No	Yes	20
Ellison (2021)[78]	1.5	0.5	1.5	1.0	0.0	0.0	0.0	1.0	2.0	1.0	8.5
	No	Can't tell	Can't tell	No	No	No	No	No	No	Yes	10
Fitterman-Harris (2021)[79]	3.0	0.5	1.0	3.0	1.0	1.0	1.0	1.0	2.0	1.0	14.5
	Yes	Yes	Yes	Yes	Yes	No	Yes	Yes	Yes	Yes	90
Gatewood (2019)[80]	1.0	1.5	1.5	1.0	0.0	0.0	0.0	0.0	1.0	1.0	7
	No	Can't tell	Can't tell	No	No	No	No	No	No	No	0
Gonzalez (2015)[81]	1.5	0.5	1.5	3.0	0.0	0.0	0.0	0.0	2.0	1.5	10
	Yes	Yes	Yes	Yes	Yes	No	Yes	Yes	Yes	Yes	90
Hughes (2016)[82]	1.5	1.5	1.5	1.0	0.0	0.0	0.0	0.0	1.0	1.0	7.5
	No	Can't tell	Can't tell	Yes	No	No	No	No	Yes	No	20
Kerrigan (2020)[83]	1.0	0.5	1.5	1.0	0.0	0.0	0.0	1.0	1.0	1.0	7
	Yes	Yes	Can't tell	Yes	Yes	No	Yes	Yes	Yes	Yes	80

Matharu (2014)[84]	3.0	1.5	1.5	3.0	1.0	1.0	0.0	1.0	2.0	1.0	15
	Yes	Yes	Yes	Yes	Yes	Can't tell	Yes	Can't tell	Yes	Yes	80
McElfish (2017)[85]	1.0	1.5	1.0	1.0	0.0	0.0	0.0	0.0	1.0	1.0	6.5
	Yes	Yes	Can't tell	No	Yes	No	No	No	No	Yes	40
Medlock (2017)[86]	1.0	0.5	1.0	1.0	0.0	0.0	0.0	0.0	1.0	1.0	5.5
	No	Can't tell	Can't tell	Can't tell	Can't tell	No	No	No	No	No	0
Mullett (2021)[87]	1.0	0.5	1.5	1.0	0.0	0.0	0.0	1.0	1.0	1.0	7
	No	Can't tell	Can't tell	Yes	Can't tell	No	Can't tell	Yes	Yes	Yes	40
Perdomo (2019)[88]	1.0	0.5	1.0	1.0	0.0	0.0	0.0	1.0	1.0	1.0	6.5
	No	Can't tell	Can't tell	Yes	Can't tell	No	Yes	No	Yes	Yes	40
Raney (2021)[89]	1.0	1.5	1.5	1.0	0.0	0.0	0.0	1.0	1.0	1.0	8
	No	Can't tell	Can't tell	Can't tell	Can't tell	No	Yes	No	Yes	Yes	30
Rodriguez (2021)[90]	1.5	1.5	1.0	1.0	1.0	1.0	0.0	1.0	2.0	1.0	11
	Yes	Yes	Yes	Yes	Can't tell	No	Yes	Yes	Yes	Yes	80
Schultz (2017)[91]	1.5	0.5	0.5	1.0	0.0	0.0	0.0	0.0	1.0	1.0	5.5
	No	Can't tell	Can't tell	Yes	Can't tell	No	Yes	Can't tell	No	Can't tell	20

Steed (2010)[92]	1.5	1.5	1.5	3.0	1.0	0.0	0.0	1.0	2.0	1.0	12.5
	Yes	Yes	Yes	Yes	Yes	No	Yes	Yes	Yes	Yes	90
Steed (2014)[93]	1.5	0.5	1.5	1.0	1.0	0.0	0.0	1.0	2.0	1.0	9.5
	No	Can't tell	Can't tell	Yes	Can't tell	Yes	Yes	Yes	Yes	Yes	60
Sukhera (2020)[94]	1.5	0.5	1.5	1.0	1.0	0.0	0.0	1.0	2.0	1.0	9.5
	Yes	Yes	Yes	Yes	Yes	No	Can't tell	No	Yes	Yes	70
Tajeu (2022)[95]	1.5	1.5	0.5	3.0	1.0	0.0	0.0	1.0	2.0	1.5	12
	No	No	Can't tell	Yes	Can't tell	No	Yes	Yes	Yes	Yes	50
Wasmuth (2020)[96]	1.0	0.5	1.5	1.0	1.0	1.0	0.0	1.0	2.0	1.0	10
	No	Can't tell	Can't tell	Yes	Can't tell	Can't tell	Yes	Can't tell	Yes	Yes	40
White-Davis (2018)[97]	1.5	1.5	1.0	1.0	0.0	0.0	0.0	1.0	2.0	1.0	9
	Yes	Yes	Can't tell	Yes	Can't tell	Yes	Yes	No	Yes	Can't tell	60
Zeidan (2020)[98]	1.5	0.5	1.5	1.0	0.0	0.0	0.0	1.0	2.0	1.0	8.5
	Yes	Yes	Yes	Yes	Can't tell	No	No	Yes	Yes	Can't tell	60

Table S5. Summary of CASP coding and scores for 11 qualitative studies.

Author (year)	Clear aims	Method	Design	Recruitment	Data collection	Relationship	Ethical issues	Analysis	Clear findings	Value	% "yes"
Collier (2022)[99]	No	Can't tell	Can't tell	Can't tell	Can't tell	No	Can't tell	Can't tell	No	No	0
Geiser (2022)[100]	Yes	Yes	Yes	Yes	Yes	No	Yes	Yes	Yes	Yes	90
Gonzalez (2021)[101]	Can't tell	Can't tell	Can't tell	Yes	Can't tell	No	Yes	Can't tell	Yes	No	30
Holm (2017)[102]	Yes	Yes	Can't tell	No	No	No	Yes	No	No	Yes	40
Khazanchi (2021)[103]	Can't tell	Can't tell	Can't tell	Yes	Can't tell	Yes	Yes	Yes	Yes	Yes	60
McMichael (2019)[104]	Yes	Yes	No	No	No	No	Yes	Can't tell	Yes	Yes	50
Sherman (2019)[105]	Yes	Yes	Yes	Yes	Yes	No	Yes	Can't tell	Yes	Can't tell	70
Sola (2020)[106]	No	Can't tell	Can't tell	Can't tell	Can't tell	No	Yes	Can't tell	No	No	10
Teal (2010)[107]	Yes	Yes	Yes	Yes	Yes	No	Yes	Yes	Yes	Yes	90
West (2019)[108]	Yes	Yes	Can't tell	Yes	Can't tell	No	No	Can't tell	Yes	Can't tell	40
White (2018)[109]	No	No	No	Can't tell	Can't tell	No	Yes	No	No	Can't tell	10

Data S1. Characteristics of individual implicit bias training reported in the 77 studies.

REFERENCES AND NOTES

1. Institute of Medicine (US) Committee on Understanding and Eliminating Racial and Ethnic Disparities in Health Care, *Unequal Treatment: Confronting Racial and Ethnic Disparities in Health Care* (National Academies Press, 2003); www.ncbi.nlm.nih.gov/books/NBK220358/

2. I. W. Maina, T. D. Belton, S. Ginzberg, A. Singh, T. J. Johnson, A decade of studying implicit racial/ethnic bias in healthcare providers using the implicit association test. *Soc. Sci. Med.* **199**, 219–229 (2018).

3. C. FitzGerald, S. Hurst, Implicit bias in healthcare professionals: A systematic review. *BMC Med. Ethics* **18**, 19–18 (2017).

4. S. Heath, AMA joins industry efforts against medical racism, implicit bias (Patient Engagement Hit, 2023); https://patientengagementhit.com/news/ama-joins-industry-efforts-against-medical-racism-implicit-bias.

5. L. A. Cooper, S. Saha, M. van Ryn, Mandated implicit bias training for health professionals—A step toward equity in health care. *JAMA Health Forum* **3**, e223250 (2022).

6. U.S. Senator Cory Booker of New Jersey, Booker, Underwood, Adams reintroduce the bicameral Momnibus Act to end America’s maternal health crisis (2023); www.booker.senate.gov/news/press/booker-underwood-adams-reintroduce-the-bicameral-momnibus-act-to-end-americas-maternal-health-crisis.

7. 118th Congress (2023-2024), *H.R.3305 - Black Maternal Health Momnibus Act* (2023); www.congress.gov/bill/118th-congress/house-bill/3305/text.

8. P. Papinemi, S. Filson, T. Harrison, M. McIntosh, *Adopting an anti-racist medical curriculum* (The BMJ, 2021); https://blogs.bmj.com/bmj/2021/02/19/adopting-an-anti-racist-medical-curriculum/.

9. N. Hagiwara, L. A. Penner, R. Gonzalez, S. Eggly, J. F. Dovidio, S. L. Gaertner, T. West, T.

L. Albrecht, Racial attitudes, physician-patient talk time ratio, and adherence in racially discordant medical interactions. *Soc. Sci. Med.* **87**, 123–131 (2013).

10. N. Hagiwara, R. B. Slatcher, S. Eggly, L. A. Penner, Physician racial bias and word use during racially discordant medical interactions. *Health Commun.* **32**, 401–408 (2017).

11. L. A. Penner, J. F. Dovidio, R. Gonzalez, T. L. Albrecht, R. Chapman, T. Foster, F. W. K. Harper, N. Hagiwara, L. M. Hamel, A. F. Shields, S. Gadgeel, M. S. Simon, J. G. Griggs, S. Eggly, The effects of oncologist implicit racial bias in racially discordant oncology interactions. *J. Clin. Oncol.* **34**, 2874–2880 (2016).

12. L. A. Cooper, D. L. Roter, K. A. Carson, M. C. Beach, J. A. Sabin, A. G. Greenwald, T. S. Inui, The associations of clinicians' implicit attitudes about race with medical visit communication and patient ratings of interpersonal care. *Am. J. Public Health* **102**, 979–987 (2012).

13. E. Dehon, N. Weiss, J. Jones, W. Faulconer, E. Hinton, S. Sterling, A systematic review of the impact of physician implicit racial bias on clinical decision making. *Acad. Emerg. Med.* **24**, 895–904 (2017).

14. N. Hagiwara, J. F. Dovidio, J. Stone, L. A. Penner, Applied racial/ethnic healthcare disparities research using implicit measures. *Soc. Cogn.* **38**, s68–s97 (2020).

15. N. Hagiwara, F. W. Kron, M. W. Scerbo, G. S. Watson, A call for grounding implicit bias training in clinical and translational frameworks. *Lancet* **395**, 1457–1460 (2020).

16. S. H. Woolf, The meaning of translational research and why it matters. *JAMA* **299**, 211–213 (2008).

17. C. A. Zestcott, I. V. Blair, J. Stone, Examining the presence, consequences, and reduction of implicit bias in health care: A narrative review. *Group Process. Intergroup Relat.* **19**, 528–542 (2016).

18. B. Ahadinezhad, O. Khosravizadeh, A. Maleki, A. Hashtroodi, Implicit racial bias among

medical graduates and students by an IAT measure: A systematic review and meta-analysis. *Ir. J. Med. Sci.* **191**, 1941–1949 (2022).

19. W. J. Hall, M. V. Chapman, K. M. Lee, Y. M. Merino, T. W. Thomas, B. K. Payne, E. Eng, S. H. Day, T. Coyne-Beasley, Implicit racial/ethnic bias among health care professionals and its influence on health care outcomes: A systematic review. *Am. J. Public Health* **105**, e60–e76 (2015).

20. C. FitzGerald, A. Martin, D. Berner, S. Hurst, Interventions designed to reduce implicit prejudices and implicit stereotypes in real world contexts: A systematic review. *BMC Psychol.* **7**, 29 (2019).

21. M. B. Vela, A. I. Erondu, N. A. Smith, M. E. Peek, J. N. Woodruff, M. H. Chin, Eliminating explicit and implicit biases in health care: Evidence and research needs. *Annu. Rev. Public Health* **43**, 477–501 (2022).

22. S. K. Calabrese, V. A. Earnshaw, K. Underhill, N. B. Hansen, J. F. Dovidio, The impact of patient race on clinical decisions related to prescribing HIV pre-exposure prophylaxis (PrEP): Assumptions about sexual risk compensation and implications for access. *AIDS Behav.* **18**, 226–240 (2014).

23. K. M. Hoffman, S. Trawalter, J. R. Axt, M. N. Oliver, Racial bias in pain assessment and treatment recommendations, and false beliefs about biological differences between blacks and whites. *Proc. Natl. Acad. Sci. U.S.A.* **113**, 4296–4301 (2016).

24. H. A. Long, D. P. French, J. M. Brooks, Optimising the value of the critical appraisal skills programme (CASP) tool for quality appraisal in qualitative evidence synthesis. *Res. Methods Med. Health Sci.* **1**, 31–42 (2020).

25. S. M. Cheng, C. C. McKinney, A. Hurtado-de-Mendoza, S. Chan, K. D. Graves, Confidence, connection & collaboration: Creating a scalable bias reduction improvement coaching train-the-trainer program to mitigate implicit bias across a medical center. *Teach. Learn. Med.* **36**, 381–398 (2023).

26. C. Okorie-Awé, S. Y. Crawford, L. K. Sharp, B. U. Jaki, M. D. Kachlic, A faculty and staff workshop on microaggression and implicit bias: Knowledge and awareness of student, faculty, and staff experiences. *Curr. Pharm. Teach. Learn.* **13**, 1200–1209 (2021).

27. H. J. Braun, P. S. O'Sullivan, M. N. Dusch, S. Antrum, N. L. Ascher, Improving interprofessional collaboration: Evaluation of implicit attitudes in the surgeon-nurse relationship. *Int. J. Surg.* **13**, 175–179 (2015).

28. J. Sukhera, K. Bertram, S. Hendrikx, M. S. Chisolm, J. Perzhinsky, E. Kennedy, L. Lingard, M. Goldszmidt, Exploring implicit influences on interprofessional collaboration: A scoping review. *J. Interprof. Care* **36**, 716–724 (2022).

29. Rayyan. “Rayyan - AI powered tool for systematic literature reviews” (2021) **36**; www.rayyan.ai/

30. D. A. Reed, D. A. Cook, T. J. Beckman, R. B. Levine, D. E. Kern, S. M. Wright, Association between funding and quality of published medical education research. *JAMA* **298**, 1002–1009 (2007).

31. Critical Appraisal Skills Programme, “CASP - Critical Appraisal Skills Programme”; https://casp-uk.net/casp-tools-checklists/

32. D. A. Cook, R. Hatala, R. Brydges, B. Zendejas, J. H. Szostek, A. T. Wang, P. J. Erwin, S. J. Hamstra, Technology-enhanced simulation for health professions education: A systematic review and meta-analysis. *JAMA* **306**, 978–988 (2011).

33. K. W. Bartlett, P. Strelitz, J. Hawley, R. Sloane, B. B. Staples, Impact of small-group workshop on resident preparedness to provide culturally competent care. *Acad. Pediatr.* **15**, e1–e2 (2015).

34. R. Bernstein, L. Ruffalo, D. Bower, A multielement community medicine curriculum for the family medicine clerkship. *MedEdPORTAL* **12**, 10417 (2016).

35. M. V. Chapman, W. J. Hall, K. Lee, R. Colby, T. Coyne-Beasley, S. Day, E. Eng, A. F.

Lightfoot, Y. Merino, F. M. Siman, T. Thomas, K. Thatcher, K. Payne, Making a difference in medical trainees' attitudes toward Latino patients: A pilot study of an intervention to modify implicit and explicit attitudes. *Soc. Sci. Med.* **199**, 202–208 (2018).

36. L. Corsino, K. Railey, K. Brooks, D. Ostrovsky, S. O. Pinheiro, A. McGhan-Johnson, B. I. Padilla, The impact of racial bias in patient care and medical education: Let's focus on the educator. *MedEdPORTAL* **17**, 11183 (2021).

37. M. DallaPiazza, M. Padilla-Register, M. Dwarakanath, E. Obamedo, J. Hill, M. L. Soto-Greene, Exploring racism and health: An intensive interactive session for medical students. *MedEdPORTAL* **14**, 10783 (2018).

38. D. L. F. Davis, D. Tran-Taylor, E. Imbert, J. O. Wong, C. L. Chou, Start the way you want to finish: An intensive diversity, equity, inclusion orientation curriculum in undergraduate medical education. *J. Med. Educ. Curric. Dev.* **8**, 23821205211000352 (2021).

39. G. Geller, P. A. Watkins, Addressing medical students' negative bias toward patients with obesity through ethics education. *AMA J. Ethics* **20**, E948-E959 (2018).

40. A. C. Gill, Y. Zhou, J. T. Greely, A. D. Beasley, J. Purkiss, M. Juneja, Longitudinal outcomes one year following implicit bias training in medical students. *Med. Teach.* **44**, 744–751 (2022).

41. C. M. Gonzalez, M. Y. Kim, P. R. Marantz, Implicit bias and its relation to health disparities: A teaching program and survey of medical students. *Teach. Learn. Med.* **26**, 64–71 (2014).

42. M. Jindal, R. L. J. Thornton, A. McRae, N. Unaka, T. J. Johnson, K. B. Mistry, Effects of a curriculum addressing racism on pediatric residents' racial biases and empathy. *J. Grad. Med. Educ.* **14**, 407–413 (2022).

43. J. W. Kanter, D. C. Rosen, K. E. Manbeck, H. M. L. Branstetter, A. M. Kuczynski, M. D. Corey, D. W. M. Maitland, M. T. Williams, Addressing microaggressions in racially charged patient-provider interactions: A pilot randomized trial. *BMC Med. Educ.* **20**, 88 (2020).

44. B. Khandalavala, J. Koran-Scholl, J. Geske, Comprehensive obesity education for family medicine residents. *Primer* **4**, 25 (2020).

45. K. Knox, D. Simpson, J. Bidwell, W. Lehmann, Implementing an interprofessional anti-racism training with community partners during a pandemic: Outcomes and recommended strategies. *WMJ.* **120**, S70–S73 (2021).

46. M. Kokas, J. W. Fakhoury, M. Hoffert, S. Whitehouse, M. Van Harn, K. Baker-Genaw, Health care disparities: A practical approach to teach residents about self-bias and patient communication. *J. Racial Ethn. Health Disparities* **6**, 1030–1034 (2019).

47. J. R. Korndorffer, S. M. Wren, C. M. Pugh, M. T. Hawn, From listening to action. *Ann. Surg.* **274**, 921–924 (2021).

48. C. Lelutiu-Weinberger, K. A. Clark, J. E. Pachankis, Mental health provider training to improve LGBTQ competence and reduce implicit and explicit bias: A randomized controlled trial of online and in-person delivery. *Psychol. Sex. Orientat. Gend. Divers.* **10**, 589–599 (2023).

49. K. F. Leslie, S. Sawning, M. A. Shaw, L. J. Martin, R. C. Simpson, J. E. Stephens, V. F. Jones, Changes in medical student implicit attitudes following a health equity curricular intervention. *Med. Teach.* **40**, 372–378 (2018).

50. F. F. Liu, J. Coifman, E. McRee, J. Stone, A. Law, L. Gaias, R. Reyes, C. K. Lai, I. V. Blair, C. Yu, H. Cook, A. R. Lyon, A brief online implicit bias intervention for school mental health clinicians. *Int. J. Environ. Res. Public Health* **19**, 679 (2022).

51. W. H. Lu, P. Baldelli, P. Migdal, R. Iuli, L. Strano-Paul, K. L. Zacharoff, Early refill of an opioid medication: recognizing personal biases through clinical vignettes and OSCEs. *MedEdPORTAL* **18**, 11234 (2022).

52. B. Marr, S. H. Mickey, S. G. Blythe, J. Baruch, The weight of pain: What does a 10 on the pain scale mean? An innovative use of art in medical education to enhance pain management. *J. Pain Symptom Manage.* **57**, 1182–1187 (2019).

53. J. J. Mayfield, E. M. Ball, K. A. Tillery, C. Crandall, J. Dexter, J. M. Winer, Z. M. Bosshardt, J. H. Welch, E. Dolan, E. R. Fancovic, A. I. Nanez, H. De May, E. Finlay, S. M. Lee, C. G. Streed, K. Ashraf, Beyond men, women, or both: A comprehensive, LGBTQ-inclusive, implicit-bias-aware, standardized-patient-based sexual history taking curriculum. *MedEdPORTAL* **13**, 10634 (2017).

54. A. Mendizabal, J. H. Fan, R. S. Price, R. H. Hamilton, Feasibility and effectiveness appraisal of a neurology residency health equities curriculum. *J. Neurol. Sci.* **431**, 120040 (2021).

55. S. C. Nelson, S. Prasad, H. W. Hackman, Training providers on issues of race and racism improve health care equity. *Pediatr. Blood Cancer* **62**, 915–917 (2015).

56. S. Nestorowicz, N. Saks, Addressing bias toward overweight patients: A training program for first-year medical students. *Med. Sci. Educ.* **31**, 1115–1123 (2021).

57. D. Ogunyemi, Defeating unconscious bias: The role of a structured, reflective, and interactive workshop. *J. Grad. Med. Educ.* **13**, 189–194 (2021).

58. P. Poitevien, C. Osman, Tackling implicit and explicit bias through objective structured teaching exercises for faculty. *J. Grad. Med. Educ.* **10**, 353–354 (2018).

59. C. Reddyhough, V. Locke, G. Paulik, Changing healthcare professionals' attitudes towards voice hearers: An education intervention. *Community Ment. Health J.* **57**, 960–964 (2021).

60. M. Ruben, N. S. Saks, Addressing implicit bias in first-year medical students: A longitudinal, multidisciplinary training program. *Med. Sci. Educ.* **30**, 1419–1426 (2020).

61. J. N. Siegelman, C. Woods, B. Salhi, S. Heron, Health care disparities education using the implicit association test. *Med. Educ.* **50**, 1158–1159 (2016).

62. J. Stone, G. B. Moskowitz, C. A. Zestcott, K. J. Wolsiefer, Testing active learning workshops for reducing implicit stereotyping of hispanics by majority and minority group medical students. *Stigma Health* **5**, 94–103 (2020).

63. J. A. Swift, V. Tischler, S. Markham, I. Gunning, C. Glazebrook, C. Beer, R. Puhl, Are anti-stigma films a useful strategy for reducing weight bias among trainee healthcare professionals? Results of a pilot randomized control trial. *Obes. Facts* **6**, 91–102 (2013).

64. K. Terry, N. A. Nickman, S. Mullin, P. Ghule, L. S. Tyler, Implementation of implicit bias awareness and action training in a pharmacy residency program. *Am. J. Health. Syst. Pharm.* **79**, 1929–1937 (2022).

65. C. Traba, A. Jain, K. Pianucci, J. Rosen-Valverde, S. Chen, Down to the last dollar: Utilizing a virtual budgeting exercise to recognize implicit bias. *MedEdPORTAL.* **17**, 11199 (2021).

66. E. Ufomata, K. L. Eckstrand, P. Hasley, K. Jeong, D. Rubio, C. Spagnoletti, Comprehensive internal medicine residency curriculum on primary care of patients who identify as LGBT. *LGBT Health* **5**, 375–380 (2018).

67. E. Vaimberg, L. Demers, E. Ford, M. Sabatello, B. Stevens, S. Dasgupta, Project inclusive genetics: Exploring the impact of patient-centered counseling training on physical disability bias in the prenatal setting. *PLOS ONE* **16**, e0255722 (2021).

68. N. N. Wijayatunga, D. Bailey, S. S. Klobodu, J. A. Dawson, K. Knight, E. J. Dhurandhar, A short, attribution theory-based video intervention does not reduce weight bias in a nationally representative sample of registered dietitians: A randomized trial. *Int. J. Obes. (Lond)* **45**, 787–794 (2021).

69. D. Wu, L. Saint-Hilaire, A. Pineda, D. Hessler, G. W. Saba, R. Salazar, N. Olayiwola, The efficacy of an antioppression curriculum for health professionals. *Fam. Med.* **51**, 22–30 (2019).

70. A. J. Zeidan, U. G. Khatri, J. Aysola, F. S. Shofer, M. Mamtani, K. R. Scott, L. W. Conlon, B. L. Lopez, Implicit bias education and emergency medicine training: Step one? Awareness. *AEM Educ. Train.* **3**, 81–85 (2019).

71. M. E. K. Amin, Addressing cultural competence and bias in treating migrant workers in pharmacies: Pharmacy students learning and changing norms. *Res. Soc. Adm. Pharm.* **18**,

3362–3368 (2022).

72. M. E. Archambault, J. A. Van Rhee, G. S. Marion, S. J. Crandall, Utilizing implicit association testing to promote awareness of biases regarding age and disability. *J. Physician Assist. Educ.* **19**, 20–26 (2008).

73. A. N. Chary, M. F. Molina, F. Z. Dadabhoy, E. C. Manchanda, Addressing racism in medicine through a resident-led health equity retreat. *West. J. Emerg. Med.* **22**, 41–44 (2020).

74. M. H. Chin, M. M. Aburmishan, M. Zhu, Standup comedy principles and the personal monologue to explore interpersonal bias: Experiential learning in a health disparities course. *BMC Med. Educ.* **22**, 80 (2022).

75. L. Clementz, M. McNamara, N. M. Burt, M. Sparks, M. K. Singh, Starting with Lucy: Focusing on human similarities rather than differences to address health care disparities. *Acad. Med.* **92**, 1259–1263 (2017).

76. M. DallaPiazza, M. S. Ayyala, M. L. Soto-Greene, Empowering future physicians to advocate for health equity: A blueprint for a longitudinal thread in undergraduate medical education. *Med. Teach.* **42**, 806–812 (2020).

77. E. Díaz, T. Armah, C. T. Linse, A. Fiskin, A. Jordan, J. Hafler, Novel brief cultural psychiatry training for residents. *Acad. Psychiatry.* **40**, 366–368 (2016).

78. J. Ellison, C. Gunther, M. B. Campbell, R. English, C. Lazarus, Critical consciousness as a framework for health equity-focused peer learning. *MedEdPORTAL* **17**, 11145 (2021).

79. H. F. Fitterman-Harris, J. S. Vander Wal, Weight bias reduction among first-year medical students: A quasi-randomized, controlled trial. *Clin. Obes.* **11**, e12479 (2021).

80. E. Gatewood, C. Broholm, J. Herman, C. Yingling, Making the invisible visible: Implementing an implicit bias activity in nursing education. *J. Prof. Nurs.* **35**, 447–451 (2019).

81. C. M. Gonzalez, A. D. Fox, P. R. Marantz, The evolution of an elective in health disparities and advocacy: Description of instructional strategies and program evaluation. *Acad. Med.* **90**, 1636–1640 (2015).

82. A. K. Hughes, C. Luz, D. Hall, P. Gardner, C. W. Hennessey, L. Lammers, Transformative theatre: A promising educational tool for improving health encounters with LGBT older adults. *Gerontol. Geriatr. Educ.* **37**, 292–306 (2016).

83. V. Kerrigan, N. Lewis, A. Cass, M. Hefler, A. P. Ralph, "How can I do more?" Cultural awareness training for hospital-based healthcare providers working with high aboriginal caseload. *BMC Med. Educ.* **20**, 173 (2020).

84. K. Matharu, J. F. Shapiro, R. R. Hammer, R. L. Kravitz, M. D. Wilson, F. T. Fitzgerald, Reducing obesity prejudice in medical education. *Educ. Health* **27**, 231–237 (2014).

85. P. A. McElfish, C. R. Long, B. Rowland, S. Moore, R. Wilmoth, B. Ayers, Improving culturally appropriate care using a community-based participatory research approach: Evaluation of a multicomponent cultural competency training program, Arkansas, 2015-2016. *Prev. Chronic Dis.* **14**, E62 (2017).

86. M. Medlock, A. Weissman, S. S. Wong, A. Carlo, M. Zeng, C. Borba, M. Curry, D. Shtasel, Racism as a unique social determinant of mental health: Development of a didactic curriculum for psychiatry residents. *MedEdPORTAL* **13**, 10618 (2017).

87. T. A. Mullett, S. N. Rooholamini, C. Gilliam, H. McPhillips, H. M. Grow, Description of a novel curriculum on equity, diversity and inclusion for pediatric residents. *J. Natl. Med. Assoc.* **113**, 616–625 (2022).

88. J. Perdomo, D. Tolliver, H. Hsu, Y. He, K. A. Nash, S. Donatelli, C. Mateo, C. Akagbosu, F. Alizadeh, A. Power-Hays, T. Rainier, D. J. Zheng, C. J. Kistin, R. J. Vinci, C. D. Michelson, Health equity rounds: An interdisciplinary case conference to address implicit bias and structural racism for faculty and trainees. *MedEdPORTAL* **15**, 10858 (2019).

89. J. Raney, R. Pal, T. Lee, S. R. Saenz, D. Bhushan, P. Leahy, C. Johnson, C. Kapphahn, M. A.

Gisondi, K. Hoang, Words matter: An antibias workshop for health care professionals to reduce stigmatizing language. *MedEdPORTAL* **17**, 11115 (2021).

90. N. Rodriguez, E. Kintzer, J. List, M. Lypson, J. H. Grochowalski, P. R. Marantz, C. M. Gonzalez, Implicit bias recognition and management: Tailored instruction for faculty. *J. Natl. Med. Assoc.* **113**, 566–575 (2021).

91. P. L. Schultz, J. Baker, Teaching strategies to increase nursing student acceptance and management of unconscious bias. *J. Nurs. Educ.* **56**, 692–696 (2017).

92. R. Steed, Attitudes and beliefs of occupational therapists participating in a cultural competency workshop. *Occup. Ther. Int.* **17**, 142–151 (2010).

93. R. Steed, The effects of an instructional intervention on racial attitude formation in occupational therapy students. *J. Transcult. Nurs.* **25**, 403–409 (2014).

94. J. Sukhera, K. Miller, C. Scerbo, A. Milne, R. Lim, C. Watling, Implicit stigma recognition and management for health professionals. *Acad. Psychiatry.* **44**, 59–63 (2020).

95. G. S. Tajeu, L. Juarez, J. H. Williams, J. Halanych, I. Stepanikova, A. A. Agne, J. Stone, A. L. Cherrington, Development of a multicomponent intervention to decrease racial bias among healthcare staff. *J. Gen. Intern. Med.* **37**, 1970–1979 (2022).

96. S. Wasmuth, K. Pritchard, C. Milton, E. Smith, A mixed-method analysis of community-engaged theatre illuminates black women’s experiences of racism and addresses healthcare inequities by targeting provider bias. *Inquiry* **57**, 46958020976255 (2020).

97. T. White-Davis, J. Edgoose, J. S. Brown Speights, K. Fraser, J. M. Ring, J. Guh, G. W. Saba, Addressing racism in medical education: An interactive training module. *Fam. Med.* **50**, 364–368 (2018).

98. A. Zeidan, A. Tiballi, M. Woodward, I. M. Di Bartolo, Targeting implicit bias in medicine: Lessons from art and archaeology. *West. J. Emerg. Med.* **21**, 1–3 (2020).

99. N. B. Collier, L. Taylor, Fostering awareness of implicit bias using an adapted visual thinking strategy and reflection. *J. Physician Assist. Educ.* **33**, 145–147 (2022).

100. E. Geiser, L. V. Schilter, J. M. Carrier, C. Clair, J. Schwarz, Reflexivity as a tool for medical students to identify and address gender bias in clinical practice: A qualitative study. *Patient Educ. Couns.* **105**, 3521–3528 (2022).

101. C. M. Gonzalez, S. Nava, J. List, A. Liguori, P. R. Marantz, How assumptions and preferences can affect patient care: An introduction to implicit bias for first-year medical students. *MedEdPORTAL* **17**, 11162 (2021).

102. A. L. Holm, M. Rowe Gorosh, M. Brady, D. White-Perkins, Recognizing privilege and bias: An interactive exercise to expand health care providers' personal awareness. *Acad. Med.* **92**, 360–364 (2017).

103. R. Khazanchi, H. Keeler, S. Strong, E. R. Lyden, P. Davis, B. K. Grant, J. R. Marcelin, Building structural competency through community engagement. *Clin. Teach.* **18**, 535–541 (2021).

104. B. McMichael, A. Nickel, E. A. Duffy, L. Skjefte, L. Lee, P. Park, S. C. Nelson, S. Puumala, A. P. Kharbanda, The impact of health equity coaching on patient's perceptions of cultural competency and communication in a pediatric emergency department: An intervention design. *J. Patient Exp.* **6**, 257–264 (2019).

105. M. D. Sherman, J. Ricco, S. C. Nelson, S. J. Nezhad, S. Prasad, Implicit bias training in a residency program: Aiming for enduring effects. *Fam. Med.* **51**, 677–681 (2019).

106. O. Solá, C. Marquez, Integrating social determinants of health into clinical training during the COVID-19 pandemic. *Primer* **4**, 28 (2020).

107. C. R. Teal, R. E. Shada, A. C. Gill, B. M. Thompson, E. Frugé, G. B. Villarreal, P. Haidet, When best intentions aren't enough: Helping medical students develop strategies for

managing bias about patients. *J. Gen. Intern. Med.* **25** (Suppl. 2), S115-S118 (2010).

108. T. J. West, K. Loomer, T. R. Wyatt, How diverse is your universe? An activity for students to reflect on ethnoracial diversity during orientation. *MedEdPORTAL* **15**, 10840 (2019).

109. A. A. White, H. J. Logghe, D. A. Goodenough, L. L. Barnes, A. Hallward, I. M. Allen, D. W. Green, E. Krupat, R. Llerena-Quinn, Self-awareness and cultural identity as an effort to reduce bias in medicine. *J. Racial Ethn. Health Disparities* **5**, 34–49 (2018).
