# Supplementary material for: The nature and validity of implicit bias training for health care providers and trainees: A systematic review
Source: Sci Adv. 2024 Aug 14;10(33):eado5957. doi: 10.1126/sciadv.ado5957 (PMC11323883; doi:10.1126/sciadv.ado5957)
Supplement: Supplementary file 2 — Table S2 [file sciadv.ado5957_table_s2.zip › ado5957_Table_S2.docx]

**Table S2. Additional studies that were excluded during full-text screening.**

| **Reference** | **Reason for Exclusion** |
| --- | --- |
| Aboshaiqah et al., 2017 | Training not focused on implicit bias |
| Abou Baker et al., 2021 | Conference abstract only (no full text) |
| Abraham & Torner, 2021 | Training not focused on healthcare providers |
| Acholonu et al., 2020 | Training focused on addressing microaggressions toward peers |
| Adames et al., 2011 | Study was not a peer-reviewed empirical paper |
| Adams, 2021 | Study was not a peer-reviewed empirical paper |
| Adams & Kaufman, 2011 | Study was not a peer-reviewed empirical paper |
| Adelman et al., 2007 | Training not focused on implicit bias |
| Agboola, 2021 | Study was not a peer-reviewed empirical paper |
| Al-Mateen, 2017 | Conference abstract only (no full text) |
| Ali & Halim, 2014 | Study was not a peer-reviewed empirical paper |
| Allen et al., 2013 | Training not focused on implicit bias |
| Alspach, 2018 | Study was not a peer-reviewed empirical paper |
| Andrews & Hay, 2018 | Conference abstract only (no full text) |
| Ang et al., 2020 | Training not focused on implicit bias |
| Anonymous, 2008 | Training not focused on implicit bias |
| Ansari et al., 2020 | Study did not assess relevant outcome |
| Ard et al., 2021 | Conference abstract only (no full text) |
| Arif & Schlotfeldt, 2021 | Study was not a peer-reviewed empirical paper |
| Ascoli et al., 2012 | Training not focused on implicit bias |
| Ashford et al., 2018 | Training not focused on healthcare providers |
| Ashurst, 2019 | Study was not a peer-reviewed empirical paper |
| Astudillo & Noulas, 2022 | Conference abstract only (no full text) |
| Avant & Gillespie, 2019 | Study did not assess relevant outcome |
| Avant et al., 2018 | Training not focused on implicit bias |
| Bahrke et al., 2014 | Training not focused on implicit bias |
| Baig et al., 2014 | Conference abstract only (no full text) |
| Bakke et al., 2014 | Study was not a peer-reviewed empirical paper |
| Ball et al., 2021 | Conference abstract only (no full text) |
| Bansal, 2016 | Study was not a peer-reviewed empirical paper |
| Basham, 2004 | Study was not a peer-reviewed empirical paper |
| Bayar et al., 2009 | Training not focused on implicit bias |
| Beam et al., 2016 | Conference abstract only (no full text) |
| Bellack, 2015 | Study was not a peer-reviewed empirical paper |
| Bello et al., 2022 | Conference abstract only (no full text) |
| Bennett et al., 2019 | Training not focused on implicit bias |
| Bennett et al., 2007 | Study was not a peer-reviewed empirical paper |
| Benoit et al., 2020 | Training not focused on implicit bias |
| Bey et al., 2022 | Conference abstract only (no full text) |
| Bheenuck et al., 2007 | Training not focused on implicit bias |
| Birtel & Crisp, 2012 | Training not focused on healthcare providers |
| Borowsky et al., 2021 | Training not focused on implicit bias |
| Boscardin, 2015 | Study was not a peer-reviewed empirical paper |
| Bowman et al., 2011 | Study did not assess relevant outcome |
| Boykins, 2018 | Study did not assess relevant outcome |
| Brammer et al., 2022 | Study did not assess relevant outcome |
| Braun et al., 2017 | Training not focused on implicit bias |
| Bridgeman-Bunyoli et al., 2015 | Training not focused on implicit bias |
| Bristol et al., 2018 | Training not focused on implicit bias |
| Brondani & Paterson, 2011 | Training not focused on implicit bias |
| Brooks et al., 2016 | Training not focused on implicit bias |
| Broomes Williamson, 2020 | Conference abstract only (no full text) |
| Brown & Bright, 2017 | Training not focused on implicit bias |
| Browne et al., 2018 | Training not focused on implicit bias |
| Buchs & Mulitalo, 2016 | Study was not a peer-reviewed empirical paper |
| Burbank et al., 2006 | Training not focused on implicit bias |
| Burgess et al., 2017 | Study was not a peer-reviewed empirical paper |
| Burns & McNally, 2021 | Conference abstract only (no full text) |
| Butler-Byrd et al., 2006 | Training not focused on implicit bias |
| Caffrey et al., 2016 | Training not focused on implicit bias |
| Cahn, 2017 | Training not focused on healthcare providers |
| Calardo et al., 2022 | Training not focused on implicit bias |
| Carbonneau, 2021 | Dissertation |
| Carey et al., 2022 | Conference abstract only (no full text) |
| Carnes et al., 2021 | Study did not assess relevant outcome |
| Cartz Piver et al., 2020 | Conference abstract only (no full text) |
| Castillo et al., 2007 | Training not focused on implicit bias |
| Centola et al., 2021 | Training not focused on implicit bias |
| Chang & Lee, 2018 | No English version available |
| Chao, 2012 | Training not focused on implicit bias |
| Chao, 2013 | Training not focused on implicit bias |
| Chao et al., 2011 | Training not focused on implicit bias |
| Charles et al., 2005 | Training not focused on implicit bias |
| Chary et al., 2020 | Training not focused on implicit bias |
| Chew et al., 2016 | Study did not assess relevant outcome |
| Chow et al., 2019 | Training not focused on implicit bias |
| Chrobot-Mason, 2012 | Training not focused on healthcare providers |
| Clarke et al., 2021 | Study was not a peer-reviewed empirical paper |
| Cleeves et al., 2019 | Conference abstract only (no full text) |
| Clement et al., 2012 | Training not focused on implicit bias |
| Clendon, 2020 | Study was not a peer-reviewed empirical paper |
| Clevel et al., 2020 | Duplicate identified at this stage of screening |
| Closson & Rhodes, 2011 | Conference abstract only (no full text) |
| Cobo & Pfeiffer, 2015 | Duplicate identified at this stage of screening |
| Cooper et al., 2018 | Training not focused on implicit bias |
| Costa et al., 2016 | Training not focused on implicit bias |
| Crabtree & Mack, 2010 | Conference abstract only (no full text) |
| Crampton et al., 2003 | Training not focused on implicit bias |
| Cravens et al., 2016 | Study did not assess relevant outcome |
| Crawford, 2020 | Study was not a peer-reviewed empirical paper |
| Cropper-Williams, 2018 | Conference abstract only (no full text) |
| Crowe, et al., 2021 | Conference abstract only (no full text) |
| Cucco, 2020 | Training not focused on implicit bias |
| Cutler et al., 2012 | Training not focused on implicit bias |
| Daniel et al., 2017 | Study did not assess relevant outcomes |
| Davtyan et al., 2020 | Training not focused on implicit bias |
| Debiasi & Selleck, 2017 | Training not focused on implicit bias |
| DeRicco & Sciarra, 2005 | Training not focused on implicit bias |
| Desai et al., 2021 | Training not focused on implicit bias |
| Dhanani et al., 2022 | Training not focused on implicit bias |
| Diachun et al., 2010 | Training not focused on implicit bias |
| Diaz Del Carpio et al., 2018 | Conference abstract only (no full text) |
| DiBrito et al., 2019 | Study did not assess relevant outcomes |
| Dickson et al., 2010 | Training not focused on implicit bias |
| Diedrichs & Barlow, 2011 | Training not focused on healthcare providers |
| Dion, 2019 | Training not focused on implicit bias |
| Doucette et al., 2015 | Training not focused on implicit bias |
| Doyle & Cruickshank, 2012 | Conference abstract only (no full text) |
| Dupre & Goodgold, 2007 | Training not focused on implicit bias |
| Eberly et al., 2021 | Study did not assess relevant outcomes |
| Echeverri & Dise, 2017 | Training not focused on implicit bias |
| Edgoose et al., 2021 | Study did not assess relevant outcomes |
| Edwards et al., 2021 | Conference abstract only (no full text) |
| Ellis et al., 2021 | Conference abstract only (no full text) |
| Ertl et al., 2020 | Study did not assess relevant outcomes |
| Etengoff, 2020 | Training not focused on implicit bias |
| Evans & McCaughan, 2020 | Study was not a peer-reviewed empirical paper |
| Farmer et al., 2011 | Conference abstract only (no full text) |
| Fisher et al., 2017 | Study was not a peer-reviewed empirical paper |
| Fisher et al., 2020 | Conference abstract only (no full text) |
| Fisher et al., 2021 | Training focused on addressing microaggressions toward peers |
| Fitzgerald, 2021 | Study was not a peer-reviewed empirical paper |
| Fitzgerald et al., 2008 | Training not focused on implicit bias |
| Flanagan et al., 2016 | Training not focused on implicit bias |
| Flatt-Fultz & Phillips, 2012 | Training not focused on implicit bias |
| Fleming et al., 2022 | Conference abstract only (no full text) |
| Fletcher & Akakpo, 2020 | Training not focused on healthcare providers |
| Foster & Clark, 2015 | Training not focused on implicit bias |
| Friday, 2015 | Training not focused on implicit bias |
| Friedman & VanPuymbrouck, 2021 | Training not focused on implicit bias |
| Friedrich et al., 2013 | Training not focused on implicit bias |
| Fruhauf et al., 2004 | Training not focused on implicit bias |
| Fuchs et al., 2021 | Conference abstract only (no full text) |
| Galletly et al., 2011 | Training not focused on implicit bias |
| Geibel et al., 2017 | Training not focused on implicit bias |
| Genao et al., 2009 | Training not focused on implicit bias |
| Gendron et al., 2021 | Training not focused on implicit bias |
| Gholamzadeh et al., 2018 | Training not focused on implicit bias |
| Gillespie et al., 2022 | Conference abstract only (no full text) |
| Gillespie et al., 2017 | Training not focused on implicit bias |
| Gilliam et al., 2020 | Conference abstract only (no full text) |
| Gillian-Daniel et al., 2020 | Study was not a peer-reviewed empirical paper |
| Gitlin, 2018 | Conference abstract only (no full text) |
| Gleeson et al., 2021 | Conference abstract only (no full text) |
| Gogineni et al., 2022 | Conference abstract only (no full text) |
| Golden et al., 2010 | Training not focused on implicit bias |
| Gonzales et al., 2010 | Training not focused on implicit bias |
| Gonzalez et al., 2022 | Conference abstract only (no full text) |
| Gonzalez et al., 2018 | Conference abstract only (no full text) |
| Gonzalez et al., 2020 | Study did not assess relevant outcomes |
| Gonzalez et al., 2021 | Study did not assess relevant outcomes |
| Gordon Perue et al., 2021 | Study was not a peer-reviewed empirical paper |
| Greene, 2016 | Training not focused on implicit bias |
| Griner et al., 2022 | Training not focused on healthcare providers |
| Guh et al., 2019 | Training not focused on implicit bias |
| Guh et al., 2020 | Study did not assess relevant outcomes |
| Gutierrez et al., 2014 | Training not focused on healthcare providers |
| Guzder et al., 2013 | Training not focused on implicit bias |
| Hackett et al., 2018 | Conference abstract only (no full text) |
| Hagopian et al., 2018 | Training not focused on implicit bias |
| Halloran, 2009 | Training not focused on implicit bias |
| Hamel et al., 2022 | Conference abstract only (no full text) |
| Hamilton-Mason & Schneider, 2018 | Training not focused on implicit bias |
| Hannah & Carpenter-Song, 2013 | Training not focused on implicit bias |
| Hardeman et al., 2018 | Training not focused on implicit bias |
| Harris et al., 2016 | Training not focused on implicit bias |
| Harrison-Bernard et al., 2020 | Training not focused on healthcare providers |
| Harrison-Bernard et al., 2018 | Duplicate identified at this stage of screening |
| Hawke et al., 2014 | Training not focused on implicit bias |
| Hernandez et al., 2013 | Study did not assess relevant outcomes |
| Heuer et al., 2020 | Training not focused on implicit bias |
| Hillbrand et al., 2008 | Training not focused on implicit bias |
| Hinners & Potter, 2006 | Training not focused on implicit bias |
| Ho et al., 2008 | Training not focused on implicit bias |
| Hodgins et al., 2019 | Conference abstract only (no full text) |
| Hofmeister & Soprych, 2017 | Study did not assess relevant outcomes |
| Holman et al., 2011 | Conference abstract only (no full text) |
| Holroyd et al., 2009 | Training not focused on implicit bias |
| Horst et al., 2019 | Training not focused on implicit bias |
| Hughes & Hood, 2007 | Training not focused on implicit bias |
| Hui et al., 2020 | Study was not a peer-reviewed empirical paper |
| Ilyenkova et al., 2012 | Training not focused on implicit bias |
| Isom, 2021 | Dissertation |
| Jabson et al., 2016 | Training not focused on implicit bias |
| Jackson et al., 2011 | Conference abstract only (no full text) |
| Jackson et al., 2011 | Conference abstract only (no full text) |
| Jackson, 2015 | Training not focused on implicit bias |
| Jahnke et al., 2015 | Training not focused on implicit bias |
| Janouskova et al., 2019 | Conference abstract only (no full text) |
| Jansen & Morse, 2004 | Training not focused on implicit bias |
| Jarris et al., 2012 | Training not focused on implicit bias |
| Javier et al., 2022 | Training not focused on healthcare providers |
| Jaworsky et al., 2017 | Training not focused on implicit bias |
| Jeste et al., 2018 | Training not focused on implicit bias |
| Jones & Forhan, 2021 | Training not focused on implicit bias |
| Kaatz et al., 2017 | Conference abstract only (no full text) |
| Kaf et al., 2011 | Training not focused on implicit bias |
| Kallianos et al., 2019 | Study did not assess relevant outcomes |
| Kantor & Myers, 2006 | Training not focused on implicit bias |
| Karayigit & Ozier, 2021 | Training not focused on implicit bias |
| Kassam et al., 2011 | Training not focused on implicit bias |
| Khan, 2019 | Conference abstract only (no full text) |
| Khidir et al., 2022 | Conference abstract only (no full text) |
| Kidd et al., 2016 | Training not focused on implicit bias |
| Kim et al., 2019 | Study was not a peer-reviewed empirical paper |
| Kistin, 2015 | Study was not a peer-reviewed empirical paper |
| Klein & Nakhai, 2016 | Study was not a peer-reviewed empirical paper |
| Kleinschmidt et al., 2020 | Conference abstract only (no full text) |
| Kliot et al., 2021 | Conference abstract only (no full text) |
| Knoeckel et al., 2018 | Conference abstract only (no full text) |
| Koch et al., 2021 | Training not focused on implicit bias |
| Kogan & Schoenfeld-Tacher, 2018 | Training not focused on healthcare providers |
| Kohrt et al., 2018 | Study was not a peer-reviewed empirical paper |
| Kushner et al., 2014 | Training not focused on implicit bias |
| Lam et al., 2015 | Training not focused on implicit bias |
| Lapshin et al., 2006 | Training not focused on implicit bias |
| Lawlis et al., 2022 | Conference abstract only (no full text) |
| Lee et al., 2018 | Training not focused on implicit bias |
| Lenes et al., 2020 | Training not focused on implicit bias |
| Lewis et al., 2015 | Training not focused on implicit bias |
| Li et al., 2015 | Training not focused on implicit bias |
| Li et al., 2013a | Training not focused on implicit bias |
| Li et al., 2013b | Training not focused on implicit bias |
| Li et al., 2017 | Training not focused on healthcare providers |
| Lie et al., 2006 | Training not focused on implicit bias |
| Lightfoot et al., 2015 | Conference abstract only (no full text) |
| Lincoln et al., 2008 | Study did not assess relevant outcomes |
| Lohiniva et al., 2016 | Training not focused on implicit bias |
| Loignon et al., 2014 | Training not focused on implicit bias |
| Long et al., 2021 | Training not focused on implicit bias |
| Lopez-Villegas et al., 2020 | Conference abstract only (no full text) |
| Lucas, 2018 | Conference abstract only (no full text) |
| Lund et al., 2021 | Study was not a peer-reviewed empirical paper |
| Madianos et al., 2005 | Training not focused on implicit bias |
| Madzima & Crawford, 2021 | Conference abstract only (no full text) |
| Magliano et al., 2014 | Training not focused on implicit bias |
| Magliano et al., 2016 | Training not focused on healthcare providers |
| Mahmoud et al., 2018 | Training not focused on implicit bias |
| Malott & de Zaid, 2007 | Training not focused on implicit bias |
| Marlow et al., 2015 | Training not focused on implicit bias |
| Martinez et al., 2016 | Conference abstract only (no full text) |
| Martinez et al., 2017 | Conference abstract only (no full text) |
| Martinez et al., 2022 | Study was not a peer-reviewed empirical paper |
| Martinez-Martinez et al., 2019 | Training not focused on implicit bias |
| Masse et al., 2020 | Training not focused on implicit bias |
| Masters et al., 2019 | Study was not a peer-reviewed empirical paper |
| Mayers, 2007 | Training not focused on implicit bias |
| McCave et al., 2019 | Training not focused on implicit bias |
| McCleary-Gaddy & Scales, 2019 | Study was not a peer-reviewed empirical paper |
| McClinton & Laurencin, 2020 | Study was not a peer-reviewed empirical paper |
| McCool et al., 2006 | Training not focused on implicit bias |
| McCray, 2021 | Training not focused on implicit bias |
| McDowell et al., 2003 | Training not focused on implicit bias |
| McGervey et al., 2020 | Conference abstract only (no full text) |
| McKinley et al., 2019 | Training focused on addressing microaggressions toward peers |
| McQuade et al., 2021 | Study did not assess relevant outcomes |
| Meltzer et al., 2013 | Training not focused on implicit bias |
| Merritt & Rougas, 2018 | Training not focused on implicit bias |
| Mette & Hanze, 2021 | Training not focused on implicit bias |
| Michaels et al., 2014 | Training not focused on implicit bias |
| Mistler et al., 2011 | Study did not assess relevant outcomes |
| Mittal et al., 2020 | Training not focused on implicit bias |
| Mkandawire-Valhmu et al., 2019 | Training not focused on implicit bias |
| Molina et al., 2020 | Study was not a peer-reviewed empirical paper |
| Molloy et al., 2016 | Training not focused on implicit bias |
| Moriello et al., 2005 | Training not focused on implicit bias |
| Morris et al., 2018 | Study was not a peer-reviewed empirical paper |
| Mueller, 2022 | Dissertation |
| Muenks, 2022 | Conference abstract only (no full text) |
| Munoz et al., 2019 | Training not focused on implicit bias |
| Murray-García et al., 2022 | Training not focused on implicit bias |
| Muzyk et al., 2020 | Training not focused on implicit bias |
| Nadan, 2016 | Study was not a peer-reviewed empirical paper |
| Nadkarni et al., 2020 | Conference abstract only (no full text) |
| Nagasawa et al., 2021 | Study was not a peer-reviewed empirical paper |
| Narayanasamy, 2014 | Study was not a peer-reviewed empirical paper |
| Nelson, 2016 | Study did not assess relevant outcomes |
| Ng et al., 2020 | Training not focused on implicit bias |
| Ng et al., 2010 | Training not focused on implicit bias |
| Nguyen et al., 2012 | Training not focused on implicit bias |
| Nickel et al., 2019 | Training not focused on implicit bias |
| Nnoromele et al., 2021 | Conference abstract only (no full text) |
| Noone, 2022 | Training not focused on implicit bias |
| Norlock et al., 2014 | Conference abstract only (no full text) |
| Nova et al., 2013 | Training not focused on implicit bias |
| Nyblade et al., 2020 | Training not focused on implicit bias |
| O Carroll & O'Reilly, 2019 | Training not focused on implicit bias |
| O' Connor et al., 2013 | Training not focused on implicit bias |
| O'Brien et al., 2010 | Training not focused on healthcare providers |
| O'Connor et al., 2019 | Study was not a peer-reviewed empirical paper |
| O'Neill et al., 2016 | Training not focused on implicit bias |
| O'Reilly et al., 2010 | Training not focused on implicit bias |
| Ogilvie et al., 2021 | Study did not assess relevant outcomes |
| Okorie-Awé et al., 2021 | Study did not assess relevant outcomes |
| Okubanjo & Lovell, 2017 | Conference abstract only (no full text) |
| Oliver et al., 2021 | Training not focused on implicit bias |
| Olveczky et al., 2019 | Training focused on addressing microaggressions toward peers |
| Omori et al., 2012 | Study did not assess relevant outcomes |
| Sandoval et al., 2021 | Conference abstract only (no full text) |
| Owen et al., 2018 | Training not focused on implicit bias |
| Ozcan Edeer & Rust, 2022 | Training not focused on implicit bias |
| Padilla & Garcia, 2022 | Conference abstract only (no full text) |
| Pai et al., 2021 | Training focused on addressing microaggressions toward peers |
| Paniagua et al., 2010 | Training not focused on implicit bias |
| Papish et al., 2013 | Training not focused on implicit bias |
| Paroz et al., 2014 | Training not focused on implicit bias |
| Paroz et al., 2014 | Study was not a peer-reviewed empirical paper |
| Patel et al., 2021 | Conference abstract only (no full text) |
| Patrick et al., 2011 | Study did not assess relevant outcomes |
| Paul et al., 2006 | Training not focused on implicit bias |
| Paulsen et al., 2019 | Conference abstract only (no full text) |
| Pearce, 2017 | Study did not assess relevant outcomes |
| Pearson, 2003 | Training not focused on implicit bias |
| Pekcetin et al., 2021 | Training not focused on implicit bias |
| Pena Dolhun et al., 2003 | Training not focused on implicit bias |
| Peralta et al., 2018 | Conference abstract only (no full text) |
| Perry, 2012 | Dissertation |
| Person & Bucuvalas, 2021 | Conference abstract only (no full text) |
| Pham et al., 2019 | Conference abstract only (no full text) |
| Phelan et al., 2017 | Study did not assess relevant outcomes |
| Phelan et al., 2015 | Study did not assess relevant outcomes |
| Pieterse, 2009 | Training not focused on implicit bias |
| Pisal et al., 2007 | Training not focused on implicit bias |
| Pittman et al., 2010 | Training not focused on implicit bias |
| Poirier et al., 2009 | Training not focused on implicit bias |
| Pomarede & Capara, 2007 | Conference abstract only (no full text) |
| Poustchi et al., 2013 | Training not focused on implicit bias |
| Power et al., 2016 | Training not focused on implicit bias |
| Pribbenow et al., 2021 | Training not focused on healthcare providers |
| Price et al., 2020 | Study did not assess relevant outcomes |
| Price, 2022 | Dissertation |
| Pryce et al., 2019 | Conference abstract only (no full text) |
| Rahman et al., 2020 | Conference abstract only (no full text) |
| Ramsdell, 2019 | Conference abstract only (no full text) |
| Rastegar et al., 2004 | Training not focused on implicit bias |
| Rathod et al., 2010 | Training not focused on implicit bias |
| Redmond et al., 2016 | Conference abstract only (no full text) |
| Reed, 2021 | Training not focused on implicit bias |
| Reilly et al., 2013 | Training not focused on implicit bias |
| Reliford et al., 2022 | Study was not a peer-reviewed empirical paper |
| Reliford et al., 2021 | Conference abstract only (no full text) |
| Ries, 2019 | Study was not a peer-reviewed empirical paper |
| Rimmer, 2016 | Study was not a peer-reviewed empirical paper |
| Roberts et al., 2011 | Training not focused on implicit bias |
| Roberts et al., 2006 | Training not focused on implicit bias |
| Roberts et al., 2008 | Training not focused on implicit bias |
| Roberts, 2021 | Study was not a peer-reviewed empirical paper |
| Roberts & Aida Farhana, 2010 | Training not focused on implicit bias |
| Rodriguez et al., 2021 | Duplicate identified at this stage of screening |
| Rogovin, 2017 | Conference abstract only (no full text) |
| Ross & Lypson, 2014 | Training not focused on implicit bias |
| Roswell et al., 2020 | Training not focused on implicit bias |
| Rothman et al., 2012 | Training not focused on implicit bias |
| Ruiz, 2011 | Training not focused on implicit bias |
| Sabin et al., 2020 | Conference abstract only (no full text) |
| Sabin et al., 2010 | Conference abstract only (no full text) |
| Sadau & Capeles, 2019 | Study was not a peer-reviewed empirical paper |
| Saetermoe et al., 2017 | Training focused on addressing microaggressions toward peers |
| Safdieh et al., 2019 | Study was not a peer-reviewed empirical paper |
| Salahou et al., 2021 | Study was not a peer-reviewed empirical paper |
| Samberg et al., 2021 | Conference abstract only (no full text) |
| Samberg et al., 2021 | Conference abstract only (no full text) |
| Sanchez & Aysola, 2018 | Conference abstract only (no full text) |
| Sandoval et al., 2020 | Conference abstract only (no full text) |
| Sangganjanavanich et al., 2011 | Conference abstract only (no full text) |
| Sarabia-Cobo & Castanedo Pfeiffer, 2015 | Training not focused on implicit bias |
| Sasaki, 2008 | Dissertation |
| Schaeffer & Lupton, 2017 | Conference abstract only (no full text) |
| Schaeffer et al., 2020 | Conference abstract only (no full text) |
| Schenner et al., 2011 | No English version available |
| Schim et al., 2006 | Training not focused on implicit bias |
| Schmetzer et al., 2008 | Training not focused on implicit bias |
| Schneider et al., 2022 | Conference abstract only (no full text) |
| Schwartz et al., 2020 | Training not focused on healthcare providers |
| Scott et al., 2003 | Study was not a peer-reviewed empirical paper |
| Selig et al., 2006 | Training not focused on implicit bias |
| Serra Galceran et al., 2013 | No English version available |
| Shapiro et al., 2006 | Training not focused on implicit bias |
| Sharon et al., 2014 | Study was not a peer-reviewed empirical paper |
| Shen et al., 2014 | Training not focused on implicit bias |
| Shteinlukht et al., 2016 | Study was not a peer-reviewed empirical paper |
| Shteinlukht et al., 2016 | Study was not a peer-reviewed empirical paper |
| Shultz & Skorcz, 2012 | Training not focused on implicit bias |
| Shutak, 2017 | Study was not a peer-reviewed empirical paper |
| Siegelman et al., 2015 | Study was not a peer-reviewed empirical paper |
| Siem et al., 2021 | Training not focused on healthcare providers |
| Sikora, 2006 | Training not focused on implicit bias |
| Simpson et al., 2022 | Training not focused on implicit bias |
| Singaravelu et al., 2011 | Conference abstract only (no full text) |
| Singer et al., 2019 | Training not focused on implicit bias |
| Singh, 2019 | Training not focused on implicit bias |
| Sir & Schoon, 2017 | Conference abstract only (no full text) |
| Sirotin et al., 2013 | Conference abstract only (no full text) |
| Smith et al., 2021 | Study was not a peer-reviewed empirical paper |
| Snyder-Roche et al., 2011 | Study did not assess relevant outcomes |
| Sotsky & Worthington, 2021 | Training focused on addressing microaggressions toward peers |
| Sotto-Santiago et al., 2020 | Training focused on addressing microaggressions toward peers |
| Sowers, 2019 | Study did not assess relevant outcomes |
| Spieß et al., 2006 | No English version available |
| Steed, 2010 | Duplicate identified at this stage of screening |
| Stein et al., 2022 | Conference abstract only (no full text) |
| Steinfeldt, J. A., & Steinfeldt, 2012 | Study was not a peer-reviewed empirical paper |
| Steinfeldt & Wong, 2010 | Duplicate identified at this stage of screening |
| Stephenson et al., 2021 | Training not focused on implicit bias |
| Stevenson, 2018 | Training not focused on implicit bias |
| Steward et al., 2020 | Training not focused on implicit bias |
| Stewart & Whiteman, 2013 | Study was not a peer-reviewed empirical paper |
| Stewart et al., 2022 | Training not focused on healthcare providers |
| Stone & Moskowitz, 2011 | Study was not a peer-reviewed empirical paper |
| Strewler et al., 2021 | Conference abstract only (no full text) |
| Strohbehn et al., 2020 | Training not focused on implicit bias |
| Stuart et al., 2014 | Training not focused on implicit bias |
| Sun et al., 2013 | Training not focused on implicit bias |
| Swiderski et al., 2011 | Conference abstract only (no full text) |
| Symons et al., 2014 | Training not focused on implicit bias |
| Tajeu et al., 2021 | Conference abstract only (no full text) |
| Tate & Prestidge, 2020 | Conference abstract only (no full text) |
| Taylor et al., 2022 | Conference abstract only (no full text) |
| Tedesco & Albino, 2011 | Study was not a peer-reviewed empirical paper |
| Tenorio da Silva et al., 2020 | Conference abstract only (no full text) |
| Thackrah & Thompson, 2013 | Training not focused on implicit bias |
| Toporek & Pope-Davis, 2005 | Training not focused on implicit bias |
| Torino et al., 2011 | Study was not a peer-reviewed empirical paper |
| Tsai & Michelson, 2017 | Duplicate identified at this stage of screening |
| Tsai & Michelson, 2020 | Training not focused on implicit bias |
| Tuan & Hsia, 2021 | Conference abstract only (no full text) |
| Tucker et al., 2016 | Training not focused on implicit bias |
| Uys et al., 2009 | Training not focused on implicit bias |
| Vaimberg et al., 2021 | Duplicate identified at this stage of screening |
| Van Bewer et al., 2021 | Training not focused on implicit bias |
| van Ryn et al., 2015 | Training not focused on implicit bias |
| Van Winkle et al., 2021 | Training not focused on healthcare providers |
| Varas-Diaz et al., 2016 | Training not focused on implicit bias |
| Varkey et al., 2006 | Training not focused on implicit bias |
| Victoroff et al., 2013 | Training not focused on implicit bias |
| Villani & Kovess-Masfety, 2017 | Training not focused on implicit bias |
| Villegas et al., 2019 | Conference abstract only (no full text) |
| Volkman, 2007 | Conference abstract only (no full text) |
| Wain et al., 2016 | Study was not a peer-reviewed empirical paper |
| Waite, & Calamaro, 2010 | Study was not a peer-reviewed empirical paper |
| Wallace et al., 2006 | Training not focused on implicit bias |
| Wardhere et al., 2022 | Conference abstract only (no full text) |
| Wear et al., 2017 | Study was not a peer-reviewed empirical paper |
| Webb & Sergison, 2003 | Training not focused on implicit bias |
| Webster, 2009 | Training not focused on implicit bias |
| Wechsler et al., 2020 | Training not focused on implicit bias |
| Weech-Maldonado et al., 2018 | Study did not assess relevant outcomes |
| Werkhoven, 2021 | Training not focused on implicit bias |
| Werkhoven et al., 2022 | Training not focused on implicit bias |
| Wheeler et al., 2018 | Training not focused on implicit bias |
| Williams, 2016 | Study was not a peer-reviewed empirical paper |
| Wolsiefer & Stone, 2019 | Study was not a peer-reviewed empirical paper |
| Woods et al., 2015 | Conference abstract only (no full text) |
| Wray et al., 2008 | Training not focused on implicit bias |
| Wu et al., 2008 | Training not focused on implicit bias |
| Yiu et al., 2010 | Training not focused on implicit bias |
| Yotis et al., 2017 | Training not focused on implicit bias |
| Yousufzai et al., 2022 | Conference abstract only (no full text) |
| Zaske et al., 2014 | Conference abstract only (no full text) |
| Zeidan et al., 2018 | Study was not a peer-reviewed empirical paper |
| Zhou et al., 2022 | Study was not a peer-reviewed empirical paper |

**References for Table S2**

Aboshaiqah, A. E., Tumala, R. B., Inocian, E. P., Almutairi, A. F., & Atallah, M. (2017). Enhancing culturally competent nursing care in Saudi Arabia. *Journal of Cultural Diversity*, *24*(1), 20-26.

Abou Baker, N., Anderson, D., & Brooks, B. (2021). Addressing Sickle Cell Disease Implicit Bias in Internal Medicine Residents. *Blood*, *138*, 2965. <https://doi.org/doi:10.1182/blood-2021-151733>

Abraham, O., & Torner, C. (2021). Preparing graduate students for community engagement in health services research. *Innovations in pharmacy*, *12*(2). <https://doi.org/doi:10.24926/iip.v12i2.3469>

Acholonu, R. G., Cook, T. E., Roswell, R. O., & Greene, R. E. (2020). Interrupting microaggressions in health care settings: A guide for teaching medical students. *MedEdPORTAL: the journal of teaching and learning resources*, *16*, 10969. <https://doi.org/doi:10.15766/mep_2374-8265.10969>

Adames H. Y., Fuentes M. A. (2011). Having a voice and being heard: Exploring the impact of multiple social identities on the self. In Pope M., Pangelinan J., Coker A. (Eds.), *Experiential activities for teaching multicultural competency in counseling* (pp. 49–51). Alexandria, VA: American Counseling Association Press.

Adams, M. A. (2021). Don't fix the women, fix the system: recognizing and addressing implicit gender bias in gastroenterology training and practice. *Gastrointestinal Endoscopy*, *93*(5), 1057-1059. <https://doi.org/doi:10.1016/j.gie.2020.10.022>

Adams, V., & Kaufman, S. R. (2011). Ethnography and the making of modern health professionals. *Culture, Medicine and Psychiatry*, *35*(2), 313-320. <https://doi.org/doi:10.1007/s11013-011-9216-0>

Adelman, R. D., Capello, C. F., LoFaso, V., Greene, M. G., Konopasek, L., & Marzuk, P. M. (2007). Introduction to the older patient: a 'first exposure' to geriatrics for medical students. *Journal of the American Geriatrics Society*, *55*(9), 1445-1450. <https://doi.org/doi:10.1111/j.1532-5415.2007.01301.x>

Agboola, B. (2021). We need to stop failing our patients. *Southern Medical Journal*, *114*(6), 350. <https://doi.org/doi:10.14423/SMJ.0000000000001253>

Al-Mateen, C. S. (2017). Unconscious bias, microaggressions, and health. *Journal of the American Academy of Child and Adolescent Psychiatry*, *56*(10), S76. <https://doi.org/doi:10.1016/j.jaac.2017.07.300>

Ali, M. A., & Halim, M. U. (2014). Recent ideas on ethnic diversity in medical education: one step forward, two steps back? *Medical Education*, *48*(4), 451. <https://doi.org/doi:10.1111/medu.12383>

Allen, J., Brown, L., Duff, C., Nesbitt, P., & Hepner, A. (2013). Development and evaluation of a teaching and learning approach in cross-cultural care and antidiscrimination in university nursing students. *Nurse Education Today*, *33*(12), 1592-1598. <https://doi.org/doi:10.1016/j.nedt.2012.12.006>

Alspach, J. G. (2018). Implicit bias in patient care: An endemic blight on quality care. *Critical Care Nurse*, *38*(4), 12-16. <https://doi.org/doi:10.4037/ccn2018698>

Andrews, J., & Hay, S. F. (2018). Updates on the ambulatory half-day: Five curricular innovations. *Journal of General Internal Medicine*, *33*(2), 750-751.

Ang, W., Verpooten, L., De Winter, B., & Bombeke, K. (2020). Stereotype rebound: From cultural competence to diversity awareness. *Medical Education*, *54*(5), 468-469. <https://doi.org/doi:10.1111/medu.14110>

Anonymous. (2008). Raising PCT staff awareness on equality and diversity. *Community Practitioner: The Journal of the Community Practitioners' & Health Visitors' Association*, *81*(1), 12-15.

Ansari, A., Barnett, M., Elk, R., Chen, H., Harris, H., Macip-Rodriguez, P., Thomson, R., & Williams, S. (2020). Improving the Care of Diverse Patient Populations: Let's Advance the Dialogue for Strategies to Address and Navigate the Elephant in the Room (P15). *Journal of Pain and Symptom Management*, *59*(2), 399-400. <https://doi.org/10.1016/j.jpainsymman.2019.12.026>

Ard, C. K., Brown, M. E., Whybrow, P., Adams, J., Finn, G., & O'Regan, A. (2021). Professional identity development within longitudinal integrated clerkships: A qualitative, multisite, international study. *Journal of General Internal Medicine*, *36*, S468.

Arif, S. A., & Schlotfeldt, J. (2021). Gaps in Measuring and Mitigating Implicit Bias in Healthcare. *Frontiers in Pharmacology*, *12,* 633565. <https://doi.org/doi:10.3389/fphar.2021.633565>

Ascoli, M., Palinski, A., Owiti, J. A., De Jongh, B., & Bhui, K. S. (2012). The culture of care within psychiatric services: tackling inequalities and improving clinical and organisational capabilities. *Philosophy, ethics, and humanities in medicine*, *7*, 12. <https://doi.org/doi:10.1186/1747-5341-7-12>

Ashford, R. D., Brown, A. M., & Curtis, B. (2018). Substance use, recovery, and linguistics: The impact of word choice on explicit and implicit bias. *Drug and Alcohol Dependence*, *189*, 131-138. <https://doi.org/doi:10.1016/j.drugalcdep.2018.05.005>

Ashurst, A. (2019). Equality and diversity training for care staff. *Nursing & Residential Care*, *21*(1), 54-56.

Astudillo, Y. M., & Noulas, C. (2022). Foundation and Decision-Making Framework for Understanding and Combating Implicit Bias in Pediatrics. *Journal of Investigative Medicine*, *70*, 1168-1169.

Avant, N. D., & Gillespie, G. L. (2019). Pushing for health equity through structural competency and implicit bias education: A qualitative evaluation of a racial/ethnic health disparities elective course for pharmacy learners. *Currents in Pharmacy Teaching and Learning*, *11*(4), 382-393. <https://doi.org/doi:10.1016/j.cptl.2019.01.013>

Avant, N. D., Weed, E., Connelly, C., Hincapie, A. L., & Penm, J. (2018). Qualitative Analysis of Student Pharmacists’ Reflections of Harvard's Race Implicit Association Test. *Currents in Pharmacy Teaching and Learning*, *10*(5), 611-617. <https://doi.org/doi:10.1016/j.cptl.2018.02.002>

Bahrke, B., De Oliveira, K., Scheel, M. H., Beck, B., & Hopp, J. (2014). Longitudinal integration of cultural components into a physician assistant program's clinical year may improve cultural competency. *The Journal of Physician Assistant Education: The Official Journal of the Physician Assistant Education Association*, *25*(1), 33-37. <https://doi.org/doi:10.1097/01367895-201425010-00005>

Baig, A. A., Benitez, A., Gao, Y., Quinn, M. T., Paredes, A. Z., Monnot, L., & Brazda, K. (2014). Local patients, local stories: A Latino cultural competency training program for healthcare providers. *Journal of General Internal Medicine*, *29*, S140-S141.

Bakke, K., Sidhar, K., & Kumagai, A. K. (2014). Exploring matters of race through dialogue in the University of Michigan Medical School's longitudinal case studies program. *The Virtual Mentor*, *16*(6), 442-449. <https://doi.org/doi:10.1001/virtualmentor.2014.16.06.medu1-1406>

Ball, A., Cheetham, A., ra, & Freeman, B. (2021). Addressing health equity and bias training in resident education. *Pediatrics*, *147*(3), 642-643. <https://doi.org/doi:10.1542/peds.147.3MA7.643a>

Bansal, A. (2016). Turning cross-cultural medical education on its head: Learning about ourselves and developing respectful curiosity. *Family Medicine and Community Health*, *4*(2), 41-44. <https://doi.org/doi:10.15212/FMCH.2016.0109>

Basham, K. (2004). Weaving a tapestry: Anti-racism and the pedagogy of clinical social work practice. *Smith College Studies in Social Work*, *74*(2), 389-314. <https://doi.org/doi:10.1080/00377310409517717>

Bayar, M. R., Poyraz, B. C., Aksoy-Poyraz, C., & Arikan, M. K. (2009). Reducing mental illness stigma in mental health professionals using a web-based approach. *The Israel Journal of Psychiatry and Related Sciences*, *46*(3), 226-230.

Beam, M. A., Muller, B., Lee, J., & Gorman, P. (2016). Exploring the structural causes of health disparities: Novel, student-led structural competency and leadership curriculum at Oregon Health & Sciences University. *Journal of General Internal Medicine*, *31*(2), S820.

Bellack, J. P. (2015). Unconscious Bias: An Obstacle to Cultural Competence. *The Journal of nursing education*, *54*(9), S63-64. <https://doi.org/doi:10.3928/01484834-20150814-12>

Bello, R., Dougherty, M., Hochstetler, V., Prigge, R., Ausejo, D., & Ehlers, E. (2022). Equity and inclusion equals diversity. Association of Schools Advancing Health Professions (ASAHP) Annual Conference October 20-21, 2021, The Westin Long Beach, Long Beach, California. *Journal of Allied Health*, *51*(1), 78-78.

Bennett, C., Hamilton, E. K., & Rochani, H. (2019). Exploring race in nursing: Teaching nursing students about racial inequality using the historical lens. *Online Journal of Issues in Nursing*, *24*(2). <https://doi.org/doi:10.3912/OJIN.Vol24No02PPT20>

Bennett, J., Kalathil, J., & Keating, F. (2007). One size doesn't fit all. *Mental Health Today (Brighton, England)*, 28-31.

Benoit, L. J., Travis, C., Swan Sein, A., Quiah, S. C., Amiel, J., & Gowda, D. (2020). Toward a bias-free and inclusive medical curriculum: Development and implementation of student-initiated guidelines and monitoring mechanisms at one institution. *Academic Medicine: Journal of the Association of American Medical Colleges*, *95*(12), S145-S149. <https://doi.org/doi:10.1097/ACM.0000000000003701>

Bey, A., Garrison, E., Li, A. N., Mosley, D., & Nettles, A. L. (2022). Multidisciplinary education: piloting an unconscious bias-maternal mortality curriculum in obstetrics. *Obstetrics and Gynecology*, *139*, 19S. <https://doi.org/doi:10.1097/01.AOG.0000826580.44006.c9>

Bheenuck, S., Miers, M., Pollard, K., & Young, P. (2007). Race equality education: implications of an audit of student learning. *Nurse Education Today*, *27*(5), 396-405. <https://doi.org/doi:10.1016/j.nedt.2006.06.003>

Birtel, M. D., & Crisp, R. J. (2012). 'Treating' prejudice: An exposure-therapy approach to reducing negative reactions toward stigmatized groups. *Psychological Science*, *23*(11), 1379-1386. <https://doi.org/doi:10.1177/0956797612443838>

Borowsky, H., Morinis, L., & Garg, M. Disability and ableism in medicine: A curriculum for medical students. *MedEdPORTAL*, *17*, 11073. <https://doi.org/doi:10.15766/mep_2374-8265.11073>

Boscardin, C. K. (2015). Reducing implicit bias through curricular interventions. *Journal of General Internal Medicine*, *30*(12), 1726-1728. <https://doi.org/doi:10.1007/s11606-015-3496-y>

Bowman, S. H., Moreno-Walton, L., Ezenkwele, U. A., & Heron, S. L. (2011). Diversity in emergency medicine education: Expanding the horizon. *Academic Emergency Medicine*, *18*(10), S104-S109. <https://doi.org/doi:10.1111/j.1553-2712.2011.01184.x>

Boykins, Branson L. (2016). The influence of feedback on implicit Bias in a sample of primarily Caucasian women counselors-in-training. *Dissertations*, *2491*. Retrieved from

<https://scholarworks.wmich.edu/dissertations/2491>

Brammer, S. V. C. N. E., Regan, S. L., Collins, C. M. B. A., & Gillespie, G. L. D. N. P. (2022). Developing innovative virtual reality simulations to increase health care providers' understanding of social determinants of health. *Journal of Continuing Education in the Health Professions*, *42*(1), 60-65. <https://doi.org/doi:10.1097/CEH.0000000000000400>

Braun, H. M., Garcia-Grossman, I. R., Quinones-Rivera, A., & Deutsch, M. B. (2017). Outcome and Impact Evaluation of a Transgender Health Course for Health Profession Students. *LGBT health*, *4*(1), 55-61. <https://doi.org/doi:10.1089/lgbt.2016.0119>

Bridgeman-Bunyoli, A., Mitchell, S. R., Bin Abdullah, A. H. M., Schwoeffermann, T., Phoenix, T., Goughnour, C., Hines-Norwood, R., & Wiggins, N. (2015). "It's in my veins": Exploring the role of an Afrocentric, popular education-based training program in the empowerment of African American and African community health workers in Oregon. *The Journal of Ambulatory Care Management*, *38*(4), 297-308. <https://doi.org/doi:10.1097/JAC.0000000000000112>

Bristol, S., Kostelec, T., & MacDonald, R. (2018). Improving emergency health care workers’ knowledge, competency, and attitudes toward lesbian, gay, bisexual, and transgender patients through interdisciplinary cultural competency training. *Journal of Emergency Nursing*, *44*(6), 632-639. <https://doi.org/doi:10.1016/j.jen.2018.03.013>

Brondani, M. A., & Paterson, R. (2011). Teaching lesbian, gay, bisexual, and transgender issues in dental education: a multipurpose method. *Journal of Dental Education*, *75*(10), 1354-1361.

Brooks, K. C., Rougas, S., & George, P. When race matters on the wards: Talking about racial health disparities and racism in the clinical setting. *MedEdPORTAL*, *12*, 10523. <https://doi.org/doi:10.15766/mep_2374-8265.10523>

Broomes Williamson, L. (2020). 46.3 Fostering lifelong learning regarding implicit racial bias in child psychiatry. *Journal of the American Academy of Child and Adolescent Psychiatry*, *59*(10), S71. <https://doi.org/doi:10.1016/j.jaac.2020.07.298>

Brown, K. M., & Bright, L. M. (2017). Teaching caring and competence: Student transformation during an older adult focused service-learning course. *Nurse Education in Practice*, *27*, 29-35. <https://doi.org/doi:10.1016/j.nepr.2017.08.013>

Browne, A. J., Varcoe, C., Ford-Gilboe, M., Nadine Wathen, C., Smye, V., Jackson, B. E., Wallace, B., Pauly, B. B., Herbert, C. P., Lavoie, J. G., Wong, S. T., & Blanchet Garneau, A. (2018). Disruption as opportunity: Impacts of an organizational health equity intervention in primary care clinics. *International Journal for Equity in Health*, *17*(1), 154. <https://doi.org/doi:10.1186/s12939-018-0820-2>

Buchs, S., & Mulitalo, K. (2016). Implicit bias: An opportunity for physician assistants to mindfully reduce health care disparities. *Journal of Physician Assistant Education*, *27*(4), 193-195. <https://doi.org/doi:10.1097/JPA.0000000000000098>

Burbank, P. M., Dowling-Castronovo, A., Crowther, M. R., & Capezuti, E. A. (2006). Improving knowledge and attitudes toward older adults through innovative educational strategies. *Journal of Professional Nursing: Official Journal of the American Association of Colleges of Nursing*, *22*(2), 91-97. <https://doi.org/doi:10.1016/j.profnurs.2006.01.007>

Burgess, D. J., Beach, M. C., & Saha, S. (2017). Mindfulness practice: A promising approach to reducing the effects of clinician implicit bias on patients. *Patient Education and Counseling*, *100*(2), 372-376. <https://doi.org/doi:10.1016/j.pec.2016.09.005>

Burns, K., & McNally, G. A. (2021). Implicit bias training improving outcomes for patients with cancer who have substance use disorders. *Clinical Journal of Oncology Nursing*, *25*(5), 595-599. <https://doi.org/doi:10.1188/21.CJON.595-599>

Butler-Byrd, N., Nieto, J., & Senour, M. N. (2006). Working successfully with diverse students and communities: The community-based block counselor preparation program. *Urban Education*, *41*(4), 376-401. <https://doi.org/doi:10.1177/0042085906289706>

Caffrey, L., Wyatt, D., Fudge, N., Mattingley, H., Williamson, C., & McKevitt, C. (2016). Gender equity programmes in academic medicine: a realist evaluation approach to Athena SWAN processes. *BMJ Open*, *6*(9), e012090. <https://doi.org/doi:10.1136/bmjopen-2016-012090>

Cahn, P. S. (2017). Recognizing and reckoning with unconscious bias: A workshop for health professions faculty search committees. *MedEdPORTAL: The Journal of Teaching and Learning Resources*, *13*, 10544. <https://doi.org/doi:10.15766/mep_2374-8265.10544>

Calardo, S. J., Kou, M., Port, C., McKnight, N., Switzer, B. E., Halmon, K., Lobo, K., Benghanem, G., & Seo-Mayer, P. W. (2022). Realizing inclusion and systemic equity in medicine: Upstanding in the medical workplace (RISE UP)- An antibias curriculum. *MedEdPORTAL: The Journal of Teaching and Learning Resources*, *18*, 11233. <https://doi.org/doi:10.15766/mep_2374-8265.11233>

Carbonneau, M. K. (2021). Examining the experience of white privilege for human service providers using the expressive therapies (Order No. 28494028). Available from ProQuest Dissertations & Theses Global. Retrieved from <http://proxy.library.vcu.edu/login?url=https://www.proquest.com/dissertations-theses/examining-experience-white-privilege-human/docview/2531161640/se-2>

Carey, J., Mackey, C., & Tran, M. (2022). Raising bias awareness in students. *Western Journal of Emergency Medicine*, *23*, S29-S30.

Carnes, M., Sheridan, J., Fine, E., Lee, Y.-G., Filut, A., & Topp, S. (2021). Engaging faculty in a workshop intervention on overcoming the influence of implicit bias. *Journal of Clinical and Translational Science*, *5*(1), e135. <https://doi.org/doi:10.1017/cts.2021.796>

Cartz Piver, L., Maillet, A., Leperre Desplanques, A., Krolak Salmon, P., Calvet, B., Dumoitier, N., Raychevae, M., & Rejdak, K. (2020). Anti-stigma training reduces stereotypes and increases GPs confidence in managing Neurocognitive disorders. *European Journal of Neurology*, *27*, 137.

Castillo, L. G., Brossart, D. F., Reyes, C. J., Conoley, C. W., & Phoummarath, M. J. (2007). The influence of multicultural training on perceived multicultural counseling competencies and implicit racial prejudice. *Journal of Multicultural Counseling and Development*, *35*(4), 243-255. <https://doi.org/doi:10.1002/j.2161-1912.2007.tb00064.x>

Centola, D., Guilbeault, D., Sarkar, U., Khoong, E., & Zhang, J. (2021). The reduction of race and gender bias in clinical treatment recommendations using clinician peer networks in an experimental setting. *Nature Communications*, *12*(1), 6585. <https://doi.org/doi:10.1038/s41467-021-26905-5>

Chang, H.-P., & Lee, Y.-J. (2018). LGBT issues and counseling competency: The impact of training on enhancing knowledge, awareness and skills. *Chinese Journal of Guidance and Counseling*, *52*, 51-80.

Chao, R. C. L. (2012). Racial/ethnic identity, gender‐role attitudes, and multicultural counseling competence: The role of multicultural counseling training. *Journal of Counseling & Development*, *90*(1), 35-44. <https://doi.org/doi:10.1111/j.1556-6676.2012.00006.x>

Chao, R. C. L. (2013). Race/ethnicity and multicultural competence among school counselors: Multicultural training, racial/ethnic identity, and color-blind racial attitudes. *Journal of Counseling and Development*, *91*(2), 140-151. <https://doi.org/doi:10.1002/j.1556-6676.2013.00082.x>

Chao, R. C. L., Wei, M., Good, G. E., & Flores, L. Y. (2011). Race/ethnicity, color-blind racial attitudes, and multicultural counseling competence: The moderating effects of multicultural counseling training. *Journal of Counseling Psychology*, *58*(1), 72-82. <https://doi.org/doi:10.1037/a0022091>

Charles, L. L., Thomas, D., & Thornton, M. L. (2005). Overcoming bias toward same-sex couples: a case study from inside an MFT ethics classroom. *Journal of Marital and Family Therapy*, *31*(3), 239-249. <https://doi.org/doi:10.1111/j.1752-0606.2005.tb01566.x>

Chary, A. N., Molina, M. F., Dadabhoy, F. Z., Manch, & a, E. C. (2020). Addressing racism in medicine through a resident-led health equity retreat. *The Western Journal of Emergency Medicine*, *22*(1), 41-44. <https://doi.org/doi:10.5811/westjem.2020.10.48697>

Chew, K. S., Durning, S. J., & van Merrienboer, J. J. (2016). Teaching metacognition in clinical decision-making using a novel mnemonic checklist: an exploratory study. *Singapore Medical Journal*, *57*(12), 694-700. <https://doi.org/doi:10.11622/smedj.2016015>

Chow, C. J., Case, G. A., & Matias, C. E. Tools for discussing identity and privilege among medical students, trainees, and faculty. *MedEdPORTAL*, *15*, 10864. <https://doi.org/doi:10.15766/mep_2374-8265.10864>

Chrobot-Mason, D. (2012). Developing multicultural competence to improve cross-race work relationships. *The Psychologist-Manager Journal*, *15*(4), 199-218. [https://doi.org/doi:https://doi.org/10.1080/10887156.2012.730440](https://doi.org/doi:https:/doi.org/10.1080/10887156.2012.730440)

Clarke, M. J., Laughlin-Tommaso, S., & Seegmiller Renner, A. (2021). Teaching about health disparities: pedagogy, curriculum, and learning theory. *American Journal of Bioethics*, *21*(9), 18-20. <https://doi.org/doi:10.1080/15265161.2021.1952340>

Cleeves, M., Tomcho, M., & Adams, J. (2019). Stretching beyond clerkships: A longitudinal post-clerkship course in social medicine. *Journal of General Internal Medicine*, *34*(2), S847.

Clement, S., van Nieuwenhuizen, A., Kassam, A., Flach, C., Lazarus, A., de Castro, M., McCrone, P., Norman, I., & Thornicroft, G. (2012). Filmed v. live social contact interventions to reduce stigma: randomised controlled trial. *The British Journal of Psychiatry: The Journal of Mental Science*, *201*(1), 57-64. <https://doi.org/doi:10.1192/bjp.bp.111.093120>

Clendon, J. (2020). Talking about unconscious bias: It is possible to challenge our own biases and assumptions, and train ourselves to focus on the individual in front of us. *Kai Tiaki Nursing New Zealand*, *26*(10), 32-33.

Closson, R. B., & Rhodes, C. M. (2011). Reflections on a positively deviant course on race and racism. *Reflective Practice*, *12*(4), 457-468. <https://doi.org/doi:10.1080/14623943.2011.590335>

Sarabia-Cobo, C. M., & Pfeiffer, C. C. (2015). Changing negative stereotypes in old age in nursing students. *Gerokomos*, *26*(1), 10-12. <https://doi.org/doi:0.1016/j.nedt.2015.06.006>

Cooper, M. B., Chacko, M., & Christner, J. (2018). Incorporating LGBT health in an undergraduate medical education curriculum through the construct of social determinants of health. *MedEdPORTAL*, *14*, 10781. <https://doi.org/doi:10.15766/mep_2374-8265.10781>

Costa, A. B., elli, Pase, P. F., de Camargo, E. S., Guaranha, C., Caetano, A. H., Kveller, D., da Rosa Filho, H. T., Catelan, R. F., Koller, S. H., & Nardi, H. C. (2016). Effectiveness of a multidimensional web-based intervention program to change Brazilian health practitioners' attitudes toward the lesbian, gay, bisexual and transgender population. *Journal of Health Psychology*, *21*(3), 356-368. <https://doi.org/doi:10.1177/1359105316628748>

Crabtree, J., & Mack, J. (2010). Designing a training programme to improve staff attitudes towards people with dementia. *Nursing Times*, *106*(39), 14-16.

Crampton, P., Dowell, A., Parkin, C., & Thompson, C. (2003). Combating effects of racism through a cultural immersion medical education program. *Academic Medicine: Journal of the Association of American Medical Colleges*, *78*(6), 595-598. <https://doi.org/doi:10.1097/00001888-200306000-00008>

Cravens, J. D., Pratt, K. J., Palmer, E., & Aamar, R. (2016). Marriage and family therapy students’ views on including weight bias training into their clinical programs. *Contemporary Family Therapy: An International Journal*, *38*(2), 210-222. <https://doi.org/doi:10.1007/s10591-015-9366-2>

Crawford, C. (2020). The Everyone Project unveils implicit bias training guide. *Annals of Family Medicine*, *18*(2), 182-183. <https://doi.org/doi:10.1370/afm.2525>

Cropper-Williams, D. (2018). Culturally appropriate training to build better relationships between men who have sex with men (MSM) of color and their health providers. *Sexually Transmitted Diseases*, *45*, S109.

Crowe, R., Gillespie, C., Girmay, B., Cook, T., Ravenell, J., & Greene, R. E. (2021). Foundations in equity: A novel, virtual, intensive, foundational curriculum on health equity for entering first-year medical students. *Journal of General Internal Medicine*, *36*, S64.

Cucco, E. (2020). Who's afraid of the big bad unconscious: Working with countertransference in training. *Journal of Psychotherapy Integration*, *30*(1), 52-59. <https://doi.org/doi:10.1037/int0000163>

Cutler, J. L., Harding, K. J., Hutner, L. A., Cortl, , C., & Graham, M. J. (2012). Reducing medical students' stigmatization of people with chronic mental illness: a field intervention at the "living museum" state hospital art studio. *Academic Psychiatry: The Journal of the American Association of Directors of Psychiatric Residency Training and the Association for Academic Psychiatry*, *36*(3), 191-196. <https://doi.org/doi:10.1176/appi.ap.10050081>

Daniel, M., Carney, M., Khandelwal, S., Merritt, C., Cole, M., Malone, M., Hemphill, R. R., Peterson, W., Burkhardt, J., Hopson, L., & Santen, S. A. (2017). Cognitive debiasing strategies: A faculty development workshop for clinical teachers in emergency medicine. *MedEdPORTAL*, *13*, 10646. <https://doi.org/doi:10.15766/mep_2374-8265.10646>

Davtyan, M., Bartell, S. M., & Lakon, C. M. (2020). Assessing the efficacy of a PhotoVoice-informed HIV stigma training for health care workers. *AIDS and Behavior*, *24*(1), 65-80. <https://doi.org/doi:10.1007/s10461-019-02710-6>

Debiasi, L. B., & Selleck, C. S. (2017). Cultural competence training for primary care nurse practitioners: An intervention to increase culturally competent care. *Journal of Cultural Diversity*, *24*(2), 39-45. Retrieved from <http://proxy.library.vcu.edu/login?url=https://www.proquest.com/scholarly-journals/cultural-competence-training-primary-care-nurse/docview/1974490916/se-2>

DeRicco, J. N., & Sciarra, D. T. (2005). The immersion experience in multicultural counselor training: Confronting covert racism. *Journal of Multicultural Counseling and Development*, *33*(1), 2-16. <https://doi.org/doi:10.1002/j.2161-1912.2005.tb00001.x>

Desai, M. U., Paranamana, N., Restrepo-Toro, M., O'Connell, M., Davidson, L., & Stanhope, V. (2021). Implicit organizational bias: Mental health treatment culture and norms as barriers to engaging with diversity. *American Psychologist*, *76*(1), 78-90. <https://doi.org/doi:10.1037/amp0000621>

Dhanani, Z., Huynh, N., Tan, L., Kottakota, H., Lee, R., & Poullos, P. (2022). Deconstructing ableism in health care settings through case-based learning. *MedEdPORTAL*, *18*, 11253. <https://doi.org/doi:10.15766/mep_2374-8265.11253>

Diachun, L., Van Bussel, L., Hansen, K. T., Charise, A., & Rieder, M. J. (2010). "But I see old people everywhere": dispelling the myth that eldercare is learned in nongeriatric clerkships. *Academic Medicine: Journal of the Association of American Medical Colleges*, *85*(7), 1221-1228. <https://doi.org/doi:10.1097/ACM.0b013e3181e0054f>

Diaz Del Carpio, R. O., Lema, P. C., Dubocovich, M. L., Makdissi, R., & Burke, B. A. (2018). Cultural and structural competency training for medical residents. *Journal of General Internal Medicine*, *33*(2), 696-697.

DiBrito, S. R., Lopez, C. M., Jones, C., & Mathur, A. (2019). Reducing implicit bias: Association of Women Surgeons #HeForShe task force best practice recommendations. *Journal of the American College of Surgeons*, *228*(3), 303-309. <https://doi.org/doi:10.1016/j.jamcollsurg.2018.12.011>

Dickson, G., Argus-Calvo, B., & Tafoya, N. (2010). Multicultural counselor training experiences: Training effects and perceptions of training among a sample of predominately Hispanic students. *Counselor Education and Supervision*, *49*(4), 247-265. <https://doi.org/doi:10.1002/j.1556-6978.2010.tb00101.x>

Diedrichs, P. C., & Barlow, F. K. (2011). How to lose weight bias fast! Evaluating a brief anti‐weight bias intervention. *British Journal of Health Psychology*, *16*(4), 846-861. <https://doi.org/doi:10.1111/j.2044-8287.2011.02022.x>

Dion, K. (2019). Teaching nursing students how to decrease the stigma against people who use drugs. *Nurse Educator*, *44*(6), 335-337. <https://doi.org/doi:10.1097/NNE.0000000000000647>

Doucette, H. J., Maillet, P. J., Brillant, M. G., & Tax, C. L. (2015). Dental hygiene students' perceptions of a cultural competence component in a tobacco dependence education curriculum: a pilot study. *Journal of dental education*, *79*(6), 680-685.

Doyle, K. E., & Cruickshank, M. (2012). Stereotyping stigma: Undergraduate health students' perceptions at handover. *Journal of Nursing Education*, *51*(5), 255-261. <https://doi.org/doi:10.3928/01484834-20120309-03>

Dupre, A.-M., & Goodgold, S. (2007). Development of physical therapy student cultural competency through international community service. *Journal of Cultural Diversity*, *14*(3), 126-134.

Eberly, L. A., Julien, H., & Adusumalli, S. (2021). Weaving antiracism practice and equity into the fabric of cardiovascular fellowship: A new training paradigm. *Journal of the American College of Cardiology*, *78*(23), 2382-2386. <https://doi.org/doi:10.1016/j.jacc.2021.07.063>

Echeverri, M., & Dise, T. (2017). Racial dynamics and cultural competence training in medical and pharmacy education. *Journal of Health Care for the Poor & Underserved*, *28*(1), 279-302. <https://doi.org/doi:10.1353/hpu.2017.0023>

Edgoose, J. Y. C., Speights, J. S. B., White-Davis, T., Guh, J., Bullock, K., Roberson, K., De Leon, J., Ferguson, W., & Saba, G. W. (2021). Teaching about racism in medical education: A mixed-method analysis of a train-the-trainer faculty development workshop. *Family Medicine*, *53*(1), 23-31. <https://doi.org/doi:10.22454/FamMed.2021.408300>

Edwards, C., Feldman, M., McKay, S. W., Lemay, M., & Kang, L. (2021). Impacting implicit bias through the arts and humanities. *Journal of General Internal Medicine*, *36*, S443.

Ellis, J., Darling, A., Grossestreuer, A. V., ry, A. M., Dubosh, N. M., & Ullman, E. (2021). Racial disparities in emergency medicine: A novel curriculum. *Academic Emergency Medicine*, *28*, S410-S411.

Ertl, M. M., Agiliga, A. U., Martin, C. M., Taylor, E. J., Kirkinis, K., Friedler, M. L., Kimber, J. M., McNamara, M. L., Pazienza, R. L., Cabrera Tineo, Y. A., & Eklund, A. C. (2020). "Hands-on" learning in a health service psychology doctoral program through social justice consultation. *Training and Education in Professional Psychology, 15*(3), 230–241. <https://doi.org/doi:10.1037/tep0000311>

Etengoff, C. (2020). Repositioning cultural competency with clinical doctoral students: Unpacking intersectionality, standpoint theory, and multiple minority stress/resilience. *Women and Therapy*, *43*(3), 348-364. <https://doi.org/doi:10.1080/02703149.2020.1729472>

Evans, E. V., & McCaughan, R. P. (2020). Comment on “the potential effect of the psychiatric clerkship and contact-based hypothesis on explicit and implicit stigmatizing attitudes of Canadian medical students towards mental illness”. *Academic Psychiatry*, *44*(2), 242-243. <https://doi.org/doi:10.1007/s40596-019-01159-y>

Farmer, D. W., Knebl, J. A., & Pitts, Y. (2011). Encore presentation SAGE an innovative competency-based senior mentoring program. *Journal of the American Geriatrics Society*, *59*, S181.

Fisher, A. K., Moore, D. J., Simmons, C., & Allen, S. C. (2017). Teaching social workers about microaggressions to enhance understanding of subtle racism. *Journal of Human Behavior in the Social Environment*, *27*(4), 346-355. <https://doi.org/doi:10.1080/10911359.2017.1289877>

Fisher, H., Chatterjee, P., Shapiro, J., Katz, J. T., & Yialamas, M. (2020). Microaggression response training workshop for internal medicine residents. *Journal of General Internal Medicine*, *35*, S751.

Fisher, H. N., Chatterjee, P., Shapiro, J., Katz, J. T., & Yialamas, M. A. (2021). “Let’s talk about what just happened”: A single-site survey study of a microaggression response workshop for internal medicine residents. *Journal of General Internal Medicine, 36,* 3592-3594. <https://doi.org/doi:10.1007/s11606-020-06576-6>

Fitzgerald, S. (2021). Neurology training steps up to focus on racism and social injustice. *Neurology Today*, *21*(11), 12-14. Retrieved from <https://journals.lww.com/neurotodayonline/fulltext/2021/06030/neurology_training_steps_up_to_focus_on_racism_and.5.aspx>

Fitzgerald, S. N., Leslie, K. F., Simpson, R., Jones, V. F., & Barnes, E. T. (2018). Culturally effective care for refugee populations: Interprofessional, interactive case studies. *MedEdPORTAL*, *14*, 10668. <https://doi.org/doi:10.15766/mep_2374-8265.10668>

Flanagan, E. H., Buck, T., Gamble, A., Hunter, C., Sewell, I., & Davidson, L. (2016). "Recovery speaks": A Photovoice intervention to reduce stigma among primary care providers. *Psychiatric services (Washington, D.C.)*, *67*(5), 566-569. <https://doi.org/doi:10.1176/appi.ps.201500049>

Flatt-Fultz, E., & Phillips, L. A. (2012). Empowerment training and direct support professionals' attitudes about individuals with intellectual disabilities. *Journal of Intellectual Disabilities*, *16*(2), 119-125. <https://doi.org/doi:10.1177/1744629512443652>

Fleming, M. T., Lewis, T., Lyons, K., Poole, R., & Prillaman, C. (2022). The impact of diversity, equity, and inclusion training in an independent community oncology practice. *Journal of Clinical Oncology Conference: Annual Meeting of the American Society of Clinical Oncology, ASCO*, *40*(16). <https://doi.org/doi:10.1200/JCO.2022.40.16_suppl.11057>

Fletcher, A. M. C., & Akakpo, T. (2020). We can do better: Mitigating negatively racialized attitudes in child welfare through self-awareness training. *Child Welfare*, *98*(3), 1-23. <https://www.jstor.org/stable/48623663>

Foster, R., & Clark, J. M. (2015). Moderating the stereotypical views of health and social care students: The role of interprofessional education. *Journal of Interprofessional Care*, *29*(1), 34-40. <https://doi.org/doi:10.3109/13561820.2014.936059>

Friday, V. E. (2015). Exploring nursing students’ perceptions of older adults: The impact of gerontological education [ProQuest Information & Learning]. In *Dissertation Abstracts International Section A: Humanities and Social Sciences, 76*(4). Retrieved from <http://proxy.library.vcu.edu/login?url=https://search.ebscohost.com/login.aspx?direct=true&AuthType=ip,url,cookie,uid&db=psyh&AN=2015-99190-230&site=ehost-live&scope=site>

Friedman, C., & VanPuymbrouck, L. (2021). Impact of Occupational Therapy Education on Students' Disability Attitudes: A Longitudinal Study. *American Journal of Occupational Therapy*, *75*(4), 1-11. <https://doi.org/doi:10.5014/ajot.2021.047423>

Friedrich, B., Evans-Lacko, S., London, J., Rhydderch, D., Henderson, C., & Thornicroft, G. (2013). Anti-stigma training for medical students: The Education Not Discrimination project. *The British Journal of Psychiatry. Supplement*, *55*, s89-94. <https://doi.org/doi:10.1192/bjp.bp.112.114017>

Fruhauf, C. A., Jarrott, S. E., & Lambert-Shute, J. J. (2004). Service-learners at dementia care programs: An intervention for improving contact, comfort, and attitudes. *Gerontology & Geriatrics Education*, *25*(1), 37-52. <https://doi.org/doi:10.1300/J021v25n01_03>

Fuchs, J. R., Tannous, A. M., Kaul, P., & Jimenez, S. M. (2021). Incorporating implicit bias education and mitigation into cultural competence curriculum for first year medical students. *Journal of General Internal Medicine*, *36*, S445-S446.

Galletly, C., & Burton, C. (2011). Improving medical student attitudes towards people with schizophrenia. *The Australian and New Zealand Journal of Psychiatry*, *45*(6), 473-476. <https://doi.org/doi:10.3109/00048674.2011.541419>

Geibel, S., Hossain, S. M. I., Pulerwitz, J., Sultana, N., Hossain, T., Roy, S., Burnett-Zieman, B., Stackpool-Moore, L., Friedl, , B. A., Yasmin, R., Sadiq, N., & Yam, E. (2017). Stigma reduction training improves healthcare provider attitudes toward, and experiences of, young marginalized people in Bangladesh. *The Journal of Adolescent Health: Official Publication of the Society for Adolescent Medicine*, *60*(2), S35-S44. <https://doi.org/doi:10.1016/j.jadohealth.2016.09.026>

Genao, I., Bussey-Jones, J., St. George, D. M., & Corbie-Smith, G. (2009). Empowering students with cultural competence knowledge: Randomized controlled trial of a cultural competence curriculum for third-year medical students. *Journal of the National Medical Association*, *101*(12), 1241-1246. <https://doi.org/doi:10.1016/s0027-9684(15)31135-4>

Gendron, T., Cimarolli, V. R., Inker, J., Rhodes, A., Hennessa, A., ra, & Stone, R. (2021). The efficacy of a video-based intervention to reduce ageism among long-term services and supports staff. *Gerontology & Geriatrics Education*, *42*(3), 316-330. <https://doi.org/doi:10.1080/02701960.2021.1880904>

Gholamzadeh, S., Khastavaneh, M., Khademian, Z., & Ghadakpour, S. (2018). The effects of empathy skills training on nursing students' empathy and attitudes toward elderly people. *BMC Medical Education*, *18*(1), 198. <https://doi.org/doi:10.1186/s12909-018-1297-9>

Gillespie, C., Surkis, C., Minksy, M., & Adams, J. (2022). Incoming medical students' empathy orientation: Baseline empathy, association with initial clinical skills, and effects of empathy curriculum on overall clinical skill performance. *Journal of General Internal Medicine*, *37*, S214. <https://doi.org/doi:10.1007/s11606-022-07653-8>

Gillespie, G. L., Pritchard, T., Bankston, K., Burno, J., & Glazer, G. (2017). An evaluation of forums for discussions on inclusion in a college of nursing. *Nursing Outlook*, *65*(1), 103-115. <https://doi.org/doi:10.1016/j.outlook.2016.08.002>

Gilliam, C. A., Rooholamini, S., McPhillips, H., Mullett, T., Homer, P., McDade, J., Olszewski, A., ra, Willgerodt, M., & Grow, H. M. (2020). 88. Qualitative assessment of a novel equity, diversity and inclusion (EDI) curriculum for pediatric residents. *Academic Pediatrics*, *20*(7), e41-e42.

Gillian-Daniel, D. L., Petty, E. M., Schmid, M. E., Stahr, A., & Raymond, N. C. (2020). Addressing biases in health care by promoting inclusive teaching practices among faculty in health professions education programs: “Learning to say the right words at the right time”. *New Directions for Teaching and Learning*, *2020*(162), 123-133. <https://doi.org/doi:10.1002/tl.20397>

Gitlin, R. (2018). 44.3 From self-reflection to guided action: Multicultural identities, power, and privilege within the provider-patient relationship. *Journal of the American Academy of Child and Adolescent Psychiatry*, *57*(10), S64. <https://doi.org/doi:10.1016/j.jaac.2018.07.270>

Gleeson, S., Bathgate, M., Frederick, J., Desruisseaux, M. S., Meyer, J., Virata, M. D., Zapata, H., Shenoi, S., Radin, J., Golden, M., Trubin, P., Shaw, A., Friedl, , G., & Aoun-Barakat, L. A. (2021). Infectious disease diversity, equity, and antiracism (ID2EA): A dedicated curriculum for infectious disease professionals. *Open Forum Infectious Diseases*, *8*, S55. <https://doi.org/doi:10.1093/ofid/ofab466.087>

Gogineni, R. R., Aggarwal, N., Sharma, S., & Kallivayalil, R. (2022). Cultural formulation interview-Indian Context. *Indian Journal of Psychiatry*, *64*, S654.

Golden, A. G., van Zuilen, M. H., Mintzer, M. J., Issenberg, S. B., Silverman, M. A., & Roos, B. A. (2010). A fourth-year medical school clerkship that addressed negative attitudes toward geriatric medicine. *Journal of the American Geriatrics Society*, *58*(4), 746-750. <https://doi.org/doi:10.1111/j.1532-5415.2010.02774.x>

Gonzales, E., Morrow-Howell, N., & Gilbert, P. (2010). Changing medical students' attitudes toward older adults. *Gerontology & Geriatrics Education*, *31*(3), 220-234. <https://doi.org/doi:10.1080/02701960.2010.503128>

Gonzalez, C. M., Fisher, M., Ark, T. K., Kalet, A., Marantz, P. R., Burgess, D. J., Rodriguez, C. J., Samuel, M., Burd, L., & Milan, F. (2022). Moving beyond vignettes: A high-fidelity simulation for skill development and assessment in implicit bias recognition and management. *Journal of General Internal Medicine*, *37*, S643-S644. <https://doi.org/doi:10.1007/s11606-022-07653-8>

Gonzalez, C. M., Walker, S. A., Karp, E., Rodriguez, N., Noah, Y., & Marantz, P. R. (2018). Implicit bias recognition and management: Advancing from awareness to skills development. *Journal of General Internal Medicine*, *33*(2), 715.

Gonzalez, C. M., Walker, S. A., Rodriguez, N., Karp, E., & Marantz, P. R. (2020). It Can Be Done! A Skills-based elective in implicit bias recognition and management for preclinical medical students. *Academic Medicine: Journal of the Association of American Medical Colleges*, *95*(12), S150-S155. <https://doi.org/doi:10.1097/ACM.0000000000003697>

Gonzalez, C. M., Walker, S. A., Rodriguez, N., Noah, Y. S., & Marantz, P. R. (2021). Implicit bias recognition and management in interpersonal encounters and the learning environment: A skills-based curriculum for medical students. *MedEdPORTAL: The Journal of Teaching and Learning Resources*, *17*, 11168. <https://doi.org/doi:10.15766/mep_2374-8265.11168>

Gordon Perue, G. L., Fox-Rosellini, S. E., Sur, N. B., Marul, a-Londono, E., Margolesky, J., Tornes, L., Bure, A., Kalika, P. M., Chileuitt, A. A., Allespach, H., Uthman, B. M., Alkhachroum, A., Sacco, R. L., & Monteith, T. S. (2021). Development of an equity, diversity, inclusion, and anti-racism pledge as the foundation for action in an academic department of neurology. *Neurology*, *97*(15), 729-736. <https://doi.org/doi:10.1212/WNL.0000000000012674>

Greene, M. H. (2016). Advocacy to Reduce the Stigma: Intervention Project for Nurses. *The Florida Nurse*, *64*(1), 12.

Griner, S. B., Spears, E. C., & Maskey, S. (2022). Advancing cultural competency toward sexual and gender minorities: Innovation in maternal and child health pedagogy. *Maternal & Child Health Journal*, *26*(1), 42-48. <https://doi.org/doi:10.1007/s10995-021-03309-x>

Guh, J., Harris, C. R., Martinez, P., Chen, F. M., & Gianutsos, L. P. (2019). Antiracism in residency: A multimethod intervention to increase racial diversity in a community-based residency program. *Family Medicine*, *51*(1), 37-40. <https://doi.org/doi:10.22454/FamMed.2019.987621>

Guh, J., Krinsky, L., White-Davis, T., Sethi, T., Hayon, R., & Edgoose, J. (2020). Teaching racial affinity caucusing as a tool to learn about racial health inequity through an experiential workshop. *Family Medicine*, *52*(9), 656-660. <https://doi.org/doi:10.22454/FamMed.2020.596649>

Gutierrez, B., Kaatz, A., Chu, S., Ramirez, D., Samson-Samuel, C., & Carnes, M. (2014). "Fair play": A videogame designed to address implicit race bias through active perspective taking. *Games for Health Journal*, *3*(6), 371-378. <https://doi.org/doi:10.1089/g4h.2013.0071>

Guzder, J., & Rousseau, C. (2013). A diversity of voices: the McGill 'Working with Culture' seminars. *Culture, Medicine and Psychiatry*, *37*(2), 347-364. <https://doi.org/doi:10.1007/s11013-013-9316-0>

Hackett, A. N., Kramer, J. E., Leonard, S. R., Scotellaro, M. A., Voigt, B. L., Silvestri, J. M., Spagnoli, A., & Chubinskaya, S. (2018). Development of an innovative diversity and inclusion (D&I) educational program. *Academic Pediatrics*, *18*(5), e1. <https://doi.org/doi:10.1016/j.acap.2018.04.015>

Hagopian, A., McGlone West, K., Ornelas, I. J., Hart, A. N., Hagedorn, J., & Spigner, C. (2018). Adopting an anti-racism public health curriculum competency: The University of Washington experience. *Public Health Reports*, *133*(4), 507-513. <https://doi.org/doi:10.1177/0033354918774791>

Halloran, L. (2009). Teaching transcultural nursing through literature. *The Journal of Nursing Education*, *48*(9), 523-528. <https://doi.org/doi:10.3928/01484834-20090610-07>

Hamel, L. M., Robinson, L. M., Chamblee, M., Dougherty, D. W., & Sood, B. (2022). Designing and implementing an evidence-based and longitudinal implicit bias training curriculum for school of medicine members and healthcare providers. *Journal of Clinical Oncology. Conference: Annual Meeting of the American Society of Clinical Oncology, ASCO*, *40*(16). <https://doi.org/doi:10.1200/JCO.2022.40.16_suppl.e24136>

Hamilton-Mason, J., & Schneider, S. (2018). Antiracism expanding social work education: A qualitative analysis of the Undoing Racism workshop experience. *Journal of Social Work Education*, *54*(2), 337-348. <https://doi.org/doi:10.1080/10437797.2017.1404518>

Hannah, S. D., & Carpenter-Song, E. (2013). Patrolling your blind spots: Introspection and public catharsis in a medical school faculty development course to reduce unconscious bias in medicine. *Culture, Medicine and Psychiatry*, *37*(2), 314-339. <https://doi.org/doi:10.1007/s11013-013-9320-4>

Hardeman, R. R., Burgess, D., Murphy, K., Satin, D. J., Nielsen, J., Potter, T. M., Karbeah, J. M., Zulu-Gillespie, M., Apolinario-Wilcoxon, A., Reif, C., & Cunningham, B. A. (2018). Developing a medical school curriculum on racism: Multidisciplinary, multiracial conversations informed by public health critical race praxis (PHCRP). *Ethnicity & Disease*, *28*, 271-278. <https://doi.org/doi:10.18865/ed.28.S1.271>

Harris, R., Cormack, D., Curtis, E., Jones, R., Stanley, J., & Lacey, C. (2016). Development and testing of study tools and methods to examine ethnic bias and clinical decision-making among medical students in New Zealand: The bias and decision-making in medicine (BDMM) study. *BMC Medical Education*, *16*(1). <https://doi.org/doi:10.1186/s12909-016-0701-6>

Harrison-Bernard, L. M., Augustus-Wallace, A. C., Souza-Smith, F. M., Tsien, F., Casey, G. P., & Gunaldo, T. P. (2020). Knowledge gains in a professional development workshop on diversity, equity, inclusion, and implicit bias in academia. *Advances in Physiology Education*, *44*(3), 286-294. <https://doi.org/doi:10.1152/advan.00164.2019>

Harrison-Bernard, L. M., Souza-Smith, F. M., Augustus-Wallace, A. C., Tsien, F., & Casey, G. P. (2018). Diversity training workshop for graduate faculty and students. *FASEB Journal*, *32*(1). <https://doi.org/doi:10.1096/fasebj.2018.32.1_supplement.773.11>

Hawke, L. D., Michalak, E. E., Maxwell, V., & Parikh, S. V. (2014). Reducing stigma toward people with bipolar disorder: impact of a filmed theatrical intervention based on a personal narrative. *The International Journal of Social Psychiatry*, *60*(8), 741-750. <https://doi.org/doi:10.1177/0020764013513443>

Hernandez, R. A., Haidet, P., Gill, A. C., & Teal, C. R. (2013). Fostering students' reflection about bias in healthcare: Cognitive dissonance and the role of personal and normative standards. *Medical Teacher*, *35*(4), e1082-e1089. <https://doi.org/doi:10.3109/0142159X.2012.733453>

Heuer, S., Douglas, N., Burney, T., & Willer, R. (2020). Service-learning with older adults in care communities: Measures of attitude shifts in undergraduate students. *Gerontology & Geriatrics Education*, *41*(2), 186-199. <https://doi.org/doi:10.1080/02701960.2019.1596087>

Hillbrand, M., Hawkins, D., Howe, D. M., & Stayner, D. Through the eyes of another: Improving the skills of forensic providers using a consumer-informed role-play procedure. *Psychiatric Rehabilitation Journal*, *31*(3), 239-242. <https://doi.org/doi:10.2975/31.3.2008.239.242>

Hinners, C., & Potter, J. (2006). A partnership between the University of Nebraska College of Medicine and the community. *Gerontology & Geriatrics Education*, *27*(2), 83-91. <https://doi.org/doi:10.1300/J021v27n02_09>

Ho, M.-J., Lee, K.-L., & Gaufberg, E. (2008). Stigma: A curriculum to change attitudes, knowledge and skills. *Medical Education*, *42*(5), 530-531. <https://doi.org/doi:10.1111/j.1365-2923.2008.03049.x>

Hodgins, G., McSherry, M. L., Gibbs, E. P., Brosco, J., & Harvey, P. D. (2019). 3.37 The Debbie project: Reducing bias toward persons with disabilities. *Journal of the American Academy of Child and Adolescent Psychiatry*, *58*(10), S206-S207.

Hofmeister, S., & Soprych, A. (2017). Teaching resident physicians the power of implicit bias and how it impacts patient care utilizing patients who have experienced incarceration as a model. *International Journal of Psychiatry in Medicine*, *52*(4), 345-354. <https://doi.org/doi:10.1177/0091217417738935>

Holman, A. R., Dotson-Blake, K. P., Pope, M., Pangelinan, J. S., & Coker, A. D. (2011). Creating a sexual orientation and identity dialogue. In Pope, M., Pangelinan, J.S., & Coker A.D. (Eds.), *Experiential activities for teaching multicultural competence in counseling.* Alexandria, VA: American Counseling Association. Retrieved from <https://www.counseling.org/publications/frontmatter/72904-fm.pdf>

Holroyd, A., Dahlke, S., Fehr, C., Jung, P., & Hunter, A. (2009). Attitudes toward aging: Implications for a caring profession. *The Journal of Nursing Education*, *48*(7), 374-380. <https://doi.org/doi:10.3928/01484834-20090615-04>

Horst, A., Schwartz, B. D., Fisher, J. A., Michels, N., & Van Winkle, L. J. (2019). Selecting and performing service-learning in a team-based learning format fosters dissonance, reflective capacity, self-examination, bias mitigation, and compassionate behavior in prospective medical students. *International Journal of Environmental Research and Public Health*, *16*(20), 3926. <https://doi.org/doi:10.3390/ijerph16203926>

Hughes, K. H., & Hood, L. J. (2007). Teaching methods and an outcome tool for measuring cultural sensitivity in undergraduate nursing students. *Journal of Transcultural Nursing: Official Journal of the Transcultural Nursing Society*, *18*(1), 57-62. <https://doi.org/doi:10.1177/1043659606294196>

Hui, K., Sukhera, J., Vigod, S., Taylor, V. H., & Zaheer, J. (2020). Recognizing and addressing implicit gender bias in medicine. *CMAJ*, *192*(42), E1269-E1270. <https://doi.org/doi:10.1503/cmaj.200286>

Ilyenkova, V., Kunitskaya, S., & Eramova, I. (2012). HIV-AIDS stigma and discrimination in health care sector in Belarus. *Retrovirology*, *9*(Suppl 1), P81. <https://doi.org/doi:10.1186/1742-4690-9-S1-P81>

Isom, K. A. (2021). The effect of an educational video intervention on knowledge of obesity and weight bias in dietetic interns: A mixed methods analysis [ProQuest Information & Learning]. *Dissertation Abstracts International: Section B: The Sciences and Engineering, 82*(1B). Retrieved from <http://proxy.library.vcu.edu/login?url=https://search.ebscohost.com/login.aspx?direct=true&AuthType=ip,url,cookie,uid&db=psyh&AN=2020-58781-249&site=ehost-live&scope=site>

Jabson, J. M., Mitchell, J. W., & Doty, S. B. (2016). Associations between non-discrimination and training policies and physicians' attitudes and knowledge about sexual and gender minority patients: a comparison of physicians from two hospitals. *BMC Public Health*, *16*, 256. <https://doi.org/doi:10.1186/s12889-016-2927-y>

Jackson, M. A., Pope, M., Pangelinan, J. S., & Coker, A. D. (2011). Multicultural career case conceptualization role-plays: Exploring hidden biases through reflections, video-recording, and small-group processing. In Pope, M., Pangelinan, J.S., & Coker A.D. (Eds.), *Experiential activities for teaching multicultural competence in counseling.* Alexandria, VA: American Counseling Association.. Retrieved from <https://www.counseling.org/publications/frontmatter/72904-fm.pdf>

Jackson, R. A., McCloskey, K. A., & McHaelen, R. P. (2011). *A sexuality & gender diversity training program: Increasing the competency of mental health professionals*. Professional Resource Press/Professional Resource Exchange.

Jackson, S. R. (2015). Pre-service school psychologists' racial and weight-related biases and the relationship that taking diversity courses has on these biases ProQuest Information & Learning]. In *Dissertation Abstracts International Section A: Humanities and Social Sciences*, *76*(5A). Retrieved from <http://proxy.library.vcu.edu/login?url=https://search.ebscohost.com/login.aspx?direct=true&AuthType=ip,url,cookie,uid&db=psyh&AN=2015-99210-142&site=ehost-live&scope=site>

Jahnke, S., Philipp, K., & Hoyer, J. (2015). Stigmatizing attitudes towards people with pedophilia and their malleability among psychotherapists in training. *Child Abuse & Neglect*, *40*, 93-102. <https://doi.org/doi:10.1016/j.chiabu.2014.07.008>

Janouskova, M., Formanek, T., Zrneckova, M., Alexova, A., Hejzlar, M., Chrtkova, D., Vitkova, M., Roboch, Z., & Motlova, L. B. (2019). How to reduce stigmatization of people with mental illness in medical education: READ intervention. *Jak omezovat stigmatizaci lidi s dusevnim onemocnenim pri vyuce lekarstvi: intervence READ.*, *Casopis lekaru ceskych, 158*(3-4), 151–155.

Jansen, D. A., & Morse, W. A. (2004). Positively influencing student nurse attitudes toward caring for elders: results of a curriculum assessment study. *Gerontology & Geriatrics Education*, *25*(2), 1-14. <https://doi.org/doi:10.1300/J021v25n02_01>

Jarris, Y. S., Bartleman, A., Hall, E. C., & Lopez, L. (2012). A preclinical medical student curriculum to introduce health disparities and cultivate culturally responsive care. *Journal of the National Medical Association*, *104*(9), 404-411. <https://doi.org/doi:10.1016/s0027-9684(15)30193-0>

Javier, D., Solis, L. G., Paul, M. F., Thompson, E. L., Maynard, G., Latif, Z., Stinson, K., Ahmed, T., & Vishwanatha, J. K. (2022). Implementation of an unconscious bias course for the National Research Mentoring Network. *BMC Medical Education*, *22*(1), 391. <https://doi.org/doi:10.1186/s12909-022-03466-9>

Jaworsky, D., Gardner, S., Thorne, J. G., Sharma, M., McNaughton, N., Paddock, S., Chew, D., Lees, R., Makuwaza, T., Wagner, A., Rachlis, A., & CHIME Research Group (2017). The role of people living with HIV as patient instructors - reducing stigma and improving interest around HIV care among medical students. *AIDS Care*, *29*(4), 524–531. <https://doi.org/10.1080/09540121.2016.1224314>

Jeste, D. V., Avanzino, J., Depp, C. A., Gawronska, M., Tu, X., Sewell, D. D., & Huege, S. F. (2018). Effect of short-term research training programs on medical students' attitudes toward aging. *Gerontology & Geriatrics Education*, *39*(2), 214-222. <https://doi.org/doi:10.1080/02701960.2017.1340884>

Jones, C. A., & Forhan, M. (2021). Addressing weight bias and stigma of obesity amongst physiotherapists. *Physiotherapy Theory and Practice*, *37*(7), 808-816. <https://doi.org/doi:10.1080/09593985.2019.1648623>

Kaatz, A., Filut, A., Her, Y., Pankey, T., Carnes, M., Stahr, A., Alex, er, L., & Kolehmainen, C. J. (2017). Evaluation of a workshop intervention to reduce racial bias in internal medicine residents' clinical decision-making. *Journal of General Internal Medicine*, *32*(2), S676-S677.

Kaf, W. A., Barboa, L. S., Fisher, B. J., & Snavely, L. A. (2011). Effect of interdisciplinary service learning experience for audiology and speech-language pathology students working with adults with dementia. *American Journal of Audiology*, *20*(2), S241-S249. <https://doi.org/doi:10.1044/1059-0889(2011/10-0025)>

Kallianos, K. G., Webb, E. M., Hess, C. P., Talbott, J., & Bucknor, M. D. (2019). Use of the Implicit Association Test to improve diversity in radiology. *Journal of the American College of Radiology*, *16*(7), 976-979. <https://doi.org/doi:10.1016/j.jacr.2019.01.010>

Kantor, B., & Myers, M. (2006). From aging... to saging- the Ohio State Senior Partners Program: longitudinal and experiential geriatrics education. *Gerontology & Geriatrics Education*, *27*(2), 69-74. <https://doi.org/doi:10.1300/J021v27n02_08>

Karayigit, C., & Ozier, M. (2021). Using character connection journaling to develop cultural empathy. *British Journal of Guidance and Counselling,* 1-12. <https://doi.org/doi:10.1080/03069885.2021.1961210>

Kassam, A., Glozier, N., Leese, M., Loughran, J., & Thornicroft, G. (2011). A controlled trial of mental illness related stigma training for medical students. *BMC Medical Education*, *11*, 51. <https://doi.org/doi:10.1186/1472-6920-11-51>

Khan, N. (2019). Improving weight management skills in trainees. *Journal of General Internal Medicine*, *34*(2), S818S819.

Khidir, H., Carson, S., Vassar, S. D., Nafisi, A., Abdullahi, G., Shah, S., Belani, H., Meza, B., Brown, A. F., & Gonzalez, C. M. (2022). Effects of a longitudinal intervention to improve clinical educator implicit bias recognition and management in teaching and clinical settings. *Journal of General Internal Medicine*, *37*, S621. <https://doi.org/doi:10.1007/s11606-022-07653-8>

Kidd, J. D., Bockting, W., Cabaniss, D. L., & Blumenshine, P. (2016). Special-"T" training: Extended follow-up results from a residency-wide professionalism workshop on transgender health. *Academic Psychiatry: The Journal of the American Association of Directors of Psychiatric Residency Training and the Association for Academic Psychiatry*, *40*(5), 802-806. <https://doi.org/doi:10.1007/s40596-016-0570-7>

Kim, S.-H., Canfield, J., & Harley, D. (2019). Using photovoice as a method to examine microaggressions: Conceptualizing culturally competent practice and curriculum with Asian Americans. *Journal of Human Behavior in the Social Environment*. *29*(8), 1036-1043. <https://doi.org/doi:10.1080/10911359.2019.1658684>

Kistin, C. J. (2015). Address persistent racial disparities in academic medicine to improve healthcare quality. *Evidence-Based Medicine*, *20*(6), 191-192. <https://doi.org/doi:10.1136/ebmed-2015-110308>

Klein, E. W., & Nakhai, M. (2016). Caring for LGBTQ patients: Methods for improving physician cultural competence. *International Journal of Psychiatry in Medicine*, *51*(4), 315-324. <https://doi.org/doi:10.1177/0091217416659268>

Kleinschmidt, S., Farl, M., , A., & Welsh, L. (2020). The "favorite patient" exercise: A nonconfrontational curriculum to address bias in emergency medicine students. *Academic Emergency Medicine*, *27*, S326.

Kliot, T., Khandani, A., Tekeste, R., Mayo, R., & Carter, R. (2021). Integrating a social justice curriculum in to resident noon conference series. *Pediatrics. Conference: National Conference and Exhibition Meeting of the American Academy of Pediatrics, AAP*, *149*.

Knoeckel, J., Sarcone, E., & Stella, S. A. (2018). The health equity pathway: Development of a novel curriculum addressing the health inequities encountered in internal medicine training. *Journal of General Internal Medicine*, *33*(2), 744.

Koch, A., Ritz, M., Morrow, A., Grier, K., & McMillian-Bohler, J. M. (2021). Role-play simulation to teach nursing students how to provide culturally sensitive care to transgender patients. *Nurse Education in Practice*, *54,* 103123. <https://doi.org/doi:10.1016/j.nepr.2021.103123>

Kogan, L. R., & Schoenfeld-Tacher, R. M. (2018). Participation in an intergenerational service learning course and implicit biases. *Innovation in Aging, 3*(Suppl 1), S201. <https://doi.org/10.1093/geroni/igz038.728>

Kohrt, B. A., Jordans, M. J. D., Turner, E. L., Sikkema, K. J., Luitel, N. P., Rai, S., Singla, D. R., Lamichhane, J., Lund, C., & Patel, V. (2018). Reducing stigma among healthcare providers to improve mental health services (RESHAPE): Protocol for a pilot cluster randomized controlled trial of a stigma reduction intervention for training primary healthcare workers in Nepal. *Pilot and Feasibility Studies*, *4*(1). <https://doi.org/doi:10.1186/s40814-018-0234-3>

Kushner, R. F., Zeiss, D. M., Feinglass, J. M., & Yelen, M. (2014). An obesity educational intervention for medical students addressing weight bias and communication skills using standardized patients. *BMC Medical Education*, *14*, 53. <https://doi.org/doi:10.1186/1472-6920-14-53>

Lam, T. P., Lam, K. F., Lam, E. W. W., & Sun, K. S. (2015). Does postgraduate training in community mental health make a difference to primary care physicians' attitudes to depression and schizophrenia? *Community Mental Health Journal*, *51*(6), 641-646. <https://doi.org/doi:10.1007/s10597-015-9829-9>

Lapshin, O., Wasserman, E., & Finkelstein, J. (2006). Computer intervention to decrease level of psychiatric stigma among medical students. *AMIA Annual Symposium Proceedings. AMIA Symposium*, 998.

Lawlis, S. M., Darby-McClure, R., Darden, A. G., & Curran, K. A. (2022). 44. Expansion and integration of a novel LGBTQ+ curriculum into the pre-clinical medical education at a midwestern school. *Journal of Adolescent Health*, *70*, S24. <https://doi.org/doi:10.1016/j.jadohealth.2022.01.157>

Lee, J. K., Spencer, J., & Tomasa, L. (2018). A didactic + experiential course to enhance aging competencies. *Journal of the American Geriatrics Society*, *66*, S71.

Lenes, E., Swank, J. M., Hart, K. A., Machado, M. M., Darilus, S., Ardelt, M., Smith‐Adcock, S., Rockwood Lane, M., & Puig, A. (2020). Color‐conscious multicultural mindfulness training in the counseling field. *Journal of Counseling & Development*, *98*(2), 147-158. <https://doi.org/10.1002/jcad.12309>

Preston Lewis, C., Corley, D. J., Lake, N., Brockopp, D., & Moe, K. (2015). Overcoming barriers to effective pain management: The use of professionally directed small group discussions. *Pain Management Nursing*, *16*(2), 121-127. <https://doi.org/doi:10.1016/j.pmn.2014.05.002>

Li, J., Li, J., Thornicroft, G., Yang, H., Chen, W., & Huang, Y. (2015). Training community mental health staff in Guangzhou, China: evaluation of the effect of a new training model. *BMC Psychiatry*, *15*, 263. <https://doi.org/doi:10.1186/s12888-015-0660-1>

Li, L., Guan, J., Liang, L.-J., Lin, C., & Wu, Z. (2013). Popular opinion leader intervention for HIV stigma reduction in health care settings. *AIDS Education and Prevention*, *25*(4), 327-335. <https://doi.org/doi:10.1521/aeap.2013.25.4.327>

Li, L., Wu, Z., Liang, L.-J., Lin, C., Guan, J., Jia, M., Rou, K., & Yan, Z. (2013). Reducing HIV-related stigma in health care settings: a randomized controlled trial in China. *American Journal of Public Health*, *103*(2), 286-292. <https://doi.org/doi:10.2105/AJPH.2012.300854>

Li, Y., Sorrentino, R., Norman, R., Hampson, E., & Ye, Y. (2017). Effects of symptom versus recovery video, similarity, and uncertainty orientation on the stigmatization of schizophrenia. *Personality and Individual Differences*, *106*, 117-121. <https://doi.org/10.1016/j.paid.2016.10.050>

Lie, D., Boker, J., Clevel, & , E. (2006). Using the tool for assessing cultural competence training (TACCT) to measure faculty and medical student perceptions of cultural competence instruction in the first three years of the curriculum. *Academic Medicine: Journal of the Association of American Medical Colleges*, *81*(6), 557-564. <https://doi.org/doi:10.1097/01.ACM.0000225219.53325.52>

Lightfoot, A., Chapman, M., Thatcher, K., Coyne-Beasley, T., Colby, R., Eng, E., & Siman, F. (2015). Envisioning health: A trans-disciplinary, community engaged visual intervention for healthcare providers on implicit bias toward Latino/a immigrant youth. *Journal of Adolescent Health*, *56*(2), S91. <https://doi.org/doi:10.1016/j.jadohealth.2014.10.182>

Lincoln, T. M., Arens, E., Berger, C., & Rief, W. (2008). Can antistigma campaigns be improved? A test of the impact of biogenetic vs psychosocial causal explanations on implicit and explicit attitudes to schizophrenia. *Schizophrenia Bulletin*, *34*(5), 984-994. <https://doi.org/doi:10.1093/schbul/sbm131>

Lohiniva, A.-L., Benkirane, M., Numair, T., Mahdy, A., Saleh, H., Zahran, A., Okasha, O., Talaat, M., & Kamal, W. (2016). HIV stigma intervention in a low-HIV prevalence setting: A pilot study in an Egyptian healthcare facility. *AIDS Care*, *28*(5), 644-652. <https://doi.org/doi:10.1080/09540121.2015.1124974>

Loignon, C., Boudreault-Fournier, A., Truchon, K., Labrousse, Y., & Fortin, B. (2014). Medical residents reflect on their prejudices toward poverty: a photovoice training project. *BMC Medical Education*, *14*, 1050. <https://doi.org/doi:10.1186/s12909-014-0274-1>

Long, A., Jennings, J., Bademosi, K., Ch, ran, A., Sawyer, S., Schumacher, C., Greenbaum, A., & Fields, E. L. (2021). Storytelling to improve healthcare worker understanding, beliefs, and practices related to LGBTQ + patients: A program evaluation. *Evaluation and Program Planning* *90*, 101979. <https://doi.org/doi:10.1016/j.evalprogplan.2021.101979>

Lopez-Villegas, A., Lopez-Liria, R., Rocamora-Perez, P., & Pezzella, A. (2020). Educational policies and legislation at European level for LGBT+ inclusive education: Building a MOOC. *European Geriatric Medicine*, *11*, S188.

Lucas, M. S. (2018). Addressing cancer pain inequities through intervention. *Oncology Nursing Forum*, *45*(2), 141-142. <https://doi.org/doi:10.1188/18.ONF.141-142>

Lund, A., Latortue, K. Y., & Rodriguez, J. (2021). Dietetic training: Understanding racial inequity in power and privilege. *Journal of the Academy of Nutrition & Dietetics*, *121*(8), 1437-1440. <https://doi.org/doi:10.1016/j.jand.2020.09.041>

Madianos, M. G., Priami, M., Alevisopoulos, G., Koukia, E., & Rogakou, E. (2005). Nursing students' attitude change towards mental illness and psychiatric case recognition after a clerkship in psychiatry. *Issues in Mental Health Nursing*, *26*(2), 169-183. <https://doi.org/doi:10.1080/01612840590901635>

Madzima, S., & Crawford, T. (2021). Historically informed medical care: Revisiting historical racial injustices to educate and challenge negative stereotypes that contribute to inequitable care and health disparities. *Journal of General Internal Medicine*, *36*, S424-S425.

Magliano, L., Read, J., Sagliocchi, A., ra, Oliviero, N., D'Ambrosio, A., Campitiello, F., Zaccaro, A., Guizzaro, L., & Patalano, M. (2014). "Social dangerousness and incurability in schizophrenia": results of an educational intervention for medical and psychology students. *Psychiatry research*, *219*(3), 457-463. <https://doi.org/doi:10.1016/j.psychres.2014.06.002>

Magliano, L., Rinaldi, A., Costanzo, R., De Leo, R., Schioppa, G., Petrillo, M., & Read, J. (2016). Improving psychology students' attitudes toward people with schizophrenia: A quasi-randomized controlled study. *The American Journal of Orthopsychiatry*, *86*(3), 253-264. <https://doi.org/doi:10.1037/ort0000161>

Mahmoud, K. F., Lindsay, D., Scolieri, B. B., Hagle, H., Puskar, K. R., & Mitchell, A. M. (2018). Changing BSN students' stigma toward patients who use alcohol and opioids through screening, brief intervention, and referral to treatment (SBIRT) education and training: A pilot study. *Journal of the American Psychiatric Nurses Association*, *24*(6), 510-521. <https://doi.org/doi:10.1177/1078390317751624>

Malott, K. M., & de Zaid, M. H. (2007). Gender bias in Guatemalan counselor education students: Using an experimental activity as a stimulus. *International Journal for the Advancement of Counselling*, *29*(1), 33-42. <https://doi.org/doi:10.1007/s10447-006-9026-1>

Manchanda, E., Chary, A., Molina, M., Dadabhoy, F., & Landry, A. (2020). Resident-led health equity curriculum. *Western Journal of Emergency Medicine*, *21*(4), S54-S55.

Marlow, E., Nosek, M., Lee, Y., Young, E., Bautista, A., ra, & Hansen, F. T. (2015). Nurses, formerly incarcerated adults, and Gadamer: phronesis and the Socratic dialectic. *Nursing philosophy : an international journal for healthcare professionals*, *16*(1), 19-28. <https://doi.org/doi:10.1111/nup.12055>

Martinez, J., Conigliaro, J., Pho, A. T., Jalali, C., Morales, S., Boutin-Foster, C., & Ghesquiere, A. (2016). A longitudinal psychosocial curriculum to enhance residents' self-efficacy in delivering culturally competent care. *Journal of General Internal Medicine*, *31*(2), S97.

Martinez, J., Friedman, K., Spielmann, K., Litwok, Y., & Katona, K. (2017). Mitigating unconscious bias in resident applicant interviews. *Journal of General Internal Medicine*, *32*(2), S692.

Martinez, L. R., Snoeyink, M. J., Hamilton, K. M., Nordstrom, A. H., & Goodfriend, W. (2022). An ally skill-building workshop. In Nordstrom, A., & Goodfriend, W. (Eds.), *Innovative stigma and discrimination reduction programs across the world*. Routledge/Taylor & Francis Group.

Martinez-Martinez, C., Sanchez-Martinez, V., Sales-Orts, R., Dinca, A., Richart-Martinez, M., & Ramos-Pichardo, J. D. (2019). Effectiveness of direct contact intervention with people with mental illness to reduce stigma in nursing students. *International Journal of Mental Health Nursing*, *28*(3), 735-743. <https://doi.org/doi:10.1111/inm.12578>

Massé, J., Dupéré, S., Martin, É., & Lévesque, M. C. (2020). Transformative medical education: must

community-based traineeship experiences be part of the curriculum? A qualitative study.

*International Journal for Equity in Health, 19*(1), 94. <https://doi.org/10.1186/s12939-020-01213-4>

Masters, C., Robinson, D., Faulkner, S., Patterson, E., McIlraith, T., & Ansari, A. (2019). Addressing biases in patient care with the 5Rs of cultural humility, a clinician coaching tool. *Journal of General Internal Medicine*, *34*(4), 627-630. <https://doi.org/doi:10.1007/s11606-018-4814-y>

Mayers, P. (2007). Introducing human rights and health into a nursing curriculum. *Curationis*, *30*(4), 53-60. <https://doi.org/doi:10.4102/curationis.v30i4.1117>

McCave, E. L., Aptaker, D., Hartmann, K. D., & Zucconi, R. (2019). Promoting affirmative transgender health care practice within hospitals: An IPE standardized patient simulation for graduate health care learners. *MedEdPORTAL*, *15*, 10861. <https://doi.org/doi:10.15766/mep_2374-8265.10861>

McCleary-Gaddy, A. T., & Scales, R. (2019). Addressing mental illness stigma, implicit bias, and stereotypes in medical school. *Academic Psychiatry*, *43*(5), 512-515. <https://doi.org/doi:10.1007/s40596-019-01081-3>

McClinton, A., & Laurencin, C. T. (2020). Just in TIME: Trauma-informed medical education. *Journal of Racial and Ethnic Health Disparities*, *7*(6), 1046-1052. <https://doi.org/doi:10.1007/s40615-020-00881-w>

McCool, M. A., Jr., Du Toit, F., Petty, C. R., & McCauley, C. (2006). The impact of a program of prejudice-reduction seminars in South Africa. *Journal of Applied Social Psychology*, *36*(3), 586-613. <https://doi.org/doi:10.1111/j.0021-9029.2006.00020.x>

McCray, N. (2021). Inside job: Examining the influence of training on mental health stigma and social distancing among psychology trainees [ProQuest Information & Learning]. *Dissertation Abstracts International: Section B: The Sciences and Engineering, 82*(1B). Retrieved from <http://proxy.library.vcu.edu/login?url=https://search.ebscohost.com/login.aspx?direct=true&AuthType=ip,url,cookie,uid&db=psyh&AN=2020-67313-226&site=ehost-live&scope=site>

McDowell, T., Fang, S.-R., Young, C. G., Khanna, A., Sherman, B., & Brownlee, K. (2003). Making space for racial dialogue: Our experience in a marriage and family therapy training program. *Journal of Marital and Family Therapy*, *29*(2), 179-194. <https://doi.org/doi:10.1111/j.1752-0606.2003.tb01199.x>

McGervey, M., Mehdi, A., & Spencer, A. (2020). Getting bang for your buck in clinical reasoning: Innovative peer-to-peer teaching utilizing feedback loops and metacognition. *Journal of General Internal Medicine*, *35*, S730.

McKinley, S. K., Wang, L. J., Gartl, , R. M., Westfal, M. L., Costantino, C. L., Schwartz, D., Merrill, A. L., Petrusa, E., Lillemoe, K., Phitayakorn, R., & Massachusetts General Hospital Gender Equity Task, F. (2019). "Yes, I'm the doctor": One department's approach to assessing and addressing gender-based discrimination in the modern medical training era. *Academic Medicine: Journal of the Association of American Medical Colleges*, *94*(11), 1691-1698. <https://doi.org/doi:10.1097/ACM.0000000000002845>

McQuade, B. M., Schwartz, A., & Jarrett, J. B. (2021). Acknowledging and assessing implicit bias among healthcare professionals: A randomized controlled trial. *Journal of Interprofessional Education and Practice*, *24,* 100452. <https://doi.org/10.1016/j.xjep.2021.100452>

Meltzer, E. C., Suppes, A., Burns, S., Shuman, A., Orfanos, A., Sturiano, C. V., Charney, P., & Fins, J. J. (2013). Stigmatization of substance use disorders among internal medicine residents. *Substance Abuse*, *34*(4), 356-362. <https://doi.org/doi:10.1080/08897077.2013.815143>

Merritt, R., & Rougas, S. (2018). Multidisciplinary approach to structural competency teaching. *Medical Education*, *52*(11), 1191-1192. <https://doi.org/doi:10.1111/medu.13692>

Mette, M., & Hanze, M. (2021). Arrogant or caring? Influence of transactive communication in interprofessional learning on knowledge gains and stereotype changes. *GMS Journal for Medical Education*, *38*(3). <https://doi.org/doi:10.3205/zma001462>

Michaels, P. J., Corrigan, P. W., Buchholz, B., Brown, J., Arthur, T., Netter, C., & Macdonald-Wilson, K. L. (2014). Changing stigma through a consumer-based stigma reduction program. *Community Mental Health Journal*, *50*(4), 395-401. <https://doi.org/doi:10.1007/s10597-013-9628-0>

Mistler, B. J., Pope, M., Pangelinan, J. S., & Coker, A. D. (2011). I imagine you imagine I am and I observe, I imagine. In Pope, M., Pangelinan, J.S., & Coker A.D. (Eds.), *Experiential activities for teaching multicultural competence in counseling.* Alexandria, VA: American Counseling Association. Retrieved from <https://www.counseling.org/publications/frontmatter/72904-fm.pdf>

Mittal, D., Owen, R. R., Ounpraseuth, S., Chekuri, L., Drummond, K. L., Jennings, M. B., Smith, J. L., Sullivan, J. G., & Corrigan, P. W. (2020). Targeting stigma of mental illness among primary care providers: Findings from a pilot feasibility study. *Psychiatry Research*, *284*, 112641. <https://doi.org/doi:10.1016/j.psychres.2019.112641>

Mkwandawire‐Valhmu, L., Weitzel, J., Dressel, A., Neiman, T., Hafez, S., Olukotun, O., Kreuziger, S., Scheer, V., Washington, R., Hess, A., Morgan, S., & Stevens, P. (2019). Enhancing cultural safety among undergraduate nursing students through watching documentaries. *Nursing Inquiry*, *26*(1), 1-11. <https://doi.org/doi:10.1111/nin.12270>

Molina, M. F., Landry, A. I., Chary, A. N., & Burnett-Bowie, S. A. M. (2020). Addressing the elephant in the room: Microaggressions in medicine. *Annals of Emergency Medicine*, *76*(4), 387-391. <https://doi.org/doi:10.1016/j.annemergmed.2020.04.009>

Molloy, M. A., Sabol, V. K., Silva, S. G., & Guimond, M. E. (2016). Using trigger films as a bariatric sensitivity intervention: Improving nursing students' attitudes and beliefs about caring for obese patients. *Nurse Educator*, *41*(1), 19-24. <https://doi.org/doi:10.1097/NNE.0000000000000225>

Moriello, G. R., Smey, J. W., Pescatello, L. S., & Murphy, M. A. (2005). Influence of an educational intervention on pre-allied health students' attitudes toward older adults. *Gerontology & Geriatrics Education*, *25*(3), 1-11. <https://doi.org/doi:10.1300/J021v25n03_01>

Morris, J. E., Cribb Fabersunne, C., Scott, N., & Saldana, F. (2018). Teaching to undo structural racism. *Medical Education*, *52*(5), 552-553. <https://doi.org/doi:10.1111/medu.13550>

Mueller, B. (2022). Professional nurse attitudes of poverty before and after participation in a poverty simulation [ProQuest Information & Learning]. *Dissertation Abstracts International: Section B: The Sciences and Engineering, 83*(1B). Retrieved from <http://proxy.library.vcu.edu/login?url=https://search.ebscohost.com/login.aspx?direct=true&AuthType=ip,url,cookie,uid&db=psyh&AN=2021-80519-142&site=ehost-live&scope=site>

Muenks, E. (2022). Applying the multicultural orientation with cancer care teams: Talking the talk and walking the walk. *Psycho-Oncology*, *31*, 21. <https://doi.org/doi:10.1002/pon.5873>

Munoz, R. T., Miller, C. R., Fritz, T. A., & Miller, P. M. (2019). The learning process of social work students after exposure to an SBIRT training. *Journal of Social Work Practice in the Addictions*, *19*(1), 92-99. <https://doi.org/doi:10.1080/1533256X.2019.1590705>

Murray-García, J. L., Ngo, V., Yonn-Brown, T. A., Hosley, D. H., & Ton, H. (2022). California’s central valley: Teaching social determinants of health and cultural humility through an interprofessional, overnight road trip. *Journal of Health Care for the Poor and Underserved*, *33*(2), 819-841. <https://doi.org/doi:10.1353/hpu.2022.0066>

Muzyk, A., Mullan, P., Andolsek, K., Derouin, A., Smothers, Z., ers, C., & Holmer, S. (2020). A pilot interprofessional course on substance use disorders to improve students' empathy and counseling skills. *American Journal of Pharmaceutical Education*, *84*(4), 7415. <https://doi.org/doi:10.5688/ajpe7415>

Nadan, Y. (2016). Teaching note—Revisiting stereotypes: Enhancing cultural awareness through a web-based tool. *Journal of Social Work Education*, *52*(1), 50-56. <https://doi.org/doi:10.1080/10437797.2016.1113054>

Nadkarni, M., Harris, D., Uthlaut, B., Arant, E., Becker, D., & Martin, B. (2020). Teaching social determinants in the real world: A novel resident rotation. *Journal of General Internal Medicine*, *35*, S771.

Nagasawa, P. R., Harris, T., Bester, V. S., Bolden, A. R., Tshuma, L., Ryujin, D., Murray, D., Brown, S. D., & Sturges, D. (2021). "I can't breathe"--Courageous conversations and responses to racism in physician assistant education. *Journal of Physician Assistant Education (Lippincott Williams & Wilkins)*, *32*(2), 127-130. <https://doi.org/doi:10.1097/JPA.0000000000000358>

Narayanasamy, A. (2014). Review: ‘Work in progress’: Nurse educators’ views on preparing pre-registration nursing students in Wales for practice in multi-ethnic environments. *Journal of Research in Nursing*, *19*(6), 502-503. <https://doi.org/doi:10.1177/1744987114546929>

Nelson, S. (2016). Race, racism, and health disparities: What can i do about it? *Creative Nursing*, *22*(3), 161-165. <https://doi.org/doi:10.1891/1078-4535.22.3.161>

Ng, K. Y. Y., Leung, G. Y. C., Tey, A. J.-Y., Chaung, J. Q., Lee, S. M., Soundararajan, A., Yow, K. S., Ngiam, N. H. W., Lau, T. C., Wong, S. F., Wong, C. H., & Koh, G. C.-H. (2020). Bridging the intergenerational gap: the outcomes of a student-initiated, longitudinal, inter-professional, inter-generational home visit program. *BMC Medical Education*, *20*(1), 148. <https://doi.org/doi:10.1186/s12909-020-02064-x>

Ng, S., Kessler, L., Srivastava, R., Dusek, J., Duncan, D., Tansey, M., & Jeffs, L. (2010). Growing practice specialists in mental health: Addressing stigma and recruitment with a nursing residency program. *Nursing Leadership (Toronto, Ont.)*, *23*, 101-112. <https://doi.org/doi:10.12927/cjnl.2010.21750>

Nguyen, E., Chen, T. F., & O'Reilly, C. L. (2012). Evaluating the impact of direct and indirect contact on the mental health stigma of pharmacy students. *Social Psychiatry and Psychiatric Epidemiology*, *47*(7), 1087-1098. <https://doi.org/doi:10.1007/s00127-011-0413-5>

Nickel, F., Tapking, C., Benner, L., Schuler, S., Ottawa, G. B., Krug, K., Muller-Stich, B. P., & Fischer, L. (2019). Video teaching leads to improved attitudes towards obesity- A randomized study with 949 participants. *Obesity Surgery*, *29*(7), 2078-2086. <https://doi.org/doi:10.1007/s11695-019-03804-9>

Nnoromele, C. C., Olezene, C. S., Chen, Y. T., Gebrekristos, B. T., Blauwet, C., & Silver, J. K. (2021). Let's talk: Resident directed curriculum on diversity, equity and inclusion. *PM and R*, *13*, S221. <https://doi.org/doi:10.1002/pmrj.12735>

Noone, J. (2022). Preparing nurse educators to teach social determinants of health using backward design. *Journal of Nursing Education*, *61*(9), 511-515. <https://doi.org/doi:10.3928/01484834-20220705-05>

Norlock, F., Sadowski, L., & Kapolnek, M. (2014). A decade of change in attitudes toward the homeless among primary care internal medicine residents. *Journal of General Internal Medicine*, *29*, S500-S501.

Nova, E. A., McGeorge, C. R., & Stone Carlson, T. (2013). Bisexuality and lesbian, gay, and bisexual affirmative training: An exploration of family therapy students' beliefs and clinical experiences. *Journal of Feminist Family Therapy*, *25*(4), 212-232. <https://doi.org/doi:10.1080/08952833.2013.777886>

Nyblade, L., Addo, N. A., Atuahene, K., Alsoufi, N., Gyamera, E., Jacinthe, S., Leonard, M., Mingkwan, P., Stewart, C., Vormawor, R., & Kraemer, J. D. (2020). Results from a difference-in-differences evaluation of health facility HIV and key population stigma-reduction interventions in Ghana. *Journal of the International AIDS Society*, *23*(4), e25483. <https://doi.org/doi:10.1002/jia2.25483>

O'Brien, K. S., Puhl, R. M., Latner, J. D., Mir, A. S., & Hunter, J. A. (2010). Reducing anti-fat prejudice in preservice health students: A randomized trial. *Obesity*, *18*(11), 2138-2144. <https://doi.org/doi:10.1038/oby.2010.79>

O Carroll, A., & O'Reilly, F. (2019). Medicine on the margins. An innovative GP training programme prepares GPs for work with underserved communities. *Education for Primary Care: An Official Publication of the Association of Course Organisers, National Association of GP Tutors, World Organisation of Family Doctors*, *30*(6), 375-380. <https://doi.org/doi:10.1080/14739879.2019.1670738>

O'Connor, K., Brennan, D., O' Loughlin, K., Wilson, L., Pillay, D., Clarke, M., Casey, P., Malone, K., & Lane, A. (2013). Attitudes towards patients with mental illness in Irish medical students. *Irish Journal of Medical Science*, *182*(4), 679-685. <https://doi.org/doi:10.1007/s11845-013-0955-5>

O'Connor, M. I., Teo, W. Z. W., Brenner, L. H., & Bal, B. S. (2019). Medicolegal sidebar: Avoiding gender-based inequities during orthopaedic training. *Clinical Orthopaedics and Related Research*, *477*(6), 1284-1287. <https://doi.org/doi:10.1097/CORR.0000000000000786>

O'Neill, S., Foster, K., & Gilbert-Obrart, A. (2016). The Balint group experience for medical students: A pilot project. *Psychoanalytic Psychotherapy*, *30*(1), 96-108. <https://doi.org/doi:10.1080/02668734.2015.1107124>

O'Reilly, C. L., Bell, J. S., & Chen, T. F. (2010). Consumer-led mental health education for pharmacy students. *American Journal of Pharmaceutical Education*, *74*(9), 167. <https://doi.org/doi:10.5688/aj7409167>

Ogilvie, L., Larkin, T. J., & Keller-Unger, J. L. (2021). Unconscious bias and self-care: Key components of an integrated, comprehensive health care system nurse manager leadership development program. *Nurse Leader*, *19*(3), 250-254. <https://doi.org/10.1016/j.mnl.2021.02.012>

Okorie-Awé, C., Crawford, S. Y., Sharp, L. K., Jaki, B. U., & Kachlic, M. D. (2021). A faculty and staff workshop on microaggression and implicit bias: Knowledge and awareness of student, faculty, and staff experiences. *Currents in Pharmacy Teaching and Learning*, *13*(9), 1200-1209. <https://doi.org/doi:10.1016/j.cptl.2021.06.031>

Okubanjo, O., & Lovell, E. (2017). Healthcare disparities. *Western Journal of Emergency Medicine*, *18*, S51.

Oliver, T. L., Shenkman, R., Diewald, L. K., & Smeltzer, S. C. (2021). Reflective journaling of nursing students on weight bias. *Nurse Education Today*, *98*, 104702. <https://doi.org/doi:10.1016/j.nedt.2020.104702>

Olveczky, D. D., Chatter-Jee, A., oval, R., Afolabi, T., & Said, J. (2019). Building a toolkit for medical students: Addressing microaggressions & discrimination on the wards. *Journal of General Internal Medicine*, *34*(2), S792.

Omori, A., Tateno, A., Ideno, T., Takahashi, H., Kawashima, Y., Takemura, K., & Okubo, Y. (2012). Influence of contact with schizophrenia on implicit attitudes towards schizophrenia patients held by clinical residents. *BMC Psychiatry*, *12*. <https://doi.org/doi:10.1186/1471-244X-12-205>

Owen, J., Drinane, J. M., Tao, K. W., DasGupta, D. R., Zhang, Y. S. D., & Adelson, J. (2018). An experimental test of microaggression detection in psychotherapy: Therapist multicultural orientation. *Professional Psychology: Research and Practice*, *49*(1), 9-21. <https://doi.org/doi:10.1037/pro0000152>

Ozcan Edeer, A., & Rust, N. (2022). Effectiveness of interprofessional education modules on cultural competency of physical therapy and occupational therapy students. *Internet Journal of Allied Health Sciences & Practice*, *20*(2), 1-8. <https://doi.org/doi:10.46743/1540-580X/2022.2183>

Padilla, L., & Garcia, D. T. (2022). Introductory curriculum to health disparities for medical physics trainees: A pilot experience. *International Journal of Radiation Oncology Biology Physics*, *114*, e21-e22. <https://doi.org/doi:10.1016/j.ijrobp.2022.06.046>

Pai, H.-C., Wu, P.-L., Hsu, W.-Y., Hung, C.-A., Liu, N.-Y., & Yen, W.-J. (2021). The effect of a situation model nursing education action program on gender-bias awareness and gender-friendliness barriers in novice nursing students. *Nurse Education in Practice*, *54*, 103129. <https://doi.org/doi:10.1016/j.nepr.2021.103129>

Paniagua, M., Waldron, M., & Eppensteiner, J. C. (2010). Preemptive exposure to geriatrics: An undergraduate chronic care internship. *Journal of the American Geriatrics Society*, *58*, S95.

Papish, A., Kassam, A., Modgill, G., Vaz, G., Zanussi, L., & Patten, S. (2013). Reducing the stigma of mental illness in undergraduate medical education: a randomized controlled trial. *BMC Medical Education*, *13*, 141. <https://doi.org/doi:10.1186/1472-6920-13-141>

Paroz, S., Bonvin, R., Casillas, A., ra, Vadot, S., Viret, F., Daele, A., & Bodenmann, P. (2014). Cultural competence education in a simulated clinical environment: A pilot experience. *Journal of General Internal Medicine*, *29*, S513-S514.

Paroz, S., Dory, E., Vu, F., Bodenmann, P., Casillas, A., & ra. (2014). Building the diversity bridge abroad: The strategy to implement pre-graduate cultural competency medical education in Lausanne, Switzerland. *Journal of General Internal Medicine*, *29*, S509.

Patel, K., Ansari, S., Hexom, B., & Dissanayake, V. (2021). 187 An assessment of a diversity, inclusion, and racial equity curriculum in emergency medicine residency training. *Annals of Emergency Medicine*, *78*, S75. <https://doi.org/doi:10.1016/j.annemergmed.2021.09.198>

Patrick, S., Connolly, C. M., Pope, M., Pangelinan, J. S., & Coker, A. D. (2011). *The token activity*. In Pope, M., Pangelinan, J.S., & Coker A.D. (Eds.), *Experiential activities for teaching multicultural competence in counseling*. Alexandria, VA: American Counseling Association. Retrieved from <https://www.counseling.org/publications/frontmatter/72904-fm.pdf>

Paul, T. J., Mitchell, A., Lagrenade, J., McCaw-Binns, A., Falloon, D., & Williams-Green, P. (2006). More questions than answers? Expanding students' reflections from a community health experience. *Education for Health: Change in Learning & Practice*, *19*(2), 244-250. <https://doi.org/doi:10.1080/13576280600783760>

Paulsen, E. L., Wells, K. M., Ajagbe, O., & Ervoes, J. (2019). 93. Teaching residents to mitigate prejudice. *Academic Pediatrics*, *19*(6), e41-e42. <https://doi.org/10.1016/j.acap.2019.05.107>

Pearce, L. (2017). Making nurse education LGBT-friendly. *Nursing Standard*, *31*(23), 22-24. <https://doi.org/doi:10.7748/ns.31.23.22.s23>

Pearson, Q. M. Breaking the silence in the counselor education classroom: A training seminar on counseling sexual minority clients. *Journal of Counseling & Development*, *81*(3), 292-300. <https://doi.org/doi:10.1002/j.1556-6678.2003.tb00256.x>

Pekcetin, S., Hasgul, E., Yildirim Dugeroglu, R., & Arabaci, Z. (2021). The effect of extended contact with community-dwelling older adults on the ageist and helping attitudes of home care students: A single-blind randomised controlled trial. *International Journal of Older People Nursing*, *16*(5), e12382. <https://doi.org/doi:10.1111/opn.12382>

Pena Dolhun, E., Munoz, C., & Grumbach, K. (2003). Cross-cultural education in U.S. medical schools: development of an assessment tool. *Academic Medicine: Journal of the Association of American Medical Colleges*, *78*(6), 615-622. <https://doi.org/doi:10.1097/00001888-200306000-00012>

Peralta, J. B., Smith, D. F., Duh-Leong, C., Durstenfeld, A., & Acholonu, R. G. (2018). Impact of social determinants of health curriculum on resident empathy. *Academic Pediatrics*, *18*(5), e2. <https://doi.org/doi:10.1016/j.acap.2018.04.017>

Perry, L. C. (2012). The effects of participation in a human sexuality workshop on the attitudes of counselors in training toward homosexuality [ProQuest Information & Learning]. *Dissertation Abstracts International: Section B: The Sciences and Engineering, 73*(3B), 1879. Retrieved from <http://proxy.library.vcu.edu/login?url=https://search.ebscohost.com/login.aspx?direct=true&AuthType=ip,url,cookie,uid&db=psyh&AN=2012-99180-117&site=ehost-live&scope=site>

Person, H., & Bucuvalas, J. (2021). Developing and implementing a behavior-oriented anti-racism curriculum for pediatric liver transplant teams. *Journal of Pediatric Gastroenterology and Nutrition*, *73*, S315-S316.

Pham, V., Murray, J., & Pasha, J. (2019). Unlocking implicit bias: Implementation of an implicit bias workshop to increase resident physician awareness of personal implicit bias and its effect on patient care. *Journal of General Internal Medicine*, *34*(2), S394.

Phelan, S. M., Burke, S. E., Hardeman, R. R., White, R. O., Przedworski, J., Dovidio, J. F., Perry, S. P., Plankey, M., A. Cunningham, B., Finstad, D., W. Yeazel, M., & van Ryn, M. (2017). Medical school factors associated with changes in implicit and explicit bias against gay and lesbian people among 3492 graduating medical students. *Journal of General Internal Medicine*, *32*(11), 1193-1201. <https://doi.org/doi:10.1007/s11606-017-4127-6>

Phelan, S. M., Puhl, R. M., Burke, S. E., Hardeman, R., Dovidio, J. F., Nelson, D. B., Przedworski, J., Burgess, D. J., Perry, S., Yeazel, M. W., & van Ryn, M. (2015). The mixed impact of medical school on medical students' implicit and explicit weight bias. *Medical Education*, *49*(10), 983-992. <https://doi.org/doi:10.1111/medu.12770>

Pieterse, A. L. (2009). Teaching antiracism in counselor training: Reflections on a course. *Journal of Multicultural Counseling and Development*, *37*(3), 141-152. <https://doi.org/doi:10.1002/j.2161-1912.2009.tb00098.x>

Pisal, H., Sutar, S., Sastry, J., Kapadia-Kundu, N., Joshi, A., Joshi, M., Leslie, J., Scotti, L., Bharucha, K., Suryavanshi, N., Phadke, M., Bollinger, R., & Shankar, A. V. (2007). Nurses' health education program in India increases HIV knowledge and reduces fear. *JANAC: Journal of the Association of Nurses in AIDS Care*, *18*(6), 32-43. <https://doi.org/doi:10.1016/j.jana.2007.06.002>

Pittman, J. O. E., Noh, S., & Coleman, D. (2010). Evaluating the effectiveness of a consumer delivered anti-stigma program: Replication with graduate-level helping professionals. *Psychiatric Rehabilitation Journal*, *33*(3), 236-238. <https://doi.org/doi:10.2975/33.3.2010.236.238>

Poirier, T. I., Butler, L. M., Devraj, R., Gupchup, G. V., Santanello, C., & Lynch, J. C. (2009). A cultural competency course for pharmacy students. *American Journal of Pharmaceutical Education*, *73*(5), 81. <https://doi.org/doi:10.5688/aj730581>

Pomarede, M. J. M., & Capara, N. R. (2007). [Cultural diversity reflexive learning]. *El aprendizaje reflexivo de la diversidad cultural*, *30*(10), 59-64.

Poustchi, Y., Saks, N. S., Piasecki, A. K., Hahn, K. A., & Ferrante, J. M. (2013). Brief intervention effective in reducing weight bias in medical students. *Family Medicine*, *45*(5), 345-348.

Power, T., Virdun, C., Sherwood, J., Parker, N., Van Balen, J., Gray, J., & Jackson, D. (2016). REM: A Collaborative Framework for Building Indigenous Cultural Competence. *Journal of Transcultural Nursing*, *27*(5), 439-446. <https://doi.org/doi:10.1177/1043659615587589>

Pribbenow, C. M., Caldwell, K. E. H., Dantzler, D. D., Brown, P., Jr., & Carnes, M. (2021). Decreasing racial bias through a facilitated game and workshop: The case of fair play. *Simulation and Gaming*, *52*(3), 386-402. <https://doi.org/doi:10.1177/1046878120983384>

Price, M., Mahle, R., Tarnasky, A., Dalal, N., Wachsmuth, L., Godwin, C., Webb, J., & Brown, J. T. (2020). The student navigators project: Improving access to early education in end-of-life care for healthcare professional students (FR441A). *Journal of Pain and Symptom Management*, *59*(2), 469-470. <https://doi.org/doi:10.1016/j.jpainsymman.2019.12.153>

Price, S. C. (2022). Enhancing nursing faculty awareness of implicit racial bias, in an online teaching environment, as an antiracism strategy toward the elimination of health disparity for the Black, Hispanic, and American Indian populations [ProQuest Information & Learning]. *Dissertation Abstracts International: Section B: The Sciences and Engineering, 83*(11B). Retrieved from <http://proxy.library.vcu.edu/login?url=https://search.ebscohost.com/login.aspx?direct=true&AuthType=ip,url,cookie,uid&db=psyh&AN=2022-70649-003&site=ehost-live&scope=site>

Pryce, P. A., Uwemedimo, O., Goenka, P., & Barone, S. (2019). 86. Early impact of a health equity, diversity, and inclusion curricula on resident knowledge, attitudes and skill in cross-cultural care. *Academic Pediatrics*, *19*(6), e39. <https://doi.org/10.1016/j.acap.2019.05.100>

Rahman, F., Tiako, M. J. N., Sabin, J., Boatright, D., Black, A., & Genao, I. (2020). Measuring the difference in response to online structural competency training between medical residents and public health students. *Journal of General Internal Medicine*, *35*, S750.

Ramsdell, A. (2019). Development of a medical student clinical elective focusing on health equity: "Exploring social determinants of health at an urban community hospital." A look at impacts on student attitudes and readiness to serve a diverse patient population. *Journal of General Internal Medicine*, *34*(2), S799.

Rastegar, D. A., Fingerhood, M. I., & Jasinski, D. R. (2004). A resident clerkship that combines inpatient and outpatient training in substance abuse and HIV care. *Substance Abuse*, *25*(4), 11-15. <https://doi.org/doi:10.1300/j465v25n04_02>

Rathod, S., Kingdon, D., Phiri, P., & Gobbi, M. (2010). Developing culturally sensitive cognitive behaviour therapy for psychosis for ethnic minority patients by exploration and incorporation of service users' and health professionals' views and opinions. *Behavioural and Cognitive Psychotherapy*, *38*(5), 511-533. <https://doi.org/doi:10.1017/S1352465810000378>

Redmond, N., Safford, M. M., Waugh, J., & Ring, J. M. (2016). University of Alabama school of medicine (UASOM) faculty scholars in health disparities and culturally responsive care. *Journal of General Internal Medicine*, *31*(2), S852-S853.

Reed, O. M. (2021). Evaluating the effectiveness of a transgender-affirmative care training on healthcare workers’ and trainees’ attitudes toward and knowledge of routine care and transition support for transgender individuals [ProQuest Information & Learning]. *Dissertation Abstracts International: Section B: The Sciences and Engineering, 82*(6B). Retrieved from <http://proxy.library.vcu.edu/login?url=https://search.ebscohost.com/login.aspx?direct=true&AuthType=ip,url,cookie,uid&db=psyh&AN=2021-08068-098&site=ehost-live&scope=site>

Reilly, J. B., Ogdie, A. R., Von Feldt, J. M., & Myers, J. S. (2013). Teaching about how doctors think: a longitudinal curriculum in cognitive bias and diagnostic error for residents. *BMJ Quality & Safety*, *22*(12), 1044-1050. <https://doi.org/doi:10.1136/bmjqs-2013-001987>

Reliford, A., Berry, O. O., Burgos, J. J., & Liaw, K. R.-L. (2022). Holding space for facilitated dialogues on antiracism in academic medicine. *Journal of the American Academy of Child & Adolescent Psychiatry*, *61*(8), 953-956. <https://doi.org/doi:10.1016/j.jaac.2022.03.020>

Reliford, A. O., Ron-Li Liaw, K., Berry, O. O., & Burgos, J. J. (2021). Creating and implementing facilitated dialogs on antiracism in psychiatry, child psychiatry, and academic medicine. *Journal of the American Academy of Child and Adolescent Psychiatry*, *60*, S322-S323. <https://doi.org/doi:10.1016/j.jaac.2021.07.786>

Ries, E. (2019). Battling bias’s distorted images: Looking in the mirror is the first step. *PT in Motion*, *11*(9), 18-27.

Rimmer, A. (2016). Avoiding unconscious bias. *BMJ (Online)*, *354*, 1308. <https://doi.org/doi:10.1136/bmj.i4366>

Roberts, D. H., Kane, E. M., Jones, D. B., Almeida, J. M., Bell, S. K., Weinstein, A. R., & Schwartzstein, R. M. (2011). Teaching medical students about obesity: a pilot program to address an unmet need through longitudinal relationships with bariatric surgery patients. *Surgical Innovation*, *18*(2), 176–183. <https://doi.org/10.1177/1553350611399298>

Roberts, E., Richeson, N., Thornhill, J. T., Corwin, S. J., & Eleazer, G. P. (2006). The senior mentor program at the University of South Carolina School of Medicine: An innovative geriatric longitudinal curriculum. *Gerontology & Geriatrics Education*, *27*(2), 11-23. <https://doi.org/doi:10.1300/J021v27n02_03>

Roberts, L. M., Wiskin, C., & Roalfe, A. (2008). Effects of exposure to mental illness in role-play on

undergraduate student attitudes. *Family Medicine*, *40*(7), 477-483.

Roberts, L. W. (2021). Advancing equity in academic medicine. *Academic Medicine: Journal of the Association of American Medical Colleges*, *96*(6), 771-772. <https://doi.org/doi:10.1097/ACM.0000000000004068>

Roberts, R. M., & Aida Farhana, H. S. (2010). Effectiveness of a first aid information video in reducing epilepsy-related stigma. *Epilepsy and Behavior*, *18*(4), 474-480. <https://doi.org/doi:10.1016/j.yebeh.2010.06.004>

Rodriguez, N., Kintzer, E., List, J., Lypson, M., Grochowalski, J. H., Marantz, P. R., & Gonzalez, C. M. (2021). Implicit bias recognition and management: Tailored instruction for faculty. *Journal of the National Medical Association*, *113*(5), 566-575. <https://doi.org/doi:10.1016/j.jnma.2021.05.003>

Rogovin, T. (2017). Changing implicit and explicit attitudes toward addiction in students in a Substance Abuse Counseling Psychology Masters program: A longitudinal academic review [ProQuest Information & Learning]. *Dissertation Abstracts International: Section B: The Sciences and Engineering, 78*(6B). Retrieved from <http://proxy.library.vcu.edu/login?url=https://search.ebscohost.com/login.aspx?direct=true&AuthType=ip,url,cookie,uid&db=psyh&AN=2017-16345-144&site=ehost-live&scope=site>

Ross, P. T., & Lypson, M. L. (2014). Using artistic-narrative to stimulate reflection on physician bias. *Teaching and Learning in Medicine*, *26*(4), 344-349. <https://doi.org/doi:10.1080/10401334.2014.945032>

Roswell, R. O., Cogburn, C. D., Tocco, J., Martinez, J., Bangeranye, C., Bailenson, J. N., Wright, M., Mieres, J. H., & Smith, L. (2020). Cultivating empathy through virtual reality: Advancing conversations about racism, inequity, and climate in medicine. *Academic Medicine*, 1882-1886. <https://doi.org/doi:10.1097/ACM.0000000000003615>

Rothman, T., Malott, K. M., & Paone, T. R. (2012). Experiences of a course on the culture of Whiteness in counselor education. *Journal of Multicultural Counseling and Development*, *40*(1), 37-48. <https://doi.org/doi:10.1111/j.2161-1912.2012.00004.x>

Ruiz, M. S. (2011). Evaluation of cultural competency medical education curriculum through the assessment of changes in student racial attitudes [ProQuest Information & Learning]. *Dissertation Abstracts International Section A: Humanities and Social Sciences, 72*(6A), 1933. Retrieved from <http://proxy.library.vcu.edu/login?url=https://search.ebscohost.com/login.aspx?direct=true&AuthType=ip,url,cookie,uid&db=psyh&AN=2011-99231-015&site=ehost-live&scope=site>

Sabin, J., Calista, J., Dykhouse, E., Eisdorfer, E., Foiles, A., Puerto, G., Terrien, J., Yazdani, M., Garcia, M., Hale, J., Rappaport, L., Valdman, O., & Tjia, J. (2020). Minimizing defensiveness in clinician education about implicit bias: Lessons learned from a community-engaged randomized clinical trial. *Health Services Research*, *55*, 51-52. <https://doi.org/doi:10.1111/1475-6773.13399>

Sabin, J., Van Schaik, E., Lynch, E., & Stoner, S. (2010). Does awareness of unconscious associations enhance learning about healthcare disparities? *American Journal of Epidemiology*, *171*, S129.

Sadau, E. W., & Capeles, T. (2019). The butterfly effect in healthcare: What happens when an organization tackles unconscious bias and promotes diversity of thought? *Journal of Healthcare Management*, *64*(5), 265-271. <https://doi.org/doi:10.1097/JHM-D-19-00152>

Saetermoe, C. L., Chavira, G., Khachikian, C. S., Boyns, D., & Cabello, B. (2017). Critical race theory as a bridge in science training: The California State University, Northridge BUILD PODER program. *BMC Proceedings*, *11*. <https://doi.org/doi:10.1186/s12919-017-0089-2>

Safdieh, J. E., Govindarajan, R., Gelb, D. J., Odia, Y., & Soni, M. (2019). Core curriculum guidelines for a required clinical neurology experience. *Neurology*, *92*(13), 619-626. <https://doi.org/doi:10.1212/WNL.0000000000007187>

Salahou, A., Rahmon, D., & Fedorowicz, M. (2021). Medical students confront racism and systemic oppression amidst a global pandemic. *Academic Medicine: Journal of the Association of American Medical Colleges*, *96*(5), e18. <https://doi.org/doi:10.1097/ACM.0000000000003966>

Samberg, D., Spinella, S., & Childers, J. (2021). Impact of AWEB-based curriculum on use of stigmatizing language for SUD. *Journal of Addiction Medicine*, *15*, E27. <https://doi.org/doi:10.1097/ADM.0000000000000902>

Samberg, D., Spinella, S., Rothenberger, S., & Childers, J. (2021). Impact of a web-based curriculum on internal medicine resident use of stigmatizing language for substance use disorder. *Journal of General Internal Medicine*, *36*, S467.

Sanchez, S., & Aysola, J. (2018). Am I biased? Using the Implicit Association Test to start the conversation among internal medicine residents. *Journal of General Internal Medicine*, *33*(2), 683-684.

Sandoval, R., Afolabi, T., Said, J., Dunleavy, S., Chatterjee, A., & Ölveczky, D. (2020). Building a tool kit for medical and dental students: Addressing microaggressions and discrimination on the wards. *MedEdPORTAL: The Journal of Teaching and Learning Resources*, *16*, 10893. <https://doi.org/doi:10.15766/mep_2374-8265.10893>

Sandoval, R., Afolabi, T., Said, J., Connor, J., Hossain, A., Kassamali, B., Srinivasan, M., Dunleavy, S., Cheng, A., Olveczky, D., & Chatterjee, A. (2021). Addressing microaggressions and discrimination in the clinical environment during medical school: A multi-year educational intervention. *Journal of General Internal Medicine*, *36*, S97-S98.

Sangganjanavanich, V. F., Pope, M., Pangelinan, J. S., & Coker, A. D. (2011). Confronting prejudice and stereotypes: Working with transgender individuals. In Pope, M., Pangelinan, J.S., & Coker A.D. (Eds.), *Experiential activities for teaching multicultural competence in counseling*. Alexandria, VA: American Counseling Association. Retrieved from <https://www.counseling.org/publications/frontmatter/72904-fm.pdf>

Sarabia-Cobo, C. M., & Castanedo Pfeiffer, C. (2015). Changing negative stereotypes regarding aging in undergraduate nursing students. *Nurse Education Today*, *35*(9), e60-64. <https://doi.org/doi:10.1016/j.nedt.2015.06.006>

Sasaki, H. M. (2008). An intervention for stereotype automaticity in therapist-trainees: A pilot study in implicit multicultural social cognition [ProQuest Information & Learning]. *Dissertation Abstracts International: Section B: The Sciences and Engineering, 69*(6B), 3861. Retrieved from <http://proxy.library.vcu.edu/login?url=https://search.ebscohost.com/login.aspx?direct=true&AuthType=ip,url,cookie,uid&db=psyh&AN=2008-99240-290&site=ehost-live&scope=site>

Schaeffer, S., & Lupton, K. (2017). Enhancing diversity in internal medicine (IM) training: Implicit bias training for residency admissions committees. *Journal of General Internal Medicine*, *32*(2), S674.

Schaeffer, S., Narayana, S., & Eniasivam, A. (2020). Embracing our values: Creating a social medicine core within academic hospital medicine. *Journal of General Internal Medicine*, *35*, S647-S648.

Schenner, M., Kohlbauer, D., & Günther, V. (2011). Kommunizieren statt stigmatisieren - Verändert der soziale kontakt mit einer an depression erkrankten person die einstellungen von medizinstudentInnen zu psychisch kranken? Eine studie zu einstellungen von medizinstudentInnen zu psychisch erkrankten menschen [Communicate instead of stigmatizing - does social contact with a depressed person change attitudes of medical students towards psychiatric disorders? A study of attitudes of medical students to psychiatric patients]. *Neuropsychiatrie: Klinik, Diagnostik, Therapie und Rehabilitation : Organ der Gesellschaft Osterreichischer Nervenarzte und Psychiater*, *25*(4), 199–207.

Schim, S. M., Doorenbos, A. Z., & Borse, N. N. (2006). Enhancing cultural competence among hospice staff. *The American Journal of Hospice & Palliative Care*, *23*(5), 404-411. <https://doi.org/doi:10.1177/1049909106292246>

Schmetzer, A. D., Lafuze, J. E., & Jack, M. E. (2008). Overcoming stigma: involving families in medical student and psychiatric residency education. *Academic Psychiatry: The Journal of the American Association of Directors of Psychiatric Residency Training and the Association for Academic Psychiatry*, *32*(2), 127-131. <https://doi.org/doi:10.1176/appi.ap.32.2.127>

Schneider, P., Dixon-Shambley, K., Marcotte, M. P., Weber, Z. A., Menegay, M., Quarshie, E., Afflitto, S., Jenkins, K., Oza-Frank, R., Lorenz, A., & Lappen, J. (2022). Assessing impact of implicit bias/anti-racism programs for obstetrical providers in a statewide quality improvement initiative. *American Journal of Obstetrics and Gynecology*, *226*, S618-S619. <https://doi.org/doi:10.1016/j.ajog.2021.11.1019>

Schwartz, B. D., Horst, A., Fisher, J. A., Michels, N., & Van Winkle, L. J. (2020). Fostering empathy, implicit bias mitigation, and compassionate behavior in a medical humanities course. *International Journal of Environmental Research and Public Health*, *17*(7). <https://doi.org/doi:10.3390/ijerph17072169>

Scott, C. J., Martin, M., & Hamilton, G. (2003). Training of medical professionals and the delivery of health care as related to cultural identity groups. *Academic Emergency Medicine*, *10*(11), 1149-1152. <https://doi.org/doi:10.1111/j.1553-2712.2003.tb00595.x>

Selig, S., Tropiano, E., & Greene-Moton, E. (2006). Teaching cultural competence to reduce health disparities. *Health Promotion Practice*, *7*(3), 247S-255S. <https://doi.org/doi:10.1177/1524839906288697>

Serra Galceran, M., Mestres Camps, L., González Soriano, M., Leyva Moral, J. M., De Dios Sánchez, R., & Montiel Pastor, M. (2013). Competencia clínico cultural: Análisis de la capacitación de los profesionales de la salud. *Index de Enfermería*, *22*(1), 16-19. <https://doi.org/doi:10.4321/S1132-12962013000100004>

Shapiro, J., Lie, D., Gutierrez, D., & Zhuang, G. (2006). "That never would have occurred to me”: A qualitative study of medical students' views of a cultural competence curriculum. *BMC Medical Education*, *6*, 31. <https://doi.org/doi:10.1186/1472-6920-6-31>

Sharon, E., Emmerich, A., & Parekh, R. (2013). Diversity dialogue: an innovative model for diversity training. In Parekh (Ed.), *The Massachusetts General Hospital textbook on diversity and cultural sensitivity in mental health* (pp. 191-212). New York, NY: Springer New York. Retrieved from <https://link.springer.com/chapter/10.1007/978-1-4614-8918-4_8>

Shen, Y., Dong, H., Fan, X., Zhang, Z., Li, L., Lv, H., Xue, Z., & Guo, X. (2014). What can the medical education do for eliminating stigma and discrimination associated with mental illness among future doctors? effect of clerkship training on Chinese students' attitudes. *International Journal of Psychiatry in Medicine*, *47*(3), 241-254. <https://doi.org/doi:10.2190/PM.47.3.e>

Shteinlukht, T., Eyler, L. T., Dev, S. I., & Dowling, N. M. (2016). Dealing with the unseen: Assessing and addressing implicit attitudes to enhance professional success in geriatric mental healthcare. *American Journal of Geriatric Psychiatry*, *24*(3), S18-S19. <https://doi.org/doi:10.1016/j.jagp.2016.01.027>

Shteinlukht, T., Eyler, L. T., Joo, J. H., & Ali, A. A. (2016). Diversity in action: Assessing and addressing biases in the workplace and patient care. *American Journal of Geriatric Psychiatry*, *24*(3), S3-S4. <https://doi.org/10.1016/j.jagp.2016.01.003>

Shultz, C., & Skorcz, S. (2012). African American infant mortality and the Genesee County, MI REACH 2010 initiative: An evaluation of the Undoing Racism Workshop. *Social Work in Public Health*, *27*(6), 567-603. <https://doi.org/doi:10.1080/19371910903253236>

Shutak, C. W. (2017). An academic half-day for healthcare disparities and social justice. *Academic Pediatrics*, *17*(5), e20. <https://doi.org/doi:10.1016/j.acap.2017.04.072>

Siegelman, J., Woods, C., Oyewo, A., Salhi, B., Bryant, A., & Heron, S. (2015). Disparities in pain management: An educational intervention using the implicit association test. *Western Journal of Emergency Medicine*, *16*, S52.

Siem, B., Neymeyer, L., & Rohmann, A. (2021). Entertainment education as a means to reduce anti-Muslim prejudice — For whom does it work best? An extended replication of Murrar and Brauer (2018). *Social Psychology*, *52*(1), 51-60. <https://doi.org/doi:10.1027/1864-9335/a000432>

Sikora, S. A. (2006). The University of Arizona College of Medicine Optimal Aging Program: Stepping in the shadows of successful aging. *Gerontology & Geriatrics Education*, *27*(2), 59-68. <https://doi.org/doi:10.1300/J021v27n02_07>

Simpson, T., Evans, J., Goepfert, A., & Elopre, L. (2022). Implementing a graduate medical education anti-racism workshop at an academic university in the Southern USA. *Medical Education Online*, *27*(1). <https://doi.org/doi:10.1080/10872981.2021.1981803>

Singaravelu, H. D., Pope, M., Pangelinan, J. S., & Coker, A. D. (2011). Stereotypes and generalizations. In Pope, M., Pangelinan, J.S., & Coker A.D. (Eds.), *Experiential activities for teaching multicultural competence in counseling*. Alexandria, VA: American Counseling Association. Retrieved from <https://www.counseling.org/publications/frontmatter/72904-fm.pdf>

Singer, R., Beth, i., Crane, B., Lemay Jr, E. P., & Omary, S. (2019). Improving the knowledge, attitudes, and behavioral intentions of perinatal care providers toward childbearing individuals identifying as LGBTQ: A quasi-experimental study. *Journal of Continuing Education in Nursing*, *50*(7), 303-312. <https://doi.org/doi:10.3928/00220124-20190612-05>

Singh, S. (2019). What do we know the experiences and outcomes of anti-racist social work education? An empirical case study evidencing contested engagement and transformative learning. *Social Work Education*, *38*(5), 631-653. <https://doi.org/doi:10.1080/02615479.2019.1592148>

Sir, O., & Schoon, Y. (2017). Education of emergency physicians in geriatrics improve knowledge and skills towards older patients admitted to the emergency department. *European Geriatric Medicine*, *8*, S149-S150.

Sirotin, N., Pho, A., & Boutin-Foster, C. (2013). Global health at home: Development of the vulnerable and immigrant populations (VIP) program for medical residents. *Journal of General Internal Medicine*, *28*, S465.

Smith, L., Proctor, G., & Akondo, D. (2021). Confronting racism in counselling and therapy training—Three experiences of a seminar on racism and whiteness. *Psychotherapy & Politics International*, *19*(2), 1-11. <https://doi.org/doi:10.1002/ppi.1579>

Snyder-Roche, S., Pope, M., Pangelinan, J. S., & Coker, A. D. (2011). Take a walk in my shoes. In Pope, M., Pangelinan, J.S., & Coker A.D. (Eds.), *Experiential activities for teaching multicultural competence in counseling*. Alexandria, VA: American Counseling Association. Retrieved from <https://www.counseling.org/publications/frontmatter/72904-fm.pdf>

Sotsky, J., & Worthington, C. (2021). A workshop on interrupting microaggressions for a surgical service: An opportunity for consultation-liaison psychiatry to address bias and inequity. *Journal of the Academy of Consultation-Liaison Psychiatry*, *62*(3), 363-367. <https://doi.org/doi:10.1016/j.jaclp.2021.02.001>

Sotto-Santiago, S., Mac, J., Duncan, F., & Smith, J. (2020). "I didn't know what to say": Responding to racism, discrimination, and microaggressions with the OWTFD approach. *MedEdPORTAL: The Journal of Teaching and Learning Resources*, *16*, 10971. <https://doi.org/doi:10.15766/mep_2374-8265.10971>

Sowers, L. (2019). Battling blind spots: Radiology leaders are first movers in Vanderbilt's training efforts to recognize and mitigate unconscious bias. *ACR Bulletin*, 18-19.

Spieß, E., Kaminski, S., & Dukat, U. (2006). Sensibilisierung gegenüber fremdenfeindlichkeit und rassismus--Ergebnisse einer wissenschaftlichen begleitung des XENOS-projektes 'Konzeptwerkstatt gegen Rechts' [Sensitization and social psychological constructs, the Deutsche Angestellten-Akademie Sudbayern's (The German Employee Academy of Southern Bavaria) XENOS project]. *Gruppendynamik und Organisationsberatung*, *37*(4), 347-362. <https://doi.org/doi:10.1007/s11612-006-0149-y>

Steed, R. (2010). Cultural competency instruction in a 3d virtual world [ProQuest Information & Learning]. *Dissertation Abstracts International Section A: Humanities and Social Sciences, 70*(11A), 4257. Retrieved from <http://proxy.library.vcu.edu/login?url=https://search.ebscohost.com/login.aspx?direct=true&AuthType=ip,url,cookie,uid&db=psyh&AN=2010-99090-024&site=ehost-live&scope=site>

Stein, J. N., Gaddy, J. J., Sorah, J. D., Wooten, M., Gibson, K., & Collichio, F. A. (2022). Seeking racial equity in hematology and oncology: A fellow-led educational series to promote reflection and action. *Journal of Clinical Oncology. Conference: Annual Meeting of the American Society of Clinical Oncology, ASCO*, *40*(16). <https://doi.org/doi:10.1200/JCO.2022.40.16_suppl.11004>

Steinfeldt, J. A., & Steinfeldt, M. C. (2012). Multicultural training intervention to address American Indian stereotypes. *Counselor Education and Supervision*, *51*(1), 17-32. <https://doi.org/doi:10.1002/j.1556-6978.2012.00002.x>

Steinfeldt, J. A., & Wong, Y. J. (2010). Multicultural training on American Indian issues: Testing the effectiveness of an intervention to change attitudes toward native-themed mascots. *Cultural Diversity and Ethnic Minority Psychology*, *16*(2), 110-115. <https://doi.org/doi:10.1037/a0018633>

Stephenson, R., Lesco, G., Babii, V., Luchian, A., Bakunina, N., De Vasconcelos, A. S., Blondeel, K., Cáceres, C. F., Pitter, R. A., Metheny, N., Goldenberg, T., Kiarie, J., & Toskin, I. (2021). Provider attitudes towards a brief behavioral intervention for sexual health in Moldova. *BMC Public Health*, *21*(1). <https://doi.org/doi:10.1186/s12889-021-11490-5>

Stevenson, S. (2018). The group as a psycho-educational medium for the teaching of anti-racist practice on social work trainings. *Journal of Social Work Practice*, *32*(3), 337-349. <https://doi.org/doi:10.1080/02650533.2017.1359779>

Steward, A., Hasche, L., Talamantes, M., & Bernhardt, M. (2021). The impact of intergenerational engagement on social work students' attitudes toward aging: The example of Clermont College. *Health & Social Work*, *45*(4), 250–258. <https://doi.org/10.1093/hsw/hlaa023>

Stewart, R., & Whiteman, L. (2013). Diminishing bias in sickle cell disease. *Medical Teacher*, *35*(6), 522-522. <https://doi.org/doi:10.3109/0142159X.2013.772967>

Stewart, T. L., Latu, I. M., Martin, T., Walsh, S. P., Schmidt, A., & Kawakami, K. (2022). Implicit bias reduction that lasts: Putting situational attribution training to the test. *Journal of Applied Social Psychology* *52*(11), 1062-1069. <https://doi.org/doi:10.1111/jasp.12912>

Stone, J., & Moskowitz, G. B. (2011). Non-conscious bias in medical decision making: What can be done to reduce it? *Medical Education*, *45*(8), 768-776. <https://doi.org/doi:10.1111/j.1365-2923.2011.04026.x>

Strewler, A., Bauer, S., Pearson, M., Wlodarczyk, S., Duggal, P., & Shunk, R. L. (2021). Building capacity for addressing gender bias: A train-the-trainer program to promote gender equity. *Journal of General Internal Medicine*, *36*, S432-S433.

Strohbehn, G. W., Hoffman, S. J. K., Tokaz, M., Houchens, N., Slavin, R., Winter, S., Quinn, M., Ratz, D., Saint, S., Chopra, V., & Howell, J. D. (2020). Visual arts in the clinical clerkship: A pilot cluster-randomized, controlled trial. *BMC Medical Education*, *20*(1). <https://doi.org/doi:10.1186/s12909-020-02386-w>

Stuart, H., Chen, S.-P., Christie, R., Dobson, K., Kirsh, B., Knaak, S., Koller, M., Krupa, T., Lauria-Horner, B., Luong, D., Modgill, G., Patten, S. B., Pietrus, M., Szeto, A., & Whitley, R. (2014). Opening minds in Canada: Targeting change. *Canadian Journal of Psychiatry. Revue Canadienne de Psychiatrie*, *59*(10), S13-18. <https://doi.org/doi:10.1177/070674371405901s05>

Sun, M., Pu, W., Wang, Z., Hu, A., Yang, J., Chen, X., Fang, Y., Liu, Z., & Rosenheck, R. (2013). Investigation on the influence of a didactic course in psychiatry on attitudes of mental illness in Chinese college students. *Asia-Pacific Psychiatry: Official Journal of the Pacific Rim College of Psychiatrists*, *5*(3), 147-151. <https://doi.org/doi:10.1111/appy.12029>

Swiderski, D., Gonzalez, C. M., & Strelnick, A. H. (2011). Health disparities: A method to teach about values and assumptions. *Journal of General Internal Medicine*, *26*, S609-S610.

Symons, A. B., Morley, C. P., McGuigan, D., & Akl, E. A. (2014). A curriculum on care for people with disabilities: Effects on medical student self-reported attitudes and comfort level. *Disability and Health Journal*, *7*(1), 88-95. <https://doi.org/doi:10.1016/j.dhjo.2013.08.006>

Tajeu, G. S., Halanych, J., Juarez, L., Agne, A., Cherrington, A., Williams, J., Stepanikova, I., & Stone, J. (2021). Development of a multi component intervention to decrease racial bias among healthcare staff. *Journal of General Internal Medicine*, *36*, S129. <https://doi.org/doi:10.1007/s11606-022-07464-x>

Tate, E. V., & Prestidge, M. (2020). Evaluating the effectiveness of a structural competency and bias in medicine curriculum for internal medicine residents. *Journal of General Internal Medicine*, *35*, S725.

Taylor, J., Davis, L., Prudhomme, A., Mumphrey, C., & English, M. (2022). Assessing implicit bias in pediatric clerkships. *Journal of Investigative Medicine*, *70*, 661. <https://doi.org/doi:10.1136/jim-2022-SRMC.432>

Tedesco, L. A., & Albino, J. E. N. (2011). Women's health curriculum and cultural competence: An IWLC Working Group report. *Journal of Dental Education*, *75*(3), S28-30.

Tenorio da Silva, D., Pereira, A. M., de Oliveira Santos Silva, R., Menendez, A. S., Dos Santos, C., de Lima Florentino Junior, I., Felizardo Neves, S. J., Dosea, M. B., & Lyra, D. P., Jr. (2020). Using virtual patient software to improve pharmacy students' knowledge of and attitudes toward geriatric patients. *American Journal of Pharmaceutical Education*, *84*(5), 7230. <https://doi.org/doi:10.5688/ajpe7230>

Thackrah, R. D., Thompson, S., & C., r. (2013). Confronting uncomfortable truths: Receptivity and resistance to Aboriginal content in midwifery education. *Contemporary Nurse: A Journal for the Australian Nursing Profession*, *46*(1), 113-122. <https://doi.org/doi:10.5172/conu.2013.46.1.113>

Toporek, R. L., & Pope-Davis, D. B. (2005). Exploring the relationships between multicultural training, racial attitudes, and attributions of poverty among graduate counseling trainees. *Cultural Diversity and Ethnic Minority Psychology*, *11*(3), 259-271. <https://doi.org/doi:10.1037/1099-9809.11.3.259>

Torino, G. C., Donnelly, P. C., Pope, M., Pangelinan, J. S., & Coker, A. D. (2011). Racial-cultural dyadic role-play. In Pope, M., Pangelinan, J.S., & Coker A.D. (Eds.), *Experiential activities for teaching multicultural competence in counseling*. Alexandria, VA: American Counseling Association. Retrieved from <https://www.counseling.org/publications/frontmatter/72904-fm.pdf>

Tsai, J. W., & Michelson, C. D. (2017). Implicit bias training in pediatric residency: Attitudes amongst program directors and lessons learned from implementation. *Academic Pediatrics*, *17*(5), e53-e54. <https://doi.org/doi:10.1016/j.acap.2017.04.148>

Tsai, J. W., & Michelson, C. D. (2020). Attitudes toward implicit bias and implicit bias training among pediatric residency program directors: A national survey. *Journal of Pediatrics*, *221*, 4-6.e1. <https://doi.org/doi:10.1016/j.jpeds.2020.01.002>

Tuan, R., & Hsia, S. (2021). An integrated, longitudinal health equity curriculum to prepare socially responsible healthcare practitioners. *FASEB Journal. Conference: Experimental Biology, EB*, *35*. <https://doi.org/doi:10.1096/fasebj.2021.35.S1.03728>

Tucker, A., Liht, J., de Swardt, G., Arendse, C., McIntyre, J., & Struthers, H. (2016). Efficacy of tailored clinic trainings to improve knowledge of men who have sex with men health needs and reduce homoprejudicial attitudes in South Africa. *LGBT health*, *3*(6), 443-450. <https://doi.org/doi:10.1089/lgbt.2016.0055>

Uys, L., Chirwa, M., Kohi, T., Greeff, M., Naidoo, J., Makoae, L., Dlamini, P., Durrheim, K., Cuca, Y., & Holzemer, W. L. (2009). Evaluation of a health setting-based stigma intervention in five African countries. *AIDS Patient Care and STDs*, *23*(12), 1059-1066. <https://doi.org/doi:10.1089/apc.2009.0085>

Vaimberg, E., Demers, L., Ford, E., Sabatello, M., Stevens, B., & Dasgupta, S. (2021). Project inclusive genetics: Exploring the impact of patient-centered counseling training on physical disability bias in the prenatal setting. *PLoS ONE*, *16*(8), e0255722. <https://doi.org/doi:10.1371/journal.pone.0255722>

Van Bewer, V., Woodgate, R. L., Martin, D., & Deer, F. (2021). Exploring theatre of the oppressed and forum theatre as pedagogies in nursing education. *Nurse Education Today*, *103*, 104940. <https://doi.org/doi:10.1016/j.nedt.2021.104940>

van Ryn, M., Hardeman, R., Phelan, S. M., PhD, D. J. B., Dovidio, J. F., Herrin, J., Burke, S. E., Nelson, D. B., Perry, S., Yeazel, M., & Przedworski, J. M. (2015). Medical school experiences associated with change in implicit racial bias among 3547 students: A medical student CHANGES study report. *Journal of General Internal Medicine*, *30*(12), 1748-1756. <https://doi.org/doi:10.1007/s11606-015-3447-7>

Van Winkle, L. J., Schwartz, B. D., Horst, A., Fisher, J. A., Michels, N., & Thornock, B. O. (2021). Impact of a pandemic and remote learning on team development and elements of compassion in prospective medical students taking a medical humanities course. *International Journal of Environmental Research and Public Health*, *18*(9), 4856. <https://doi.org/doi:10.3390/ijerph18094856>

Varas-Diaz, N., Neil, s, T. B., Rodriguez-Madera, S. L., & Padilla, M. (2016). The role of emotions in the reduction of HIV/AIDS stigma among physicians in training. *AIDS Care*, *28*(3), 376-383. <https://doi.org/doi:10.1080/09540121.2015.1090537>

Varkey, P., Chutka, D. S., & Lesnick, T. G. (2006). The aging game: Improving medical students' attitudes toward caring for the elderly. *Journal of the American Medical Directors Association*, *7*(4), 224-229. <https://doi.org/doi:10.1016/j.jamda.2005.07.009>

Victoroff, K. Z., Williams, K. A., Lalum, & ier, J. (2013). Dental students' reflections on their experiences with a diverse patient population. *Journal of Dental Education*, *77*(8), 982-989.

Villani, M., & Kovess-Masfety, V. (2017). Could a short training intervention modify opinions about mental illness? A case study on French health professionals. *BMC Psychiatry*, *17*(1), 133. <https://doi.org/doi:10.1186/s12888-017-1296-0>

Villegas, C., Flynn, P., Wilson, A., & Hayes, G. (2019). Pilot test of an online bias reduction curriculum for medical students. *Journal of Investigative Medicine*, *67*(1), 190-191.

Volkman, K. (2007). Bates Chair to address health disparities. *Science of Caring*, *19*(2), 5-5.

Wain, T., Sim, M., Bessarab, D., Mak, D., Hayward, C., & Rudd, C. (2016). Engaging Australian Aboriginal narratives to challenge attitudes and create empathy in health care: A methodological perspective. *BMC Medical Education*, *16*(1). <https://doi.org/doi:10.1186/s12909-016-0677-2>

Waite, R., & Calamaro, C. J. (2010). Cultural competence: A systemic challenge to nursing education, knowledge exchange, and the knowledge development process. *Perspectives in Psychiatric Care*, *46*(1), 74-80. <https://doi.org/doi:10.1111/j.1744-6163.2009.00240.x>

Wallace, M., Greiner, P., Grossman, S., Lange, J., & Lippman, D. T. (2006). Development, implementation, and evaluation of a geriatric nurse education program. *Journal of Continuing Education in Nursing*, *37*(5), 214-217. <https://doi.org/doi:10.3928/00220124-20060901-09>

Wardhere, A., Saidy, S. O., Newman, R., & Homayounrooz, F. (2022). Implementing a diversity, equity and inclusion curriculum in an internal medicine residency program. *Journal of General Internal Medicine*, *37*, S290. <https://doi.org/doi:10.1007/s11606-022-07653-8>

Wear, D., Zarconi, J., Aultman, J. M., Chyatte, M. R., & Kumagai, A. K. (2017). Remembering Freddie Gray: Medical education for social justice. *Academic Medicine: Journal of the Association of American Medical Colleges*, *92*(3), 312-317. <https://doi.org/doi:10.1097/ACM.0000000000001355>

Webb, E., & Sergison, M. (2003). Evaluation of cultural competence and antiracism training in child health services. *Archives of Disease in Childhood*, *88*(4), 291-294. <https://doi.org/doi:10.1136/adc.88.4.291>

Webster, D. A. (2009). Addressing nursing students' stigmatizing beliefs toward mental illness. *Journal of Psychosocial Nursing and Mental Health Services*, *47*(10), 34-31. <https://doi.org/doi:10.3928/02793695-20090902-05>

Wechsler, D., Mahlke, C., Bock, T., & Schomerus, G. (2020). Effects of contact-based, short-term anti-stigma training for medical students: Results from a randomized controlled trial. *Neuropsychiatrie*, *34*(2), 66-73. <https://doi.org/doi:10.1007/s40211-020-00337-x>

Weech-Maldonado, R., Dreachslin, J. L., Epané, J. P., Gail, J., Gupta, S., & Wainio, J. A. (2018). Hospital cultural competency as a systematic organizational intervention: Key findings from the national center for healthcare leadership diversity demonstration project. *Health Care Management Review*, *43*(1), 30-41. <https://doi.org/doi:10.1097/HMR.0000000000000128>

Werkhoven, T. (2021). Designing, implementing and evaluating an educational intervention targeting weight bias and fat stereotyping. *Journal of Health Psychology*, *26*(12), 2084-2097. <https://doi.org/doi:10.1177/1359105319901310>

Werkhoven, T., Nordstrom, A. H., & Goodfriend, W. (2022). Achieving a reduction in weight stigma by providing a holistic education intervention targeting nutrition knowledge and bias. In Nordstrom, A., & Goodfriend, W. (Eds*.*), *Innovative stigma and discrimination reduction programs across the world*. Routledge/Taylor & Francis Group.

Wheeler, A., J., Mey, A., Fowler, J. L., Mihala, G., & Kelly, F. (2018). A web-based mental health promotion intervention for pharmacy staff to reduce stigmatising and discriminating attitudes. *Health Promotion Journal of Australia: Official Journal of Australian Association of Health Promotion Professionals*, *29*(3), 328-336. <https://doi.org/doi:10.1002/hpja.33>

Williams, R. M. (2016). Addressing implicit bias: Leading by example. *Academic Medicine*, *91*(2), 163. <https://doi.org/doi:10.1097/ACM.0000000000001039>

Wolsiefer, K., & Stone, J. (2019). Addressing bias in healthcare: Confrontation as a tool for bias reduction and patient and provider self-advocacy. In Mallett, R.K., & Monteith, M.J. (Eds.), *Confronting Prejudice and Discrimination: The Science of Changing Minds and Behaviors*, 275-297. Academic Press. <https://doi.org/10.1016/B978-0-12-814715-3.00013-8>

Woods, C. E., Siegelman, J., Bryant, A., Oyewu, A., Salhi, B., & Heron, S. L. (2015). The implicit associations test: Targeting unconscious physician bias and health care disparities in emergency medicine resident education. *Academic Emergency Medicine*, *22*(5), S447.

Wray, J., Walker, L., & Fell, B. (2008). Student nurses' attitudes to vulnerable groups: A study examining the impact of a social inclusion module. *Nurse Education Today*, *28*(4), 513-520. <https://doi.org/doi:10.1016/j.nedt.2007.09.011>

Wu, S., Li, L., Wu, Z., Liang, L.-J., Cao, H., Yan, Z., & Li, J. (2008). A brief HIV stigma reduction intervention for service providers in China. *AIDS Patient Care and STDs*, *22*(6), 513-520. <https://doi.org/doi:10.1089/apc.2007.0198>

Yiu, J. W., Mak, W. W. S., Ho, W. S., & Chui, Y. Y. (2010). Effectiveness of a knowledge-contact program in improving nursing students' attitudes and emotional competence in serving people living with HIV/AIDS. *Social Science & Medicine*, *71*(1), 38-44. <https://doi.org/doi:10.1016/j.socscimed.2010.02.045>

Yotis, L., Theocharopoulos, C., Fragiadaki, C., & Begioglou, D. (2017). Using playback theatre to address the stigma of mental disorders. *Arts in Psychotherapy*, *55*, 80-84. <https://doi.org/10.1016/j.aip.2017.04.009>

Yousufzai, H., Wang, N., & Abe, R. (2022). Teaching Antiracism to Providers: An Educational Clinical Workshop. *Journal of General Internal Medicine*, *37*, S655. <https://doi.org/doi:10.1007/s11606-022-07653-8>

Zäske, H., Freimüller, L., Wölwer, W., & Gaebel, W. (2014). Antistigma-kompetenz in der psychiatrischen versorgung: Ergebnisse der pilotierung einer berufsgruppenübergreifenden weiterbildung [Anti-stigma competence for mental health professionals: results of a pilot study of a further education programme for people working in psychiatric and psychosocial settings]. *Fortschritte der Neurologie-Psychiatrie, 82*(10), 586–592. <https://doi.org/10.1055/s-0034-1385130>

Zeidan, A., Aysola, J., Khatri, U. G., Shofer, F. S., Conlon, L. W., Scott, K. R., & Mamtani, M. (2018). Implicit bias training in emergency medicine residency: There's a right answer. *Academic Emergency Medicine*, *25*, S230-S231.

Zhou, Y., Purkiss, J., Juneja, M., Greely, J., Beasley, A., & Gill, A. (2022). Dataset: Knowledge and attitude retention following an implicit bias classroom workshop. *F1000Research*, *11*. <https://doi.org/doi:10.12688/f1000research.74442.1>
